# Evaluating the protonation state of the catalytic Cys25 in cruzain cysteine protease: A target for Chagas disease

**DOI:** 10.1002/pro.70283

**Published:** 2025-09-17

**Authors:** Clauber H. S. da Costa, Vinícius Bonatto, Hemillin Brenda Teixeira Santos, Carlos Gabriel da Silva de Souza, Carlos A. Montanari, Munir S. Skaf, F. Javier Luque, Jerônimo Lameira

**Affiliations:** ^1^ Institute of Chemistry and Center for Computing in Engineering & Sciences University of Campinas – UNICAMP Campinas Brazil; ^2^ São Carlos Institute of Chemistry Grupo de Química Medicinal do Instituto de Química de São Carlos da Universidade de São Paulo São Carlos Brazil; ^3^ Laboratory of Computer Modeling of Molecular Biosystems (CompMBio) Federal University of Pará Belém Brazil; ^4^ Department of Nutrition, Food Science and Gastronomy, Faculty of Pharmacy and Food Science‐Campus Torribera, Institute of Biomedicina (IBUB) and Institute of Theoretical and Computational Chemistry (IQTCUB) University of Barcelona Santa Coloma de Gramenet Spain

**Keywords:** cruzain, Cys25, molecular dynamics simulations, protonation state, *Trypanosoma cruzi*

## Abstract

Cruzain (Cz), the major cysteine protease of *Trypanosoma cruzi*, the etiological agent of Chagas disease, employs Cys25 as its catalytic nucleophile, enabling peptide bond hydrolysis via nucleophilic attack on the carbonyl carbon of substrates. The pKa of Cys25 can be modulated by the local environment in the free enzyme or upon formation of pre‐reactive complexes with substrates or inhibitors. Here, we employ molecular dynamics (MD) simulations, free energy calculations, and constant‐pH simulations with explicit solvent to investigate the protonation state of Cys25 in the apoenzyme and in complexes with either a substrate mimic (Ac‐Ala‐Ala‐Ala‐Gly‐Ala‐OCH₃) or the covalent inhibitor K777 (*N*‐methyl‐piperazine‐phenylalanyl‐homophenylalanyl‐vinylsulfone‐phenyl). The simulations consistently support the presence of a neutral Cys25/His162 dyad across all states examined. Binding of either substrate or inhibitor reinforces a weak hydrogen bond between Cys25 and His162 but does not substantially perturb the dyad's protonation state. These findings suggest that cruzain's catalytic dyad remains predominantly neutral in the apo and bound forms, with the formation of a CysS^−^/HisH^+^ ion pair likely occurring as an early event during catalysis or covalent inhibition. These insights have important implications for understanding the catalytic mechanism and for the rational design of cruzain‐targeted therapeutics.

## 1 INTRODUCTION

Cysteine proteases emerge as potential targets in the quest for innovative therapeutics for a broad spectrum of human diseases (Cianni et al., 2019). Many pathological conditions in humans are associated with the malfunctioning of these enzymes, making them attractive targets for drug discovery (Liu et al., 2018; Mohamed & Sloane, 2006; Patel et al., 2018; Turk, 2006). Additionally, cysteine proteases from parasites and viruses are an appealing approach for targeting infectious diseases (Agbowuro et al., 2018; Ettari et al., 2013; Mckerrow, 2018; Pandey & Dixit, 2012; Xiong et al., 2021). Among these proteases, cruzain (Cz) stands out as a prime example in *Trypanosoma cruzi* (Cazzulo et al., 1990; Eakin et al., 1993; Otto & Schirmeister, 1997; Verma et al., 2016), where it plays a crucial role in the development and survival of the parasite inside and outside the host cells. In particular, Cz garners attention as an interesting target for the treatment of Chagas disease, a nomenclature attributed to Carlos Chagas, who first elucidated this disease in 1909 (Chagas, 1909). Considering that this neglected disease is gaining increasing impact in endemic and even non‐endemic countries, efforts to cure and eradicate Chagas disease are an urgent medical need (Lidani et al., 2019).

**FIGURE 1:**
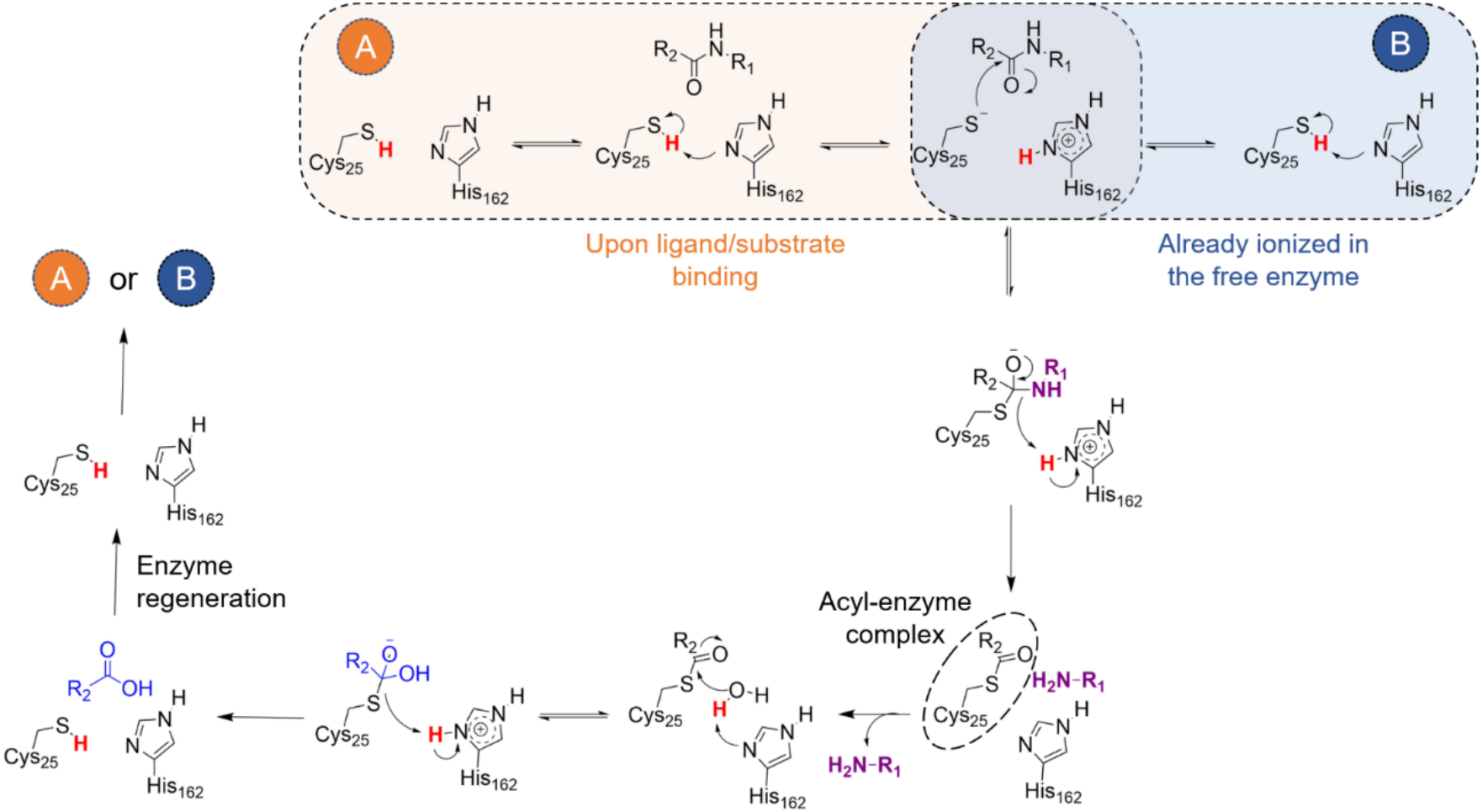
Reaction mechanism catalyzed by cruzain in a complex with substrate or covalent inhibitor. The ion pair can be formed before or upon ligand binding.

Cysteine proteases feature a catalytic Cys/His dyad that orchestrates protein hydrolysis via a nucleophilic attack directed at the carbonyl group of scissile peptide bonds (Coulombe et al., 1996; Verma et al., 2016). Often, the catalytic dyad is also referred to as a catalytic triad with an additional Asn residue. However, this latter residue is sometimes absent from the active site, and its function is compensated by a water molecule (Grigorenko et al., 2023; Kneller et al., 2020). One intriguing question regarding the catalytic mechanism of cysteine proteases involves the formation of the Cys/His ion pair, which can exist in the apo enzyme (B pathway in Figure 1) or be formed after binding of the ligand/substrate (A pathway in Figure 1) (Creighton et al., 1980; Keillor & Brown, 1992; Wei et al., 2013). Figure 1 illustrates the two potential routes of the ion pair formation in the context of the reaction mechanism of Cz against a substrate or covalent inhibitor.

Employing the empirical valence bond model, Warshel and coworkers determined that the Cys → His proton transfer in Cz is energetically more favorable in the absence of substrate within the active site (B pathway in Figure 1) (Oanca et al., 2020). However, they stated that the presence of water molecules could exert a significant influence on the proton transfer energetics. Hence, the thermodynamic cycle employed in their study may be subject to modification, potentially resulting in alterations to the calculated free energy changes. In this regard, the results obtained for a variety of cysteine proteases disclose the existence of distinct, even conflicting, insights into the ion pair formation. For instance, studies on papain support the formation of the ion pair prior to ligand/substrate binding (Lewis et al., 1976). A similar finding was made for caricain and ficin, as the p*K*a of the catalytic Cys was estimated to be 2.9 and 2.5 (Harris & Turner, 2002), respectively, in the free enzyme. For human cathepsin B, a structurally Cz‐related enzyme, the presence of the ion pair in the apo enzyme was also suggested in previous studies (Hasnain et al., 1992). Additionally, the analysis of electrostatic properties in the catalytic site of papain has shown that the formation of the Cys25^−^/His159^+^ ion pair is favored by a net electric field aligned along the S(Cys25)‐Nδ(His159) axis (Dardenne et al., 2003). The existence of the ion pair was supported by different experimental (Creighton et al., 1980; Lewis et al., 1976; Lewis et al., 1981) and computational studies for cysteine proteases (Wei et al., 2013). In contrast to the previous studies, Zhai and Meek demonstrated the neutral state of the catalytic Cys and His in the unbound Cz through solvent kinetic isotope effects (Zhai & Meek, 2018). Based on the pH‐rate profiles, they assigned p*K*a values of 9.7 and 6.6 for Cys and His, respectively, in the apo enzyme and proposed that the neutral dyad seemingly undergoes proton transfer only upon substrate binding (A pathway in Figure 1) (Zhai & Meek, 2018). Similar observations were made for other cysteine proteases, as noted by Villamil and colleagues (Villamil et al., 2012). Furthermore, molecular simulations of the main protease (M^pro^) of severe acute respiratory syndrome coronavirus 2 (SARS‐CoV‐2) indicated that the catalytic Cys145 assumes a neutral state in the free enzyme and may change to its pre‐reactive complex with different inhibitors (Pavlova et al., 2021). Nevertheless, neutron crystallography studies of the M^pro^ free enzyme suggested that the Cys^−^/His^+^ ion pair is present in the apo enzyme (Kneller et al., 2021).

Overall, the chemical nature of the catalytic dyad either before or after ligand binding is still a challenging question that may exhibit a large dependence on the specific features of cysteine proteases. In this context, the determination of the p*K*a of the Cys residue holds central significance for elucidating the ionization state of the catalytic dyad of the enzyme. The intrinsic p*K*a of Cys is approximately 8.6 (Roos et al., 2012) and, in general, the p*K*a of Cys in peptides ranges from 7.4 to 9.1 (Bulaj et al., 1998). Accordingly, this residue mainly populates the neutral form at pH = 7.0. However, the protein microenvironment and exposure to the solvent can drastically shift the p*K*a of a given Cys, resulting in values as low as 2.9 (Bonatto et al., 2023; Lohse et al., 1997; Zhang & Dixon, 1993).

In this context, the prediction of p*K*a values in protein‐ligand complexes is of paramount importance, especially in the realm of structure‐based drug design targeting reactive cysteines. Many methods can be used as p*K*a predictors. For instance, Wei and coworkers reported a benchmark study for seven high‐throughput p*K*a prediction methods (Wei et al., 2023). While computationally fast and efficient methods mainly pertain to macroscopic physics‐based (e.g., H++ and DelPhiPKa) or empirical (e.g., PROPKA3) categories, attaining chemical accuracy in the p*K*a requires more robust methods, such as microscopic physics‐based techniques, including the use of all‐atom molecular dynamics (MD) simulations to capture the dynamical motions of the protein. Hofer et al. have reported the use of constant pH molecular dynamics (CpHMD) simulations to predict the p*K*a of the catalytic Cys of papain (Hofer et al., 2020). To obtain the titration curve of Cys25, they used different implicit solvent models and GB‐radii of 2 Å for the sulfur atom (Liu et al., 2019). However, they were not able to reproduce the reported experimental pKa of 3.3, since the predicted value was 8.7. Recently, Awoonor‐Williams and coworkers benchmarked in silico tools to predict the p*K*a of Cys residues using a wide range of methods (Awoonor‐Williams et al., 2023). For CpHMD simulations, they used both implicit and explicit solvent models, but the results were not satisfactory in any case. They also tried to employ thermodynamic integration (TI) calculations, but no significant improvement was obtained in comparison with the CpHMD results. In such cases, the authors used the Amber ff99SB and ff14SB force fields and one plausible reason for the prediction failure was attributed to the usage of identical sigma/epsilon parameters in the Lennard‐Jones description adopted for thiol and thiolate forms. Also, it was not clear how structural water molecules were treated as they can be crucial for stabilizing the ion pair formation.

Herein, we have used MD simulations in conjunction with CpHMD (Donnini et al., 2011; Lee et al., 2014; Mongan et al., 2004; Swails et al., 2014) and TI calculations, both with explicit solvent, to evaluate the protonation state of the catalytic Cys25 of Cz. To this end, three simulation systems have been considered: the ligand‐free Cz and the enzyme in complex with both its substrate analog (Ac‐Ala‐Ala‐Ala‐Gly‐Ala‐OCH_3_) and the vinyl sulfone‐derived inhibitor K777 (N‐methyl‐piperazine‐phenylalanyl‐homophenylalanyl‐vinylsulfone phenyl; also known as K11777), which is an irreversible inhibitor. The results are used to discuss the merits of the two computational techniques for predicting the protonation state of Cys25 and His162 in Cz. In this sense, we aim to provide molecular details about the protonation states of the catalytic residues in Cz, particularly Cys25. The results are examined in light of previous experimental data that determined the p*K*a of the apo enzyme, and the comparative analysis will be used to discuss the reliability of computational techniques used to determine the p*K*a of amino acids in the active site of enzymes. This is critical for guiding drug discovery efforts, particularly in the design of new cysteine protease inhibitors.

## 2 METHODS

### 2.1 The system setup

**FIGURE 2:**
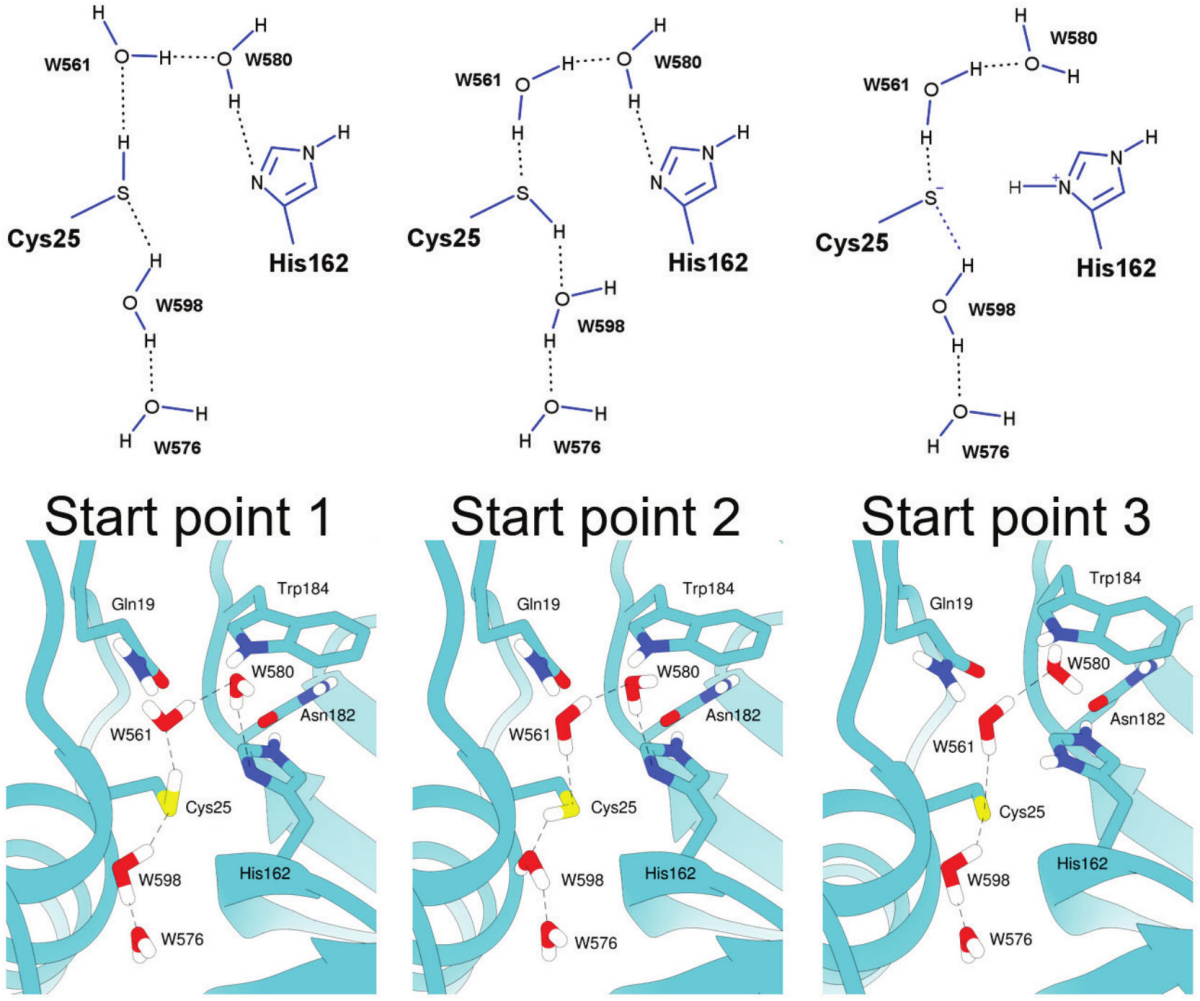
Initial structures used for MD simulations: Start point 1—Cys25 as HB acceptor from W598 and HB donor to W561; Start Point 2—Cys25 as HB donor to W598 and HB acceptor from W561; Start Point 3—Presence of the CysS^−^/HisH^+^ ion pair. Systems defined from the crystal structure of Cz (PDB code: 6N3S) (da Barbosa Silva et al., 2019).

The initial atomic coordinates for Cz were obtained from the Protein Data Bank (PDB) under the code 6N3S (da Barbosa Silva et al., 2019). The p*K*a of all residues, except for His162 and Cys25, were determined using the PDB2PQR server (Jurrus et al., 2018). The Amber18 package (Case et al., 2018) was used in conjunction with the ff14SB (Maier et al., 2015) force field for the protein and TIP3P (Jorgensen et al., 1983) water molecules using the *pmemd.cuda* module. The missing hydrogen atoms of the protein were added using the *tleap* module as implemented in AmberTools18. Each system was solvated in a truncated octahedral cell, extending 10 Å outside the protein on each side. Initially, each solvated complex was energy‐minimized (7500 steps of steepest descent and 7500 steps of conjugate gradient approach), and then gradually heated from 100 K to 300 K for 500 ps of MD simulations with a constraint of 100 kcal/mol·Å^2^ on the protein heavy atoms. Next, each system was relaxed for 200 ps in five stages where the constraints were gradually eliminated. Finally, for ligand‐free Cz, we performed 3 replicas of 300 ns each, considering two possible start points (see Figure 2). In these replicas, we explored different starting points for ligand‐free Cz (see Figure 2). In all cases, a nonbonded cutoff of 8 Å was used in conjunction with the particle mesh Ewald (PME) approach for the treatment of long‐range Coulomb forces. The SHAKE method was applied for all hydrogen bonds during the simulations.

MD simulations were performed considering three starting systems (see Figure 2), which encompass distinct hydrogen bond (HB) arrangements involving crystallographic water molecules within the active site of Cz. In start point 1 and 2, Cys25 is acting as HB acceptor and donor, respectively, and in start point 3, Cys25 is deprotonated and His162 is positively charged. Note that suitable constraints were used to maintain key HB distances (see Figure 2), which were gradually reduced during the equilibration stage performed for these systems. The choice of these starting systems permits assessing the stability of distinct HB patterns, especially the distance between the thiol hydrogen (HG) of Cys25 and the Nδ (ND1) of His162 and facilitates the exploration of the energy landscapes for these configurations. This analysis aims to determine if the HBs formed by these atoms remain stable, even when both residues initially interact with water molecules at the beginning of the MD simulations.

### 2.2 Free energy landscape

In this study, the Bio3D package (Grant et al., 2006) was employed for conducting principal component analysis (PCA). The principal components (PCs) were derived through the diagonalization of the positional covariance matrix, calculated using the cartesian coordinates of the Cα atoms for the ensemble of aligned Cz structures. In order to prevent an underestimation of atomic displacements, a repetitive superposition method was executed prior to PCA. This involved iteratively excluding residues with the most significant positional variations in each round, ultimately retaining only the unchanged “core” residues (Grant et al., 2006).

The analysis of the free energy landscape (FEL) was performed using the two first principal components (PC1 and PC2) derived from the PCA. The Gibbs free energy associated with PC1 and PC2 is denoted as $∆G\left({\mathrm{PC}}_{s}\right)$ (Equation 1).

(1) $$∆G\left({\mathrm{PC}}_{s}\right)=-k_{B}T\left[\mathrm{ln\rho}\left({\mathrm{PC}}_{1},{\mathrm{PC}}_{2}\right)-\ln\rho_{\max}\right]$$

This expression is based on the probability distribution derived from the MD trajectories, where $k_{B}$ is the Boltzmann constant, T is the temperature, and $\rho_{\max}$ correspond to the probability value corresponding to the most significant conformation, which is subtracted from the free energy to approximate it to 0 (Karamzadeh et al., 2017; Papaleo et al., 2009).

To investigate conformations close to the native structure, two‐dimensional FEL values were extracted from the probability distributions of both PC1 and PC2 across all the studied systems. The FEL plot and the conformational states identified in the Cz structure's energy landscape were generated using the *cpptraj* module within the Amber18 package. This analysis was also used to identify the primary structures contributing to the first (PC1) and second (PC2) components. Solely the atomic coordinates of the Cα atoms of Cz were utilized for creating the PCA plots to ensure a uniform atom representation of both bound and unbound states. Additionally, per‐residue Cα atomic fluctuations and B‐factors were computed for each starting conformation using the *cpptraj* module in AmberTools18.

### 2.3 Molecular docking and MD simulations of protein‐ligand complexes

Five minima (A, B, C, D and E) identified from the FEL were used to perform noncovalent docking of Ac‐Ala‐Ala‐Ala‐Gly‐Ala‐OCH_3_ and K777 in the active site with Molegro Virtual Docker (MVD) (Thomsen & Christensen, 2006). The parameters used for docking were X = 57.8, Y = 0.5, and Z = 6.7, with a radius = 9.0 Å, using the Scoring Function MolDock Score with the MolDock SE Algorithm, with a number of runs equal to 10 and generating a maximum of 50 conformational poses in the active site. More information about the scoring function used here can be found elsewhere (Thomsen & Christensen, 2006).

It is worth noting that the docking protocol was validated using the crystallographic structure of Cz bound to K777 (PDB ID 2OZ2) (Kerr et al., 2009) which was used as a control in our molecular docking study (Brak et al., 2010). The alignment of this structure (PDB ID 2OZ2) with the ligand‐free enzyme (PDB ID 6N3S) showed that the structural features of the residues that shape the binding pocket are almost identical, revealing that binding of the inhibitor occurs without relevant alterations in the side chain of the residues in the binding pocket. In this regard, the results of molecular docking calculations show that the substrate (Ac‐Ala‐Ala‐Ala‐Gly‐Ala‐OCH_3_) exhibits a structural arrangement very similar to K777 (Figure S1). In addition to the crystallographic structure, molecular docking of both the substrate and K777 was extended to each minimum found during the simulation of the apo systems (Figure S1; scoring values given in Table S1). Figure S1 shows the ligand poses obtained for the minima A, B, C, D, and E and the configuration determined using the crystallographic structure of the Cz‐K777 complex. The results indicated that docking calculations led to a proper alignment with the ligand's pose observed in the X‐ray crystallographic structure.

For the Cz‐K777 and Cz‐substrate complexes, we performed five replicas of 300 ns each (A, B, C, D, and E), resulting in a total of 3000 ns of MD simulation. The Amber18 MD package (Case et al., 2018) with ff14SB (Maier et al., 2015) (protein), which was shown to provide an improved description of the conformational preferences of backbone and side chains, GAFF (Wang et al., 2004) (ligands) and TIP3P (Jorgensen et al., 1983) (water molecules) parameter sets were used for simulations of the protein‐ligand complexes. The optimized structures and partial charges for the inhibitors were obtained utilizing the RESP method at HF/6‐31G* quantum mechanical level using the Gaussian09 program. The MD simulations of the protein‐ligand complexes were performed using the protocol described above for the free Cz enzyme.

### 2.4 Constant pH simulations in explicit solvent

CpHMD calculations were used for predicting the effective p*K*a of Cys25 and His162 (Donnini et al., 2011; Lee et al., 2014; Mongan et al., 2004; Swails et al., 2014). In this study, CpHMD simulations were run in explicit solvent using discrete protonation states. This method involves standard MD simulations being propagated in explicit solvent, followed by protonation state changes being attempted in GB implicit solvent at fixed intervals (Swails et al., 2014). CpHMD simulations were performed using the *pmemd.cuda* module of AMBER18 (Case et al., 2018) CpHMD calculations were conducted for the following systems: (i) a Cys residue in the solvated peptide Gly‐Ser‐Cys‐Trp‐Ala, conveniently modified at N‐ and C‐termini with capping groups, which correspond to the neighboring residues of Cys25 in Cz, (ii) the structure of the ligand‐free Cz, and (iii) the complexes of Cz with substrate and K777.

In the simulations accounting for the protein environment, the p*K*a of Cys25 was assessed under two scenarios: one with neutral His162 and another with positively charged His162. The CpHMD calculations were performed using the final frame of the MD simulations of the complexes from replicas A, B, C, and D as starting points. Eight replicas were performed for each CpHMD calculation for Cz‐K777 and Cz‐substrate complexes and ligand‐free Cz, where the p*K*a at each time step was computed from a running average of the deprotonation fraction, using 5 ns per window at each pH value. Taking into account that the simulations were performed for Cz with His162 in its neutral and positively charged forms, we have obtained a total of 3000 ns in the CpHMD calculations.

Simulations encompassed pH values ranging from 0 to 14. CpHMD calculations can be analyzed in a fashion entirely analogous to that used for experiments that give information about the protonation changes as a function of pH for individual side chains. As long as the protonation fraction is a monotonic function of pH, the p*K*a of a side chain can be defined as the pH value for which the protonated and deprotonated populations are equal. The special case of an ideal titratable group having no interactions with other titratable groups has a sigmoidal titration curve, and the behavior is characterized by the Henderson‐Hasselbalch equation.

The deprotonation fraction (df; Equation 2) and pH for each simulation and each window of the running averages was fitted to the modified Hill equation using the Levenberg–Marquardt nonlinear least‐squares algorithm implemented in SciPy to compute the p*K*a and Hill coefficient (*n*). All p*K*a values reported for titratable residues in this paper correspond to the value computed by fitting df from the simulations at every pH over the specified time interval (Swails et al., 2014).

(2) $$\mathrm{df}=\frac{1}{1+10^{n\left(\mathrm{pKa}-\mathrm{pH}\right)}}$$

### 2.5 Thermodynamics integration

**FIGURE 3:**
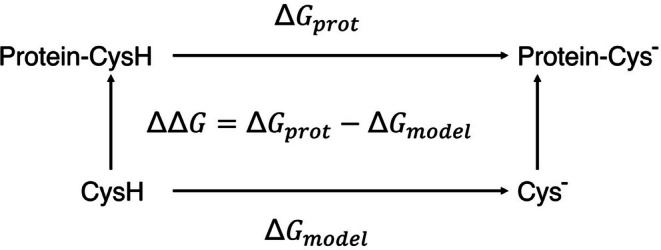
Thermodynamic cycle used to analyze p*K*a shifts. The transformations were carried out in solution and in a protein environment. The double free energy difference between the two legs is calculated as $\mathrm{\Delta \Delta G}={\mathrm{\Delta G}}_{\text{prot}}-{\mathrm{\Delta G}}_{\text{model}}$.

Thermodynamic integration (TI) was used to calculate the p*K*a shifts (Δp*K*a; Equation 3) using the approach proposed by Warshel and co‐workers (Warshel et al., 1986)

(3) $$∆\mathrm{pKa}=\frac{1}{2.303k_{B}T}\left(∆∆G\right)$$

where *k*_*B*_ is the Boltzmann constant, T is the temperature used in the simulations, and *ΔΔG* is the difference in the free energy of the enzyme considering the change of the thiol group to thiolate anion of Cys25 (Figure 3).

To obtain *ΔΔG*, TI calculations involved a total number of 11 windows corresponding to λ values of 0.0, 0.1, 0.2, 0.3, 0.4, 0.5, 0.6, 0.7, 0.8, 0.9, and 1.0. Note that the neutral Cys was defined as *λ* = 0.0, and the thiolate anion was defined as *λ* = 1.0. For each window, 2 ns MD simulations were run, one for relaxation and one for production. TI calculations were obtained following the protocol described in (Simonson et al., 2004) using four replicas for apo and eight replicas for complexes (see Supporting Information). In these simulations, the protonated Cys was gradually transformed to thiolate by modifying the energy function *U* as described below:

$$U\left(\lambda \right)=\left(1-\lambda \right)U_{A}+\lambda U_{B}$$

where *A* represents the initial state, *B* the final state, and *λ* is the coupling parameter.

Furthermore, we employed the Minimum‐Distance Distribution Function (MDDF) approach to characterize protein‐solvent interactions using the ComplexMixtures.jl package (Martínez, 2022).

## 3 RESULTS AND DISCUSSION

The 300‐ns MD simulations on the ligand‐free Cz were used to examine the structural features of the active site for three systems corresponding to distinct protonation states of Cys25 and His162 (Figure 2). The positional root‐mean‐square deviation (RMSD) of the backbone confirmed the structural stability of the protein fold (RMSD values close to 1 Å; Figure S2). The distance from the Cys25 sulfur atom (SG) to the His162 imidazole nitrogen atom (ND1) is, on average, 3.43 Å for the neutral residues (Table 1). These results agree with the values found in the apo x‐ray structure (3.3 Å in PDB ID 6N3S, which compares with distances of 3.3–3.7 Å in ligand‐bound structures; da Barbosa Silva et al., 2019). Table 1 also shows that the average distance from the Cys25 thiol hydrogen and ND1 is close to 2.7 Å (Figure S3), thus reflecting a favorable configuration for proton transfer from Cys25 to His162 (Zhai & Meek, 2018). In contrast, the S(Cys25)⋯Nδ(His162) distance is reduced to 3.08 Å in the free enzyme with the Cys25^−^/His162^+^ ion pair. In this regard, MD simulations suggest that His162 and Cys25 have neutral charges in the free enzyme.

**TABLE 1:** Average distance (Å) for Cys25– His162 interaction in the free form of Cz and in the complex with K777 and substrate (standard deviation in parentheses). The S(Cys25)⋯Nδ(His162) distance found in x‐ray structures of the unbound (6N3S; da Barbosa Silva et al., 2019), and ligand‐bound (1AIM [Gillmor et al., 1997], 2AIM [Gillmor et al., 1997], 2OZ2 [Kerr et al., 2009], 4KLB [Wiggers et al., 2013], and 6UX6 [Zhai et al., 2020]) species are also indicated.

Average distances (Å)			
	SG(Cys25)⋯Nδ(His162)	HG(Cys25)⋯Nδ(His162)	SG(Cys25)⋯HN_δ_(His162)
Free form (Cruzain—6N3S)			
Start point 1	3.43 (0.23)	2.71 (0.73)	
Start point 2	3.43 (0.23)	2.70 (0.75)	
Start point 3	3.08 (0.16)		2.16 (0.26)
Bound form (Complex K777)			
Minimum A	3.35 (0.19)	2.39 (0.54)	
Minimum B	3.34 (0.19)	2.38 (0.40)	
Minimum C	3.32 (0.17)	2.31 (0.40)	
Minimum D	3.32 (0.18)	2.32 (0.39)	
Minimum E	3.09 (0.13)		2.25 (0.19)
Bound form (complex substrate)			
Minimum A	3.37 (0.20)	2.55 (0.56)	
Minimum B	3.38 (0.20)	2.58 (0.58)	
Minimum C	3.38 (0.20)	2.55 (0.57)	
Minimum D	3.38 (0.20)	2.58 (0.60)	
Minimum E	3.02 (0.10)		2.07 (0.13)
Experimental			
6N3S	3.31		
1AIM	3.71		
2AIM	3.75		
**2OZ2**	3.58		
4KLB	3.34		
6UX6	3.68		

Since PCA describes the largest amplitude protein motions during a simulation, the bi‐dimensional FEL was obtained taking into account the projections of the first principal components (PC1, PC2 and PC3), which account for 53.4%, 42.8%, and 44.2%, respectively, of the structural variance sampled in the simulations.

**FIGURE 4:**
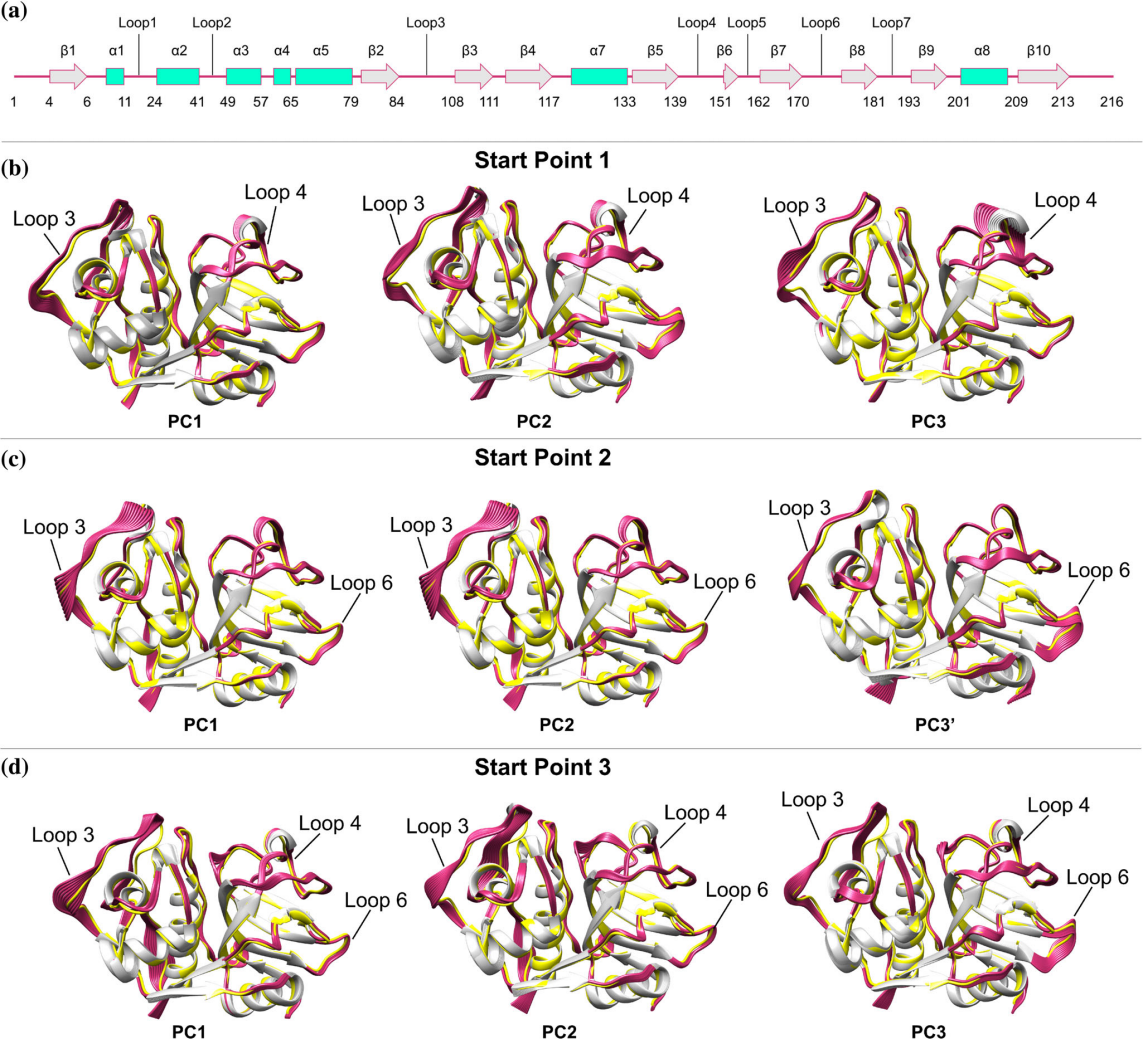
(a) Distribution of secondary structural elements along the sequence of Cz. (b–d) Major conformation motions for the backbone of Cz encountered from the PCA analysis of the simulations started for Start points (b) 1, (c) 2, and (d) 3. The backbone of apo Cz in the x‐ray structure (6NS3) is shown as yellow cartoon.

The PCA results demonstrate that the main conformational changes in the free Cz are associated with loop3, which links strands β2 and β3, together with loop 4, which connects strands β5 and β6, and loop 6, which is found between β7 and β8 (Figure 4). For start point 1, the conformational changes mainly affect loops 3, 4, and 6 compared with the x‐ray structure of Cz (shown in yellow in Figure 4). The result for start point 2 highlights the motions of loops 3 and 6, whereas the analysis of start point 3 primarily discloses the conformational flexibility of loops 3 and 4, and at lesser extent loop 6.

**FIGURE 5:**
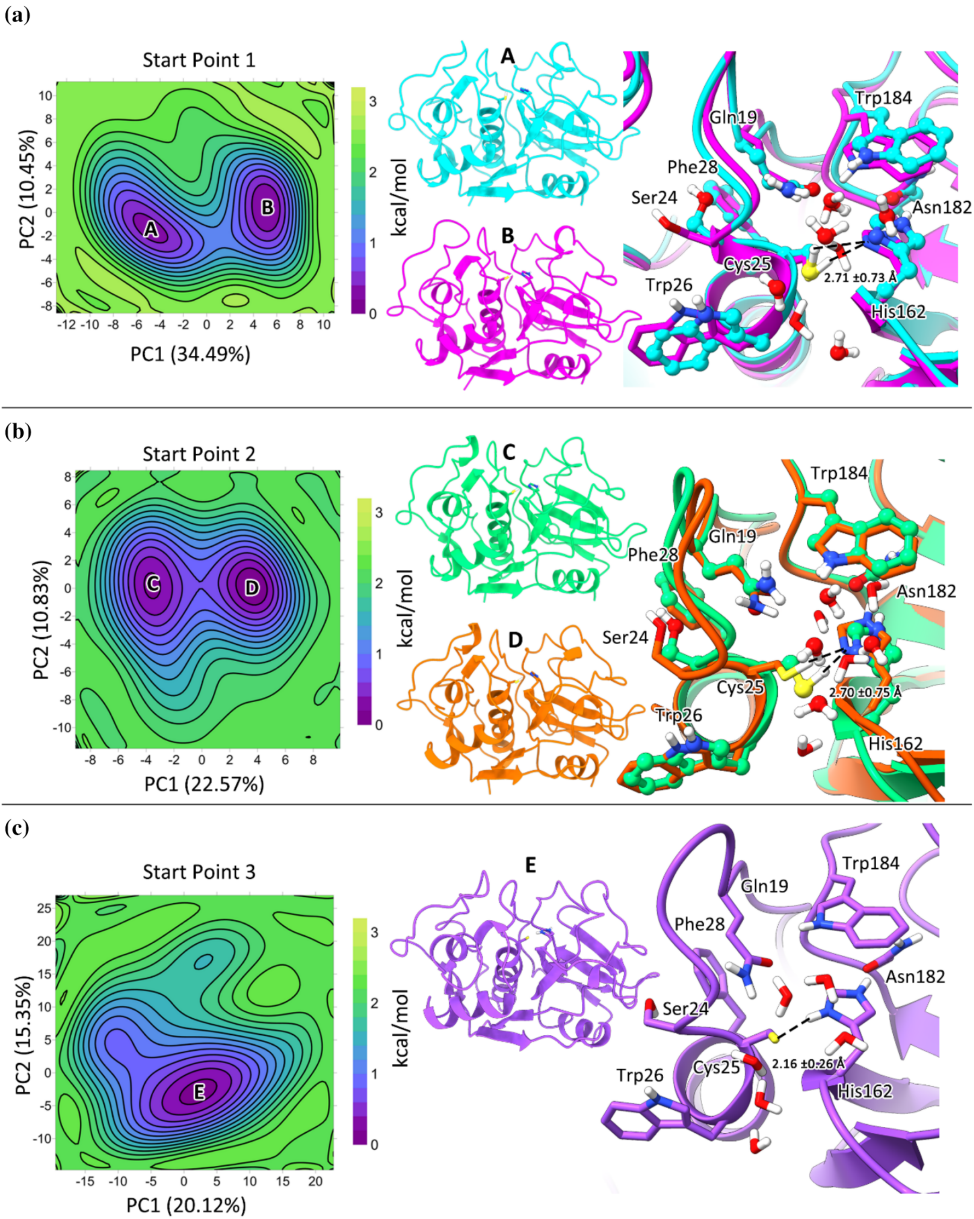
FEL analysis of ligand‐free Cz considering three starting points: (a) Start Point 1 with Cys25 as HB acceptor from W598 and HB donor to W561. This FEL shows two distinct minima: Minimum A (cyan) and minimum B (purple). In minimum A, Cys25 is positioned to form a HB with a water molecule, whereas in minimum B Cys25 forms a weak HBond with His162. (b) Start Point 2 with Cys25 as HB donor to W598 and HB acceptor from W561. It shows two distinct minima: Minimum C (green) and minimum D (orange), where Cys25 forms a HB with His162 in both cases. (c) Start Point 3 with CysS^−^/HisH^+^ ion pair shows a single minimum (minimum E, violet), in which Cys25 forms HB with His162.

The projection of the sampled trajectories onto the two main principal components (PC1 and PC2) were used to examine the FEL plots, since the contribution of the third principal component (PC3) is less than 10% (Figure 5; see also Figure S4 for the projections involving PC3). Two minima can be identified from the FEL analysis for each replica in the case of start points 1 and 2 (see Figure 5a,b). One minima Cys25 is positioned to form a weak HB with His162 and another minima Cys25 may form a HB with a water molecule. In contrast, the analysis performed for start point 3 displayed a single minimum in the FEL (Figure 5c).

The B‐factor results are consistent with the PCA, indicating that the regions of highest flexibility in Start Points 1, 2, and 3 (Figure S5) are primarily located in loop 2 (between α2 and α3), loop 3 (connecting β2 and β3), loop 4 (between β5 and β6), and loop 6 (between β8 and β9). The experimental B‐factor data from PDB structures 6N3S and 6UX6 (Figures S5d and S5e) support these findings, demonstrating that the simulations reproduce the dynamic behavior of these flexible regions and thereby validate our computational approach.

In order to analyze the involvement of water molecules in the HB network with the catalytic Cys in the ligand‐free trajectories, we have we employed the MDDF approach (Martínez, 2022) to characterize protein‐solvent interactions. The Global protein‐water MDDFs show conserved hydration shells, where the function g^md^(r), see Figure S6, represents the probability distribution of the shortest distance between any atom of the solute (here, the protein, including the catalytic residues Cys25 and His162) and water molecules sampled throughout the MD trajectories. Figure S6a depicts the spatial organization of water molecules in the free cruzain form (start points 1, 2, and 3). The sharp first two peaks at approximately 1.8 and 2.8 Å, indicative of water–protein hydrogen bonding, reveal an ordered solvent arrangement consistent with the presence of structural water molecules previously identified in the crystallographic structure (Martínez & Shimizu, 2017).

The three profiles are nearly superimposable, indicating that, regardless of the initial conformational state, the catalytic site converges toward a conserved solvation architecture. This observation highlights the robustness of the aqueous microenvironment surrounding the catalytic dyad across the different systems, including the neutral (Start Points 1 and 2) and ionic (Start Point 3) states. The individual MDDFs computed for Cys25 and His162 exhibit coordination densities ranging from 0.2 to 0.8, with His162 consistently showing a higher propensity to coordinate water molecules throughout the simulations (Figures S2c,d and S6b).

To further dissect these interactions, we performed a per‐residue decomposition of the solute‐solvent MDDFs. Figure S6e–g displays the contributions of the main active site residues, shown as contour plots representing the minimum‐distance solvent density. The patterns observed across Start Points 1, 2, and 3 are highly consistent, with Asp140 (D140), Asp161 (D161), His162, and Cys25 exhibiting prominent water coordination within the buried active site. Additional solvent exposure was detected for Asn182, Ser183, and Trp184, which are closer to the protein surface (Figure S6). Together, these results demonstrate that water‐based networks formed at the beginning of the simulations persist throughout the trajectories, effectively coordinating and stabilizing the catalytic dyad. This behavior is consistent with the energy minima captured in Figure 5 and reinforces the functional relevance that may be played by structured water molecules in the protonation dynamics of the catalytic site. Particularly, it modulates the formation or suppression of the Cys^−^/His^+^ ion pair.

**FIGURE 6:**
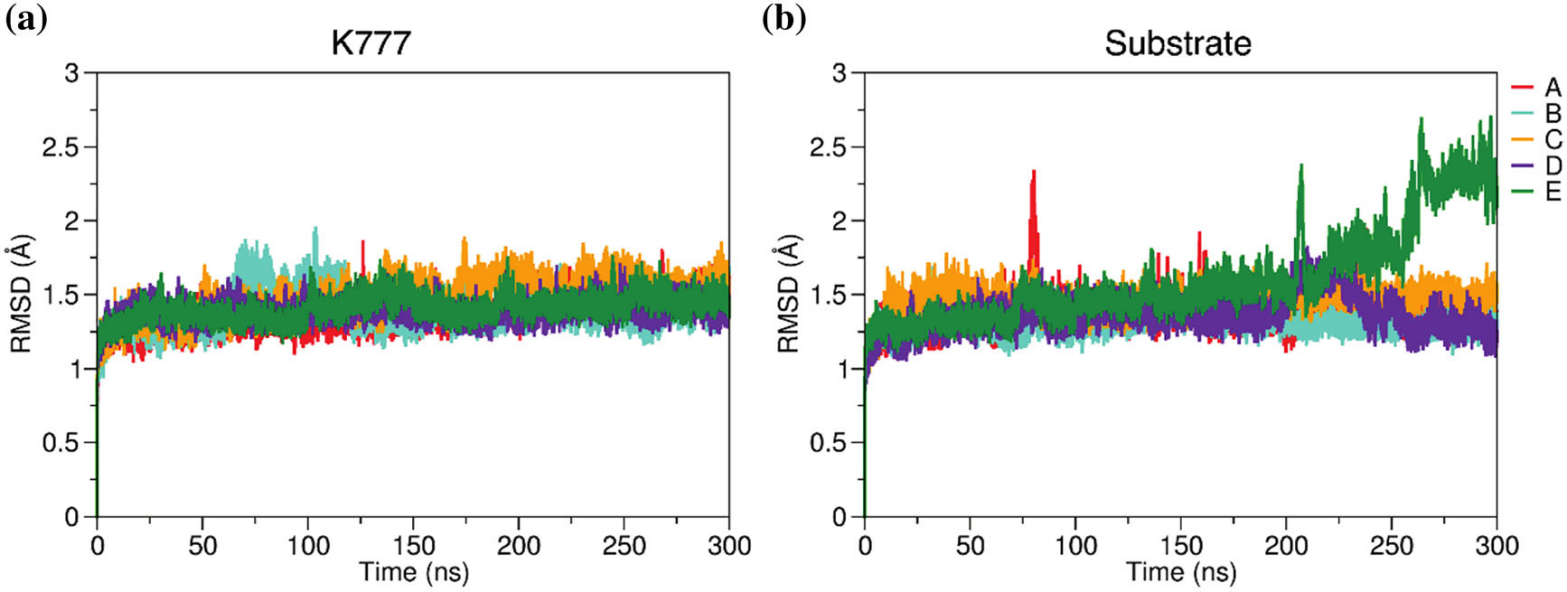
The RMSD results for (a) Cz‐K777 and (b) Cz‐substrate complexes in all replicas for the minimums: A–E along 300 ns of MD simulations.

To gain a detailed understanding of the key interactions in the active site of Cz, MD simulations were conducted for Cz in complex with substrate and K777. To this end, the bi‐dimensional free energy landscape (FEL) was used to generate protein templates for docking of both substrate (Ac‐Ala‐Ala‐Ala‐Gly‐Ala‐OCH_3_; a small peptide previously used in a computational investigation of Cz's catalytic mechanism [Simonson et al., 2004]) and inhibitor (K777). In particular, the minimum‐energy structures A–E (as shown in Figure 5) were selected as templates for docking calculations. The docking outcomes are depicted in Figure S1. In general, the results were able to reproduce the experimental pose found in the x‐ray complex with K777 (PDB code 2OZ2), thus giving confidence to the predicted structure of the ligand‐bound complexes (Kerr et al., 2009). Next, these structures were used for MD simulations of the holo species (300 ns). It is worth mentioning that simulations were performed for two independent replicas using the set of minima (A–E) identified from the analysis of the FEL plots as starting points. The RMSD results during 300 ns of MD simulations show that the Cz‐K777 and Cz‐substrate complexes are stable in all replicas (Figure 6), but for the simulation performed for the complex with the substrate in minimum E (see below).

To investigate the increase in RMSD values for the complex in the minimum energy state (E), we analyzed the non‐bonded interaction energies (electrostatic and van der Waals) between the substrate and the protein over the entire 300 ns simulation. Our findings, presented in Figure S7, provide strong evidence that the observed RMSD increase is not due to a force field issue, but rather reflects the dynamic behavior of the substrate within the binding pocket. Figure S7 shows that both electrostatic and van der Waals energies fluctuate around a stable mean throughout the simulation. There is no systematic drift toward less favorable values, indicating that the interactions remain consistently strong. The probability density plots in Figure S7 reinforce this conclusion: both energy distributions are unimodal and narrow, suggesting that the complex samples conformations within a single, well‐defined energetic state.

The MD simulations revealed stable trajectories for the inhibitor K777 bound to Cz (Figure S8). During the MD simulation, the binding of K777 was favored through the formation of a HB interaction with the main chain carbonyl oxygen of Asp161 and atom N4 of K777, maintaining an average distance of approximately 3.0 Å. Simultaneously, Gly66 interacts with the N3 of K777 via hydrogen bonding using the oxygen and backbone nitrogen on the opposite side within the active site, thereby contributing to the stabilization of the inhibitor. These interactions were transiently assisted by additional contacts with Gln19 and Trp184, which formed contacts with the sulfone group of the inhibitor (Table 2). These findings support the role played by these amino acids in stabilizing the inhibitor within the enzyme's active site. On the other hand, the distance from the Cys25 sulfur atom (SG) to the His162 imidazole nitrogen atom (ND1) is, on average, close to 3.33 (Table 1), which is around 0.10 Å lower than the average value obtained for the apo enzyme (3.43 Å). The interaction between K777 and Cz in minimum E, characterized by the presence of the Cys25^−^/His162^+^ ion pair, exhibited relatively consistent distances between the residues and the inhibitor. The average distance from the Cys25 sulfur atom (SG) to the His162 imidazole nitrogen atom (ND1) is 3.09 Å (Table 1), which matches the distance found for the apo enzyme (3.08 Å; Table 1). This suggests that the presence of the inhibitor K777 does not introduce a significant alteration in the geometrical arrangement of Cys25 and His162 in both neutral and charge‐separated states.

**TABLE 2:** HB interactions between residues of Cz and inhibitor K777. The values were computed along the 300 ns of MD simulations for the minimums A–E.

Cz‐inhibitor K777									
Minimum	K777	Residues	Distance, Å (mean ± SD)^a^	(%)^b^	Minimum	K777	Residues	Distance, Å (mean ± SD)^a^	(%)^b^
A	‐N4	Asp161‐O	3.19 (0.44)	30.4	B	‐N4	Asp161‐O	3.02 (0.29)	45.6
	‐N3	Gly66‐O	2.97 (0.18)	11.5		‐N3	Gly66‐O	2.96 (0.18)	10.7
	‐O4	Gln19‐NE2	4.02 (1.38)	6.6		‐O4	Gln19‐NE2	4.76 (1.06)	3.7
	‐O4	Trp184‐NE1	3.44 (0.55)	4.3		‐O4	Trp184_NE1	3.38 (0.55)	1.8
	‐O3	Gln19‐NE2	3.45 (0.54)	3.0		‐O3	Gln19‐NE2	3.15 (0.14)	4.3
	‐O3	Trp184‐NE1	4.19 (0.87)	1.5		‐O3	Cys25‐N	5.12 (0.83)	0.1
	‐O2	Gly66‐N	3.07 (0.23)	2.1		‐O2	Gly66‐N	3.04 (0.19)	7.5
C	‐N4	Asp161‐O	2.99 (0.27)	41.5	D	‐N4	Asp161‐O	3.02 (0.30)	53.0
	‐N3	Gly66‐O	2.97 (0.25)	11.3		‐N3	Gly66‐O	2.97 (0.19)	10.3
	‐O4	Gln19‐NE2	4.92 (1.07)	3.5		‐O4	Gln19‐NE2	5.13 (0.99)	2.8
	‐O4	Trp184‐NE1	4.11 (0.72)	1.1		‐O4	Trp184‐NE1	4.49 (0.86)	0.52
	‐O3	Gln19‐NE2	3.17 (0.49)	2.4		‐O3	Gln19‐NE2	3.18 (0.59)	9.2
	‐O3	Trp184‐NE1	3.79 (0.83)	1.8		‐O3	Trp184‐NE1	3.79 (0.78)	1.2
	‐O2	Gly66‐N	3.04 (0.20	4.7		‐O2	Gly66‐N	3.07 (0.22)	5.6
E	‐N4	Asp161‐O	2.89 (0.16)	20.3					
	‐N3	Gly66‐O	2.94 (0.16)	9.7					
	‐O4	Gln19‐NE2	4.92 (1.07)	3.51					
	‐O4	Trp184‐NE1	4.11 (0.72)	1.05					
	‐O3	Trp184‐NE1	4.13 (0.76)	1.3					
	‐O3	Gln19‐NE2	5.58 (0.80)	0.2					
	‐O2	Gly66‐N	3.17 (0.22)	3.3					

^a^ Values are means ± SD over *n* = 3 replicas, each 300 ns.

^b^ Occupancy (%) of interaction along the MD simulation.

Regarding the Cz complex with the substrate (Ac‐Ala217‐Ala218‐Ala219‐Gly220‐Ala221‐OCH_3_), its backbone engaged with residues Gln19, Gly66, Ser64, Asp161, and Trp184 throughout the MD simulation (Table 3). Among these interactions, Asp161 consistently exhibited the most frequent interaction with the substrate. The distance from the Cys25 sulfur atom (SG) to the His162 imidazole nitrogen atom (ND1) is, on average, close to 3.38 (Table 1), which is slightly shorter than the value obtained for the apo enzyme. In the substrate‐complex minimum E, variations were observed in the substrate‐Cz interactions, with average HB distances being more pronounced. Notably, interactions involving the substrate complexed with Cz and the ion pair showed certain instabilities in hydrogen bonding, as evidenced by average distances of 3.5 Å and standard deviations exceeding 1.0 Å. On the other hand, a slight decrease in the distance from the Cys25 sulfur atom (SG) to the His162 imidazole nitrogen atom (ND1) (3.02 Å; Table 1), was found compared to the apo enzyme.

**TABLE 3:** HB interactions between residues of Cz and substrate (Ac‐Ala‐Ala‐Ala‐Gly‐Ala‐OCH_3_). The values were computed along the 300 ns of MS simulations for the minimums A–E.

Cz‐substrate									
Minimum	Subst	Residues	Distance, Å (mean ± SD)^a^	(%)^b^	Minimum	Subst	Residues	Distance, Å (mean ± SD)^a^	(%)^b^
A	Ala219‐N	Asp161‐O	2.89 (0.13)	68.56	B	Ala219‐N	Asp161‐O	2.88 (0.13)	62.39
	Ala218‐N	Gly66‐O	2.99 (0.19)	32.26		Ala218‐N	Gly66‐O	2.97 (0.18)	33.07
	Gly220‐O	Gln19‐NE2	2.89 (0.14)	23.30		Gly220‐O	Gln19‐NE2	2.89 (0.16)	20.20
	Gly220‐N	Asp161‐O	2.96 (0.17)	20.74		Gly220‐N	Asp161‐O	2.95 (0.16)	21.19
	Ala217‐N	Gly66‐O	3.86 (0.96)	8.95		Ala217‐N	Gly66‐O	3.86 (0.95)	9.97
	Ala218‐O	Gly66‐N	3.05 (0.19)	7.61		Ala218‐O	Gly66‐N	3.05 (0.19)	6.85
	Gly220‐O	Trp184‐NE1	3.78 (0.62)	1.42		Gly220‐O	Trp184‐NE1	3.88 (0.71)	2.01
C	Ala219‐N	Asp161‐O	2.89 (0.13)	41.50	D	Ala219‐N	Asp161‐O	2.88 (0.12)	39.98
	Ala218‐N	Gly66‐O	3.01 (0.19)	47.19		Ala218‐N	Gly66‐O	2.97 (0.19)	47.05
	Gly220‐O	Gln19‐NE2	2.89 (0.14)	5.97		Gly220‐O	Gln19‐NE2	2.88 (0.13)	3.43
	Gly220‐N	Asp161‐O	2.96 (0.17)	18.46		Gly220‐N	Asp161‐O	2.95 (0.16)	18.58
	Ala217‐N	Gly66‐O	3.78 (0.93)	15.14		Ala217‐N	Gly66‐O	4.05 (0.97)	12.11
	Ala218‐O	Gly66‐N	3.05 (0.18)	0.89		Ala218‐O	Gly66‐N	3.05 (0.19)	0.80
	Gly220‐O	Trp184‐NE1	3.82 (0.63)	1.48		Gly220‐O	Trp184‐NE1	3.85 (0.67)	2.30
E	Ala219‐N	Asp161‐O	4.32 (2.29)	30.85					
	Ala218‐N	Gly66‐O	3.78 (1.06)	21.30					
	Gly220‐N	Asp161‐O	5.75 (2.78)	3.01					
	Gly220‐N	Ser64‐O	5.82 (2.59)	5.36					
	Ala217‐N	Gly66‐O	3.27 (0.63)	30.05					
	Ala218‐N	Asp161‐O	4.77 (1.02)	4.22					
	Ala219‐N	Ser64‐O	5.49 (1.68)	1.95					

^a^ Values are means ± SD over *n* = 3 replicas, each 300 ns.

^b^ Occupancy (%) of interaction along the MD simulation.

The results for minimum E (system with an initial point containing an ion pair) demonstrate that the substrate does not exhibit stability throughout the simulation. The RMSD analysis (Figure 6) reveals that at the end of the simulation there is an increase in the RMSD values for the complex, which is caused by the substrate's structural changes during the simulation, leading it to exit the active site. While the arrangement of Cys25 and His162 remains stable throughout the trajectory (Figure S9), the distance from the catalytic dyad (Cys25 and His162) to the center of mass of the substrate increases to above 12.0 Å at the end of the simulation (Figure S9), suggesting that the ion pair system does not offer stability for the substrate's binding. The RMSD of the substrate (Figure S10) indicates the occurrence of several rearrangements of the ligand along the simulation, with a partial loss of interactions with residues at the active site at around 75 ns. These findings would also support the notion that the substrate needs to bind to the active site with the Cys and His residues in their neutral forms. In fact, the interactions between the substrate in the Ala217 and Ala218 regions with the protein's Gly66 occur at distances of 3.3 Å (±0.63 Å) and 3.8 Å (±1.06 Å), respectively. Over the 300 ns simulation, the substrate adopts configurations that lead to its gradual exit from the active site through the opposite end, near the Gly220 and Ala221 regions, while the area around Ala217 and Ala218 remains in contact with the protein for a longer period (Figure S10).

### 3.1 Constant pH simulations

**FIGURE 7:**
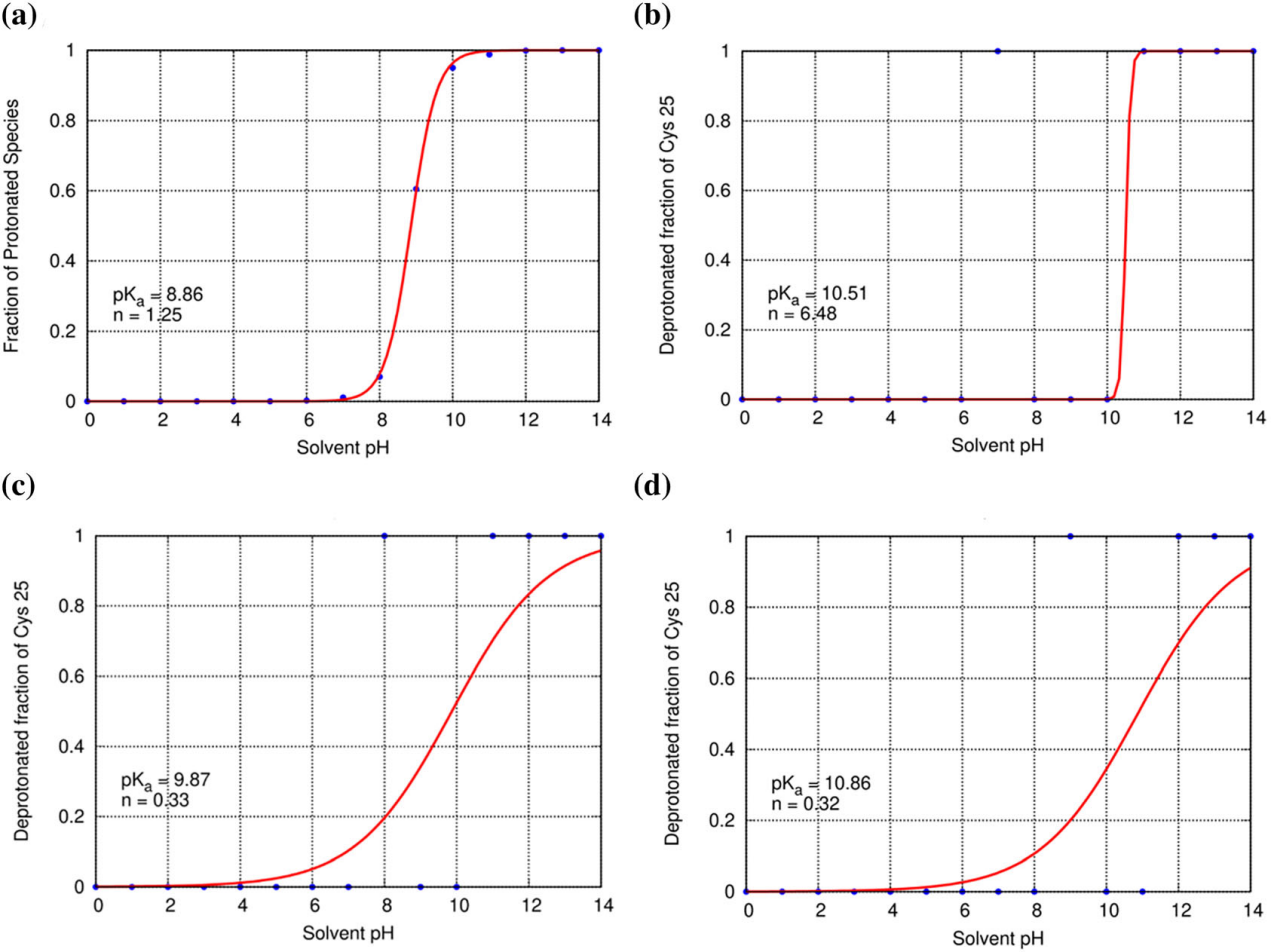
Titration curves for (a) cysteine residue in water, (b) Cys25 in ligand‐free Cz, (c) Cys25 in K777‐Cz complex, and (d) Cys25 in substrate‐Cz complex. The plots shown in panels b–d were selected as representative examples from the Supporting Information (Figures S11–S20).

CpHMD simulations were carried out for the following systems: Cys residue in solution, ligand‐free Cz, Cz‐K777, and Cz‐substrates complexes. The simulations involving the enzymatic environment were performed taking into consideration two possible protonation states for His162: neutral and positively charged (Figures S11 and S20 show the titration curves corresponding to each system). The titration curves obtained from the CpHMD simulations of Cys25 in water and protein environments are depicted in Figure 7. The calculated p*K*a value of the cysteine residue in reference water solution was 8.9 (Figure 7a), which agrees with the experimental estimation of p*K*a for the Cys side chain (8.6) (Roos et al., 2012). The calculated average p*K*a values for Cys25 in the ligand‐free Cz correspond to 10.2 (see Table S2), which is in agreement with the experimental value of p*K*a of 9.8 (Zhai & Meek, 2018).

Panels B, C, and D show representative titration curves for Cys25 in the active site of the ligand‐free Cz enzyme, the Cz‐K777 inhibitor complex, and the Cz‐substrate complex, respectively (Figure 7). Average p*K*a values are found in Table S2, and the plots shown in panels B, C, and D (Figure 7) were selected as representative examples from the Supporting Information (Figures S8–S27). The calculated p*K*a value for Cys25 in the ligand‐free Cz is 10.5 for one replica (Figure 7b), and the average p*K*a amounts to 10.2 (see Table S2), which is in agreement with the experimental p*K*a of 9.8 (Zhai & Meek, 2018). Upon binding of the covalent inhibitor K777 and substrate, the p*K*a of Cys25 shifts slightly to 9.9 and 10.9, respectively (Figure 7c,d).

To assess the pKa shift of Cys25 in the Cz active site upon ligand binding, CpHMD calculations were conducted for both Cz‐K777 and Cz‐substrate complexes, accounting for two potential protonation states of His162: neutral and positively charged. In the first case, with neutral His162, the analyses indicated no titration of Cys25 across the entire pH range from 0 to 14 for both Cz‐K777 and Cz‐substrate complexes (see Figures S11–S20). When His162 is protonated (Figure 7; see also Figures S8–S27), the average p*K*a value of Cys25 is 10.1 and 11.9 for the Cz‐K777 and Cz‐substrate complexes, respectively (see Table S2). These results support the existence of a possible neutral Cys25‐His162 dyad in the ligand‐free Cz, Cz‐K777 and Cz‐substrates complexes.

Previous studies indicated that predicting p*K*a values for Cys residues is exceedingly challenging. Hofer and colleagues (Hofer et al., 2020) employed CpHMD with implicit solvent as a tool for predicting p*K*a values of the apoenzymes for aspartic, cysteine, and serine proteases. Furthermore, Awoonor‐Williams and coworkers also employed CpHMD in both implicit and explicit solvent to predict the p*K*a of cysteines in free‐ligand enzymes, including the catalytic ones. Nevertheless, their assessment identified discrepancies in defining the behavior of cysteine titration during constant pH analysis. One of the reasons why accurate pKa values could not be estimated is the problematic treatment of the sulfur atom and its titration within the context of partial charges. This issue arises in both the ff14SB and Amber99SB force fields, which were used in the respective studies. Describing sulfur or titration is not an issue when considering an isolated cysteine. Herein, we were able to capture the correct p*K*a for the free enzyme, Cz‐K777 and Cz‐substrate, when His162 is positively charged. The challenge to explore the p*K*a shift of Cys25 emerges when cysteine is located in a dense, tightly packed environment, such as the ligand‐bound state in the holo enzyme.

Curiously, Shen and coworkers were able to reproduce the experimental p*K*a values of cysteine residues using implicit solvent based on the GBNeck2 and with the ff14SB, which can improve the computational cost associated. Nevertheless, the presence of a ligand can significantly alter the electrostatic and steric environment within the active site, and CpHMD's simplified models for protonation and deprotonation can lead to significant deviations in p*K*a predictions. The substantial perturbations of the sampled conformations during CpHMD analysis are not adequately captured. In addition, this could also be attributed to the pronounced polarizability of sulfur, an effect neglected in tested CpHMD approaches due to the absence of polarizable force fields. At this point, Hofer and colleagues (Hofer et al., 2020) proposed that a more precise definition of sulfur's electrostatic interactions or the incorporation of polarizable force fields would significantly enhance p*K*a prediction.

### 3.2 Free energies for estimating p*K*a shifts

To address the problem of inadequate description of the thiol and thiolate groups in the Amber99SB force field, researchers in (Awoonor‐Williams et al., 2023) employed TI calculations with refined parameters. However, the outcomes showed only a marginal improvement of 0.5 log units in the Δp*K*a. Here, we have also employed the TI method to estimate the p*K*a shift of Cys when Cz is in complex with substrate and inhibitor, considering His162 in its neutral state.

The pKa shift of Cys25 in the active site of Cz was explored considering the neutral cysteine as the initial state (λ = 0.0) and the negatively charged thiolate anion as the final state (λ = 1.0) (see Figures S8–S27 for the energetic analyses obtained for each simulation window). As seen from the results in Table 4, a free energy difference (∆∆G) of 3.3 kcal/mol and p*K*a shift of 2.4 for Cys25 was obtained for the ligand‐free enzyme, which indicates that the transformation of Cys25‐SH to Cys25‐S^−^ was unfavorable. These results are in agreement with the experimental data, which pointed out that Cys25 is protonated in the ligand‐free enzyme (Zhai & Meek, 2018).

**TABLE 4:** Free energy differences (ΔΔG) and pKa changes for cruzain in its different forms: Free, bound to the inhibitor and substrate. The free energy values and pKa changes are presented as averages computed from replicates (see Supporting Information).

	TI (kcal/mol) (mean ± SD)^a^	ΔΔG (kcal/mol)	△p*K*a
Model (in Water)	−78.26 (0.14)		
Cruzain free form	−74.87 (0.16)	3.3	2.4
Cruzain‐K777	−58.58 (0.15)	19.6	14.2
Cruzain‐substrate	−59.50 (0.14)	18.7	13.6

^a^ Values are means ± SD over *n* = 3 replicas, each 300 ns.

In view of this, we also conducted TI calculations in the presence of the ligand and the substrate in the active site (see Figures S21–S40 for the energetic analyses obtained for each simulation window). Free energy differences of 19.6 and 18.7 kcal/mol were obtained for Cys25 in Cz‐K777 and Cz‐substrate complexes, respectively, which also suggest that Cys25 is in its protonated form in Cz‐ligand complexes. Therefore, the neutral Cys‐SH/His dyad is present in the pre‐reactive complex in Cz. It is important to note that the unassisted nucleophilic attack by neutral thiol on the amide unit involves the highest barrier of 31 kcal/mol (Štrajbl et al., 2001), and as expected the neutral cysteine thiol group are moderately nucleophile in comparison with thiolate form of cysteine. Therefore, the existence of the Cys25^−^/His162^+^ ion pair in Cz may occur prior to the nucleophilic attack of Cys25, as suggested by QM/MM free energy calculations (Silva et al., 2020).

The alchemical calculations suggest that the neutral form is populated in both the free enzyme and the ligand‐bound state, which agrees with the findings reported by Moliner and Swiderek, who demonstrated that the most stable protonation state of the Cys145/His41 dyad in the enzyme‐substrate complex of SARS‐CoV‐2 Mpro corresponds to the neutral species (Świderek & Moliner, 2020). The Cys145^−^/His41^+^ ion pair is located in high‐energy regions of the free energy surface for the intermediate and final steps of the catalytic cycle, indicating that this protonated state is unfavorable in the case of Mpro. They suggest that the absence of a conserved residue (Asp/Glu) interacting with the δ‐nitrogen of His41 is normally responsible for modulating its p*K*a (Świderek & Moliner, 2020).

On the other hand, the existence of the ion pair was experimentally proven by different studies (Creighton et al., 1980; Keillor & Brown, 1992; Lewis et al., 1981) and also explained by computational modeling. For instance, Gul et al. (2008), utilizing normal mode analysis, demonstrated that in papain the motion of the cleft around Trp177 exhibits asymmetric open‐close dynamics (Gul et al., 2008). This motion allows Trp177 to glide over His159, resulting in the exposure of the imidazolium side chain to the solvent. This interaction highlights the potential role of Trp residues in facilitating the generation of reactive intermediates, CysS^−^/HisH^+^ ion pair.

The formation of the ion pair in either the free or ligand‐bound enzyme is highly system‐dependent. As previously noted, in cysteine proteases, the ion pair may form either before or after ligand binding, depending on the specific enzyme species. This suggests that there is no general mechanism for covalent inhibition or catalysis in cysteine proteases. In some cases, a concerted proton transfer from cysteine to histidine, or to a water molecule, may occur simultaneously with nucleophilic attack on the electrophilic warhead of the ligand. In other cases, proton transfer from cysteine to histidine precedes ligand binding.

Cz exhibits a population of the neutral form in both the free enzyme and ligand‐bound states (see above). One factor that may trigger the formation of the CysS^−^/HisH^+^ ion pair may be Trp184 (Figure 5), in combination with the local electrostatic environment. The presence of Trp184 could induce conformational changes that influence the electric field of the active site, thereby affecting the p*K*a of the Cys25 and His162 residues and lowering the activation barrier for proton transfer. Additionally, the electronic distribution within an aromatic ring often favors the positioning of a neighboring positively charged side chain directly above the ring, as noted in protein structures (Fernández‐Recio et al., 1997). In this sense, protonation of the His residue strengthens its interaction with a neighboring tryptophan. Interactions between aromatic and positively charged residues are commonly observed in proteins and can play a crucial role in stabilizing intermediate states during biochemical reactions (Fernández‐Recio et al., 1997).

The formation of the ion pair is closely tied to the electric field within the enzyme's active site, which influences the p*K*a values of Cys and His residues and affects the activation barrier for proton transfer. Recent studies have highlighted the importance of this interplay in controlling the catalytic mechanism (Zheng et al., 2023), stating that enhancing the active site electric field aids the enzyme in accelerating the catalysis by lowering the activation barrier. In this way, when a ligand binds to the Cz active site, it impacts the arrangement of the microenvironment of the active site, inducing a change in the electric field and lowering the p*K*a of Cys25; hence, the proton is transferred to His162 through a lower activation energy barrier, and the ion pair is formed in a pre‐reactive state.

Overall, the protein environment significantly influences the p*K*a of certain residues, as seen in the active site of enzymes like papain. Specifically, in the case of Cz, the imidazole group of His162 polarizes the SH group of Cys25, facilitating its deprotonation and the formation of a highly nucleophilic CysS^−^/HisH^+^ ion pair. However, it is important to note that the neutral state, where both Cys and His residues are uncharged, typically represents the ground state of the Cz enzyme. Under certain conditions, this neutral state transitions to the ion pair (CysS^−^/HisH^+^), which acts as a crucial intermediate along the reaction pathway. This observation is in line with what Zhai and Meek have observed for Cz through solvent kinetic isotope effects, demonstrating that both residues remain neutral in free Cz (Zhai & Meek, 2018).

## 4 CONCLUSION

In this paper, we have explored the possible protonation states of the catalytic residue Cys25 in Cz. The MD simulations show that Cys25 forms a weak hydrogen bond with the His162 residue, which may support the idea of a neutral Cys25/His162 dyad in the free form of Cz. The calculated p*K*a value for Cys25 in ligand‐free Cz is 10.2, which agrees with the experimental p*K*a value of 9.8. The free energy calculations indicate that the catalytic Cys25/His162 dyad is neutral in the free form, but they also indicate that the catalytic dyad is neutral in complex with K777 and with the substrate. In this context, the MD simulations suggest that the neutral Cys‐SH/His dyad is present in the pre‐reactive complex in Cz. Therefore, the potential occurrence of a CysS^−^/HisH^+^ ion pair may happen prior to the nucleophilic attack of Cys25, which could be triggered by changes in the local electric field. Altogether, these results are useful for drug design projects, where the details of the protonation state of catalytic residues are critical for docking and molecular mechanics‐based studies and for understanding the catalytic mechanism and inhibition.

## AUTHOR CONTRIBUTIONS

**Clauber H. S. da Costa:** Investigation; methodology; visualization; validation; writing – original draft. **Vinícius Bonatto:** Methodology; data curation; writing – original draft. **Hemillin Brenda Teixeira Santos:** Formal analysis; data curation; methodology; visualization; writing – review and editing. **Carlos Gabriel da Silva de Souza:** Validation; visualization; methodology. **Carlos A. Montanari:** Validation; formal analysis; supervision; project administration; funding acquisition. **Munir S. Skaf:** Validation; formal analysis; supervision; project administration; funding acquisition. **F. Javier Luque:** Writing – review and editing; supervision; formal analysis; conceptualization. **Jerônimo Lameira:** Conceptualization; methodology; writing – review and editing; writing – original draft; project administration; funding acquisition; formal analysis.

## Supporting information


**Data S1.** Supporting Information.

## Data Availability

The data that support the findings of this study are available from the corresponding author upon reasonable request.
