# Supplementary material for: Evaluating the protonation state of the catalytic Cys25 in cruzain cysteine protease: A target for Chagas disease
Source: Protein Sci. 2025 Sep 17;34(10):e70283. doi: 10.1002/pro.70283 (PMC12442452; doi:10.1002/pro.70283)
Supplement: Supplementary file 1 — Data S1. Supporting Information. [file PRO-34-e70283-s001.docx]

**Evaluating the protonation state of the catalytic Cys25 in Cruzain cysteine protease: A target for Chagas disease**

Clauber H. S. da Costa,^a^ Vinícius Bonatto,^b^ Hemillin Brenda Teixeira Santos,^c^ Carlos Gabriel da Silva de Souza,^c^ Carlos A. Montanari,^b^ Munir S. Skaf,^a^ F. Javier Luque,^d^ Jerônimo Lameira^,b,c*^

^a^ Institute of Chemistry and Center for Computing in Engineering & Sciences, University of Campinas – UNICAMP. Campinas, SP 13084-862, Brazil.

^b^Grupo de Química Medicinal do Instituto de Química de São Carlos da Universidade de São Paulo, NEQUIMED/IQSC/USP. 13566-590. São Carlos/SP. Brazil

^c^Laboratory of Computer Modeling of Molecular Biosystems (CompMBio), Federal University of Pará, Belém 66075-110, Brazil.

^d^Department of Nutrition, Food Science and Gastronomy, Faculty of Pharmacy and Food Science-Campus Torribera, Institute of Biomedicina (IBUB) and Institute of Theoretical and Computational Chemistry (IQTCUB), University of Barcelona, Santa Coloma de Gramenet 08921, Spain

*Corresponding Authors: [lameira@ufpa.br](mailto:lameira@ufpa.br) (JL), hemillin.santos@ics.ufpa.br (HBTS)

**Supporting Information**

4
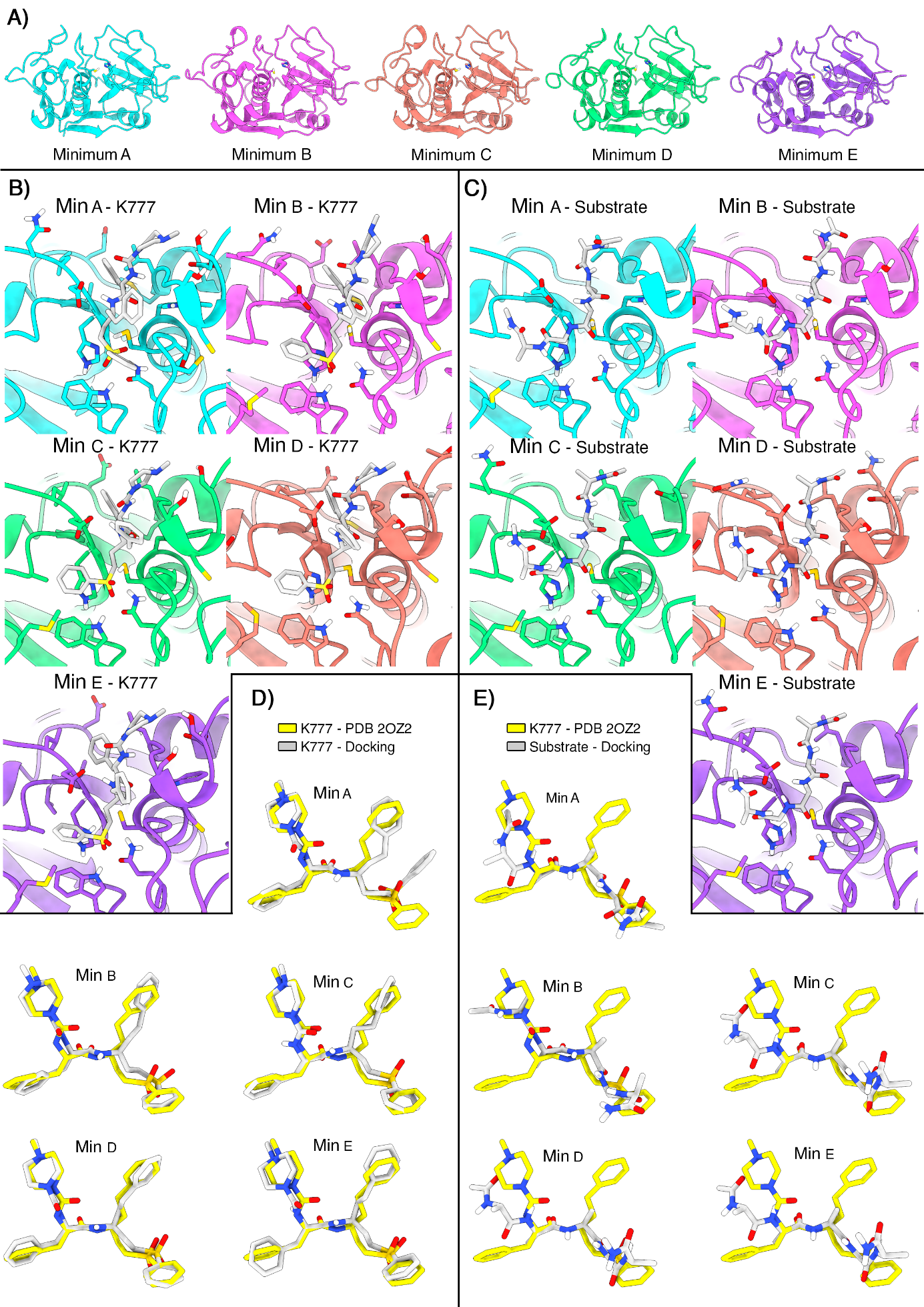


**Figure S1**. Molecular docking results of the **K777** inhibitor and substrate for the Cz protein structures defined by the minima found in the PCA/FEL analysis. A) Representation of the protein backbone for the five models extracted from the two-dimensional free energy landscape plots (Figure 5). B,C) Best docked poses obtained for **K777** and substrate in the five protein models. D,E) Comparison of the docked poses of **K777** and substrate with the X-ray crystallographic structure of **K777** bound to CZ (PDB ID 2OZ2).

**Table S1**. Molecular docking results for the inhibitor **K777** and substrate (Ac-Ala-Ala-Ala-Gly-Ala-OCH_3_) in the receptors found at the minima of the simulations, using the Molegro Virtual Docker program.

| **Inhibitor K777** | | | | | |
| --- | --- | --- | --- | --- | --- |
|  | **Start point 1** | | **Start point 2** | | **Start point 3** |
|  | **Min A** | **Min B** | **Min C** | **Min D** | **Min E** |
| **Moldock score** | -88.0 | -199.3 | -119.5 | -80.7 | -151.8 |
| **E - Hbond** | 0.7 | -7.3 | -9.1 | -10.9 | -9.2 |
| **Substrate (Ac-Ala-Ala-Ala-Gly-Ala-OCH_3_)** | | | | | |
| **Moldock score** | -80.7 | -106.5 | -93.7 | -64.5 | -91.8 |
| **E - Hbond** | -6.6 | -9.1 | -6.3 | -4.6 | -4.8 |


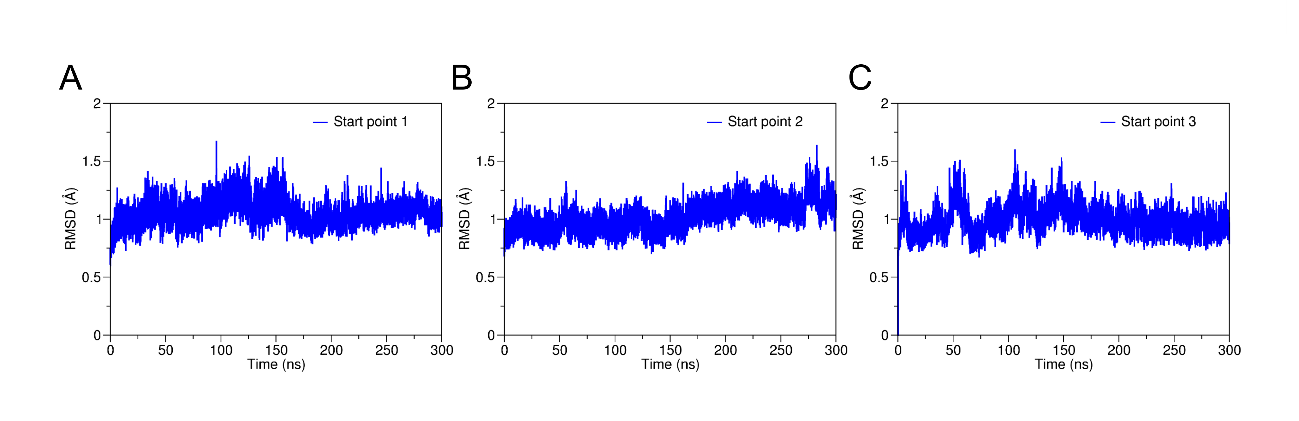


**Figure S2.** Time evolution of the RMSD profile (Å) determined for the protein backbone for the three simulations of free protein structure (cruzain free form) starting from Start points 1-3.


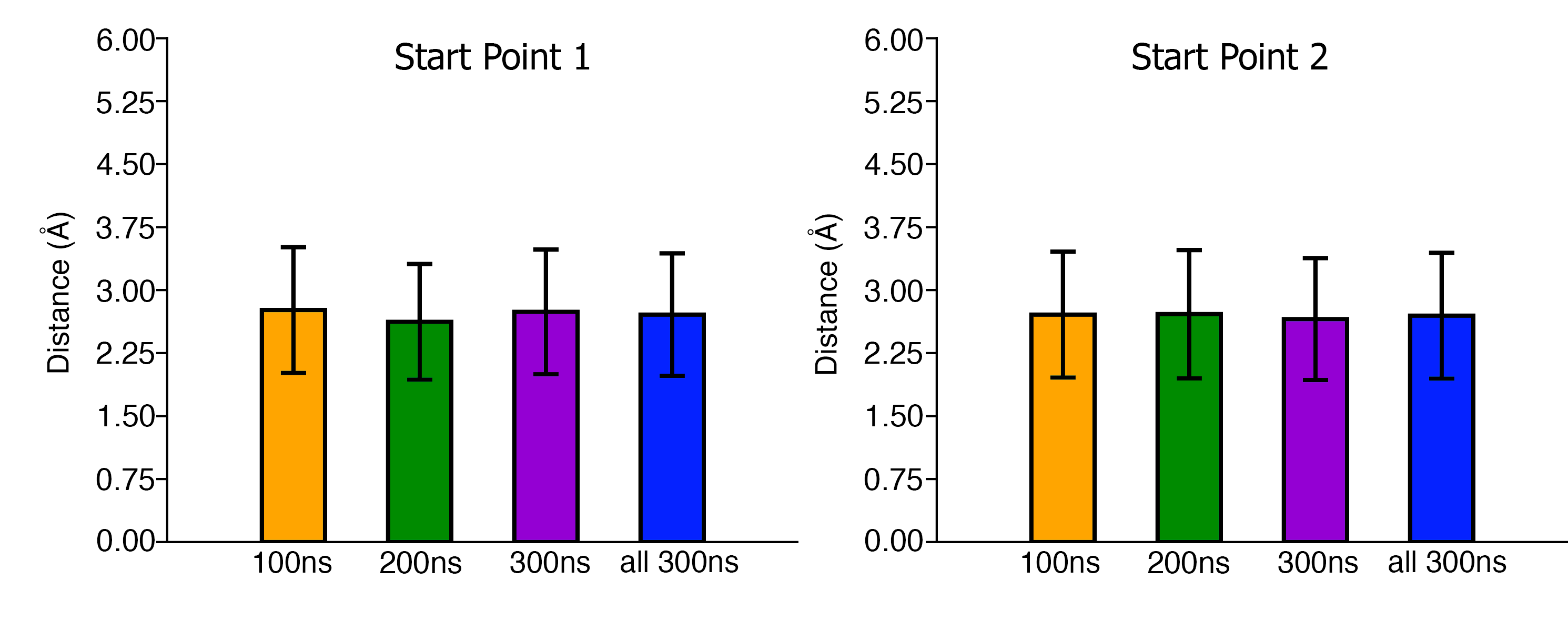


**Figure S3**. Atomic distance (Å) between the thiol hydrogen of Cys25 to the Nδ of His162 for Start point 1 and 2 (neutral states) of the cruzain free form.


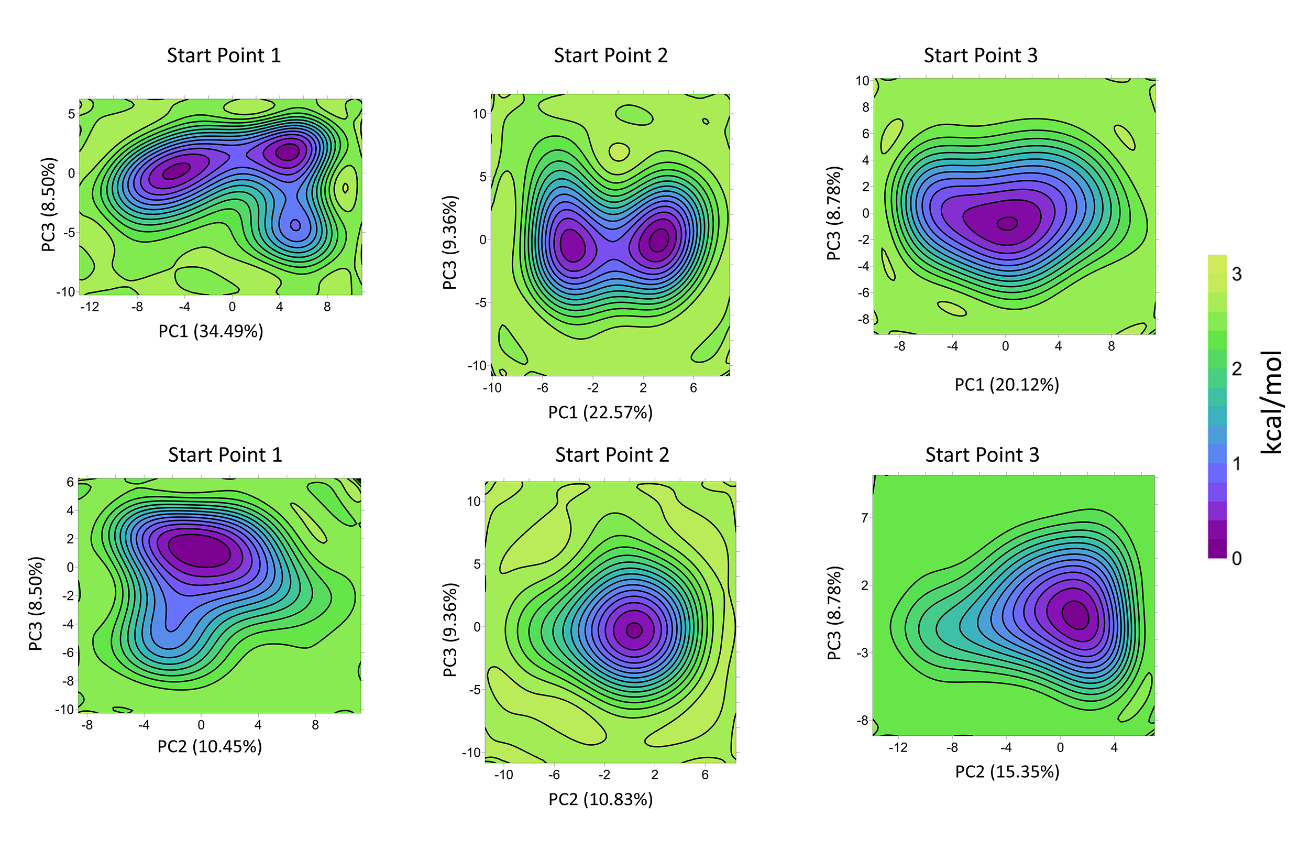


**Figure S4**. Representation of the FEL for (top) PC1 vs PC3 and (bottom) PC2 vs PC3 for start points 1, 2, and 3.

**
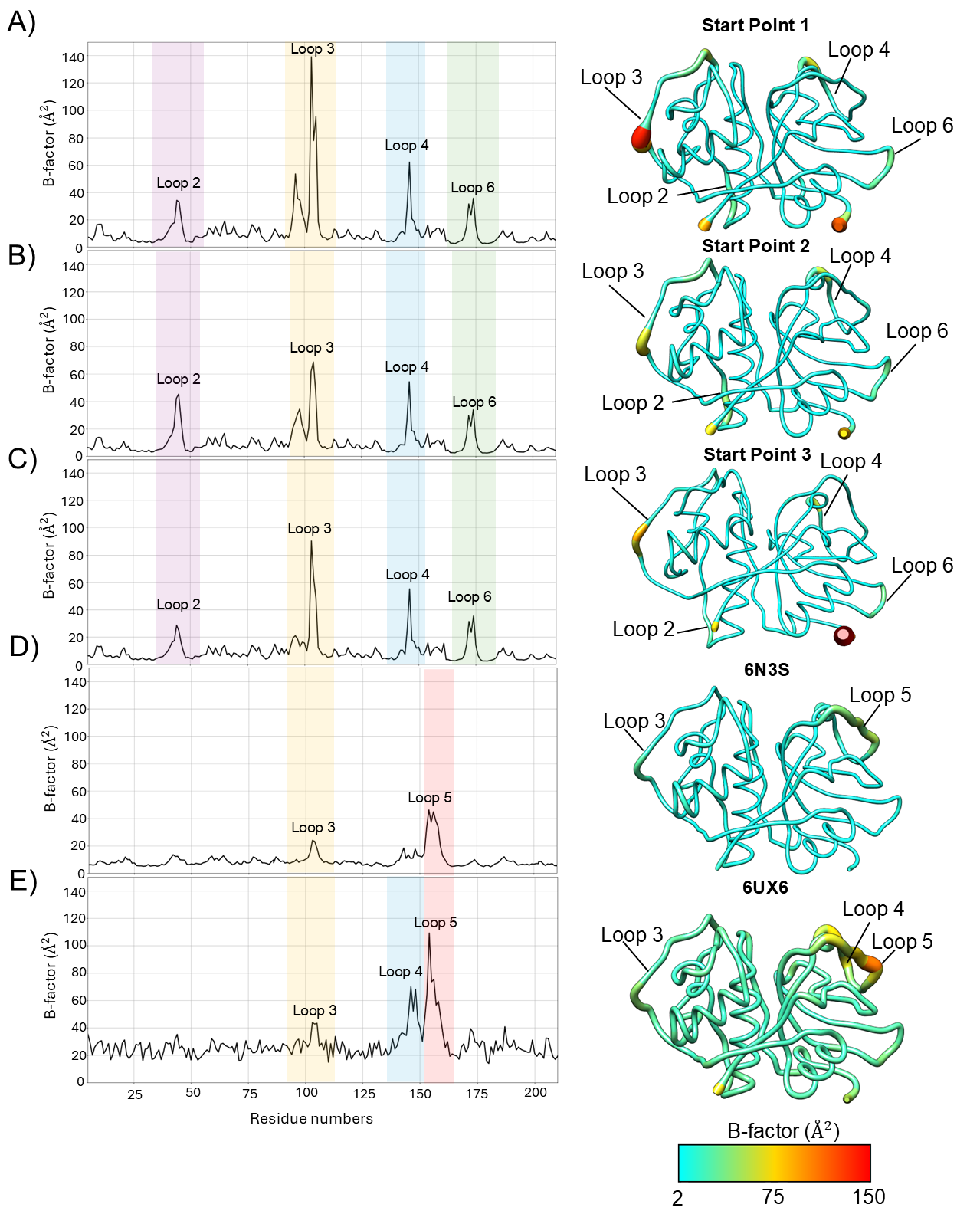
**

**Figure S5**. B-factor analysis of cruzain free form. A, B and C) MD-derived B-factor profiles for Start Points 1, 2 and 3, highlighting flexible loops (3, 4, 6). Side panels show corresponding 3D structures colored by flexibility. D and E) Crystallographic B-factors from PDB entries 6N3S and 6UX6, with flexibility concentrated in loops 3, 4, and 5.


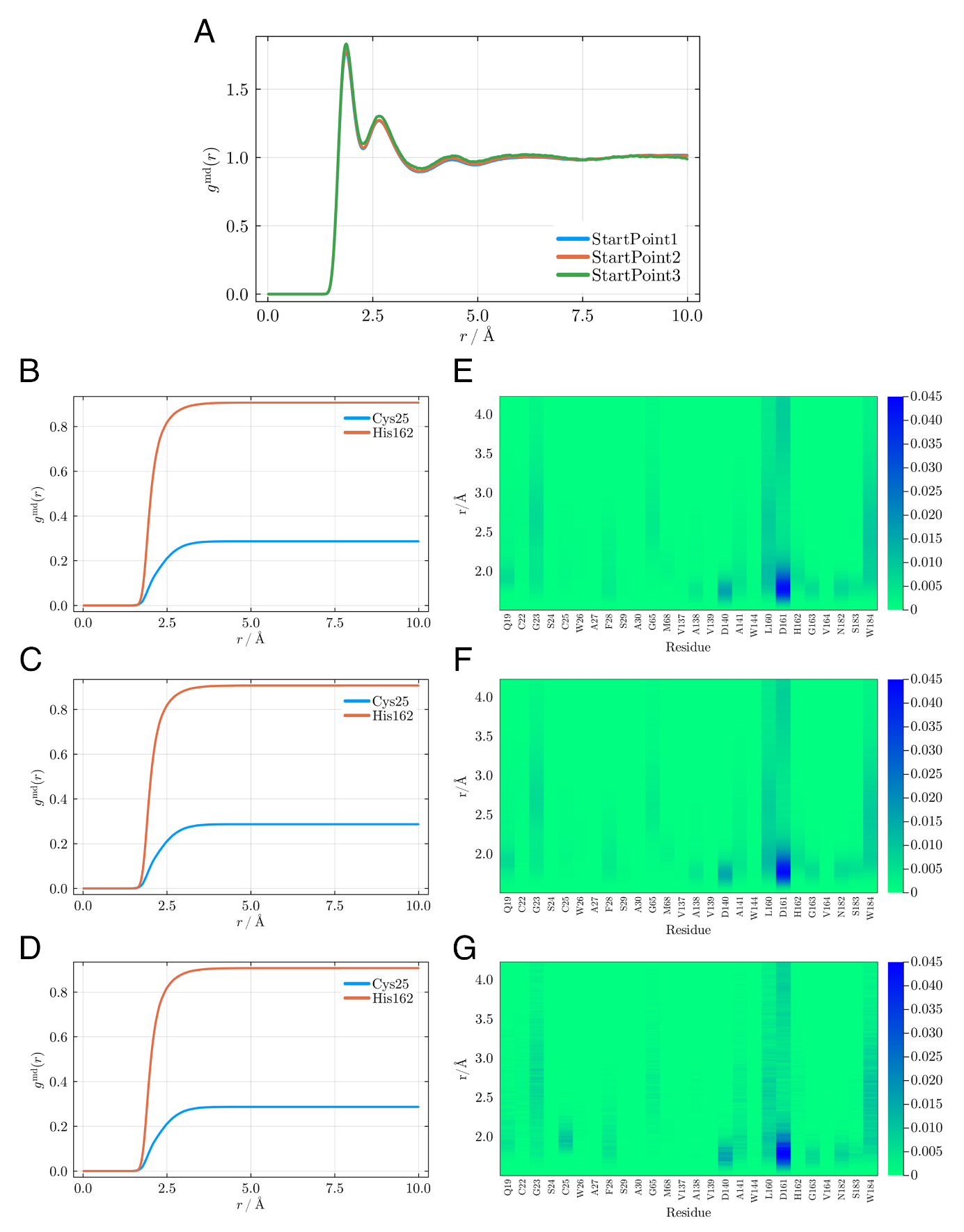


**Figure S6**. MDDF analysis of cruzain free form systems (Start Points 1, 2 and 3). A) Global protein–water MDDFs show conserved hydration shells. B, C and D) Residue-specific MDDFs for Cys25 and His162 indicate higher water coordination for His162. E, F and G) Per-residue decomposition highlights structured water interactions around key active site residues, including Asp140, Asp161, and His162.


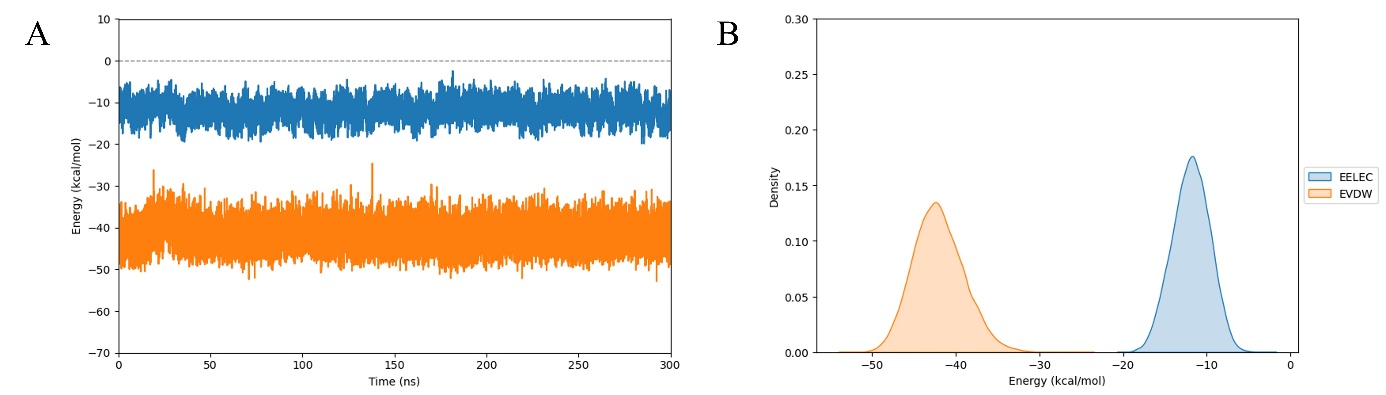
**Figure S7**. Electrostatic and van der Waals energies along the MD simulations for the Cz–substrate system (minimum E)


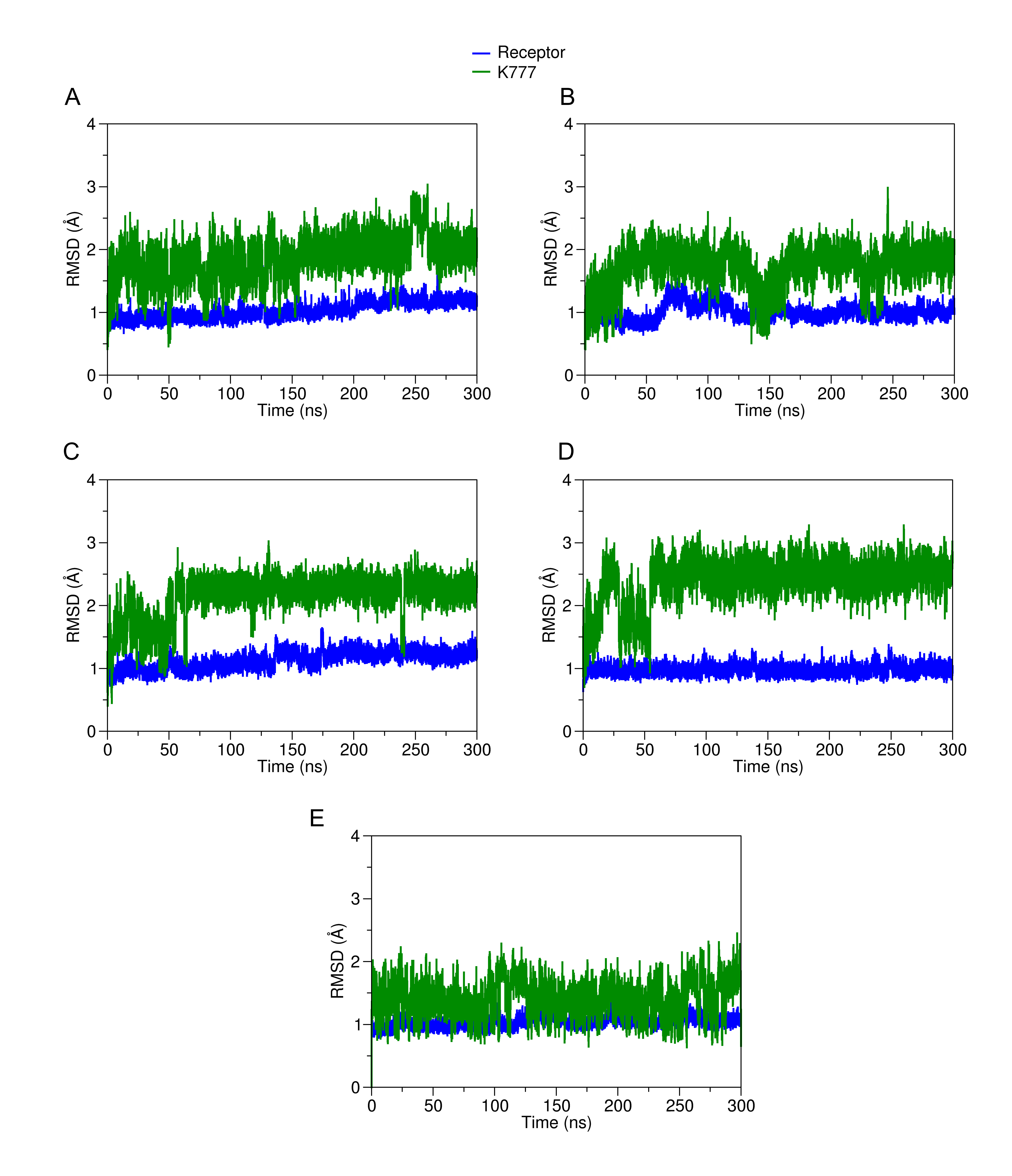


**Figure S8.** Time evolution of the RMSD (Å) of the **K777** inhibitor (green) and the backbone of Cz (blue) for the Cz-**K777** complexes built up from energy minima A-E.


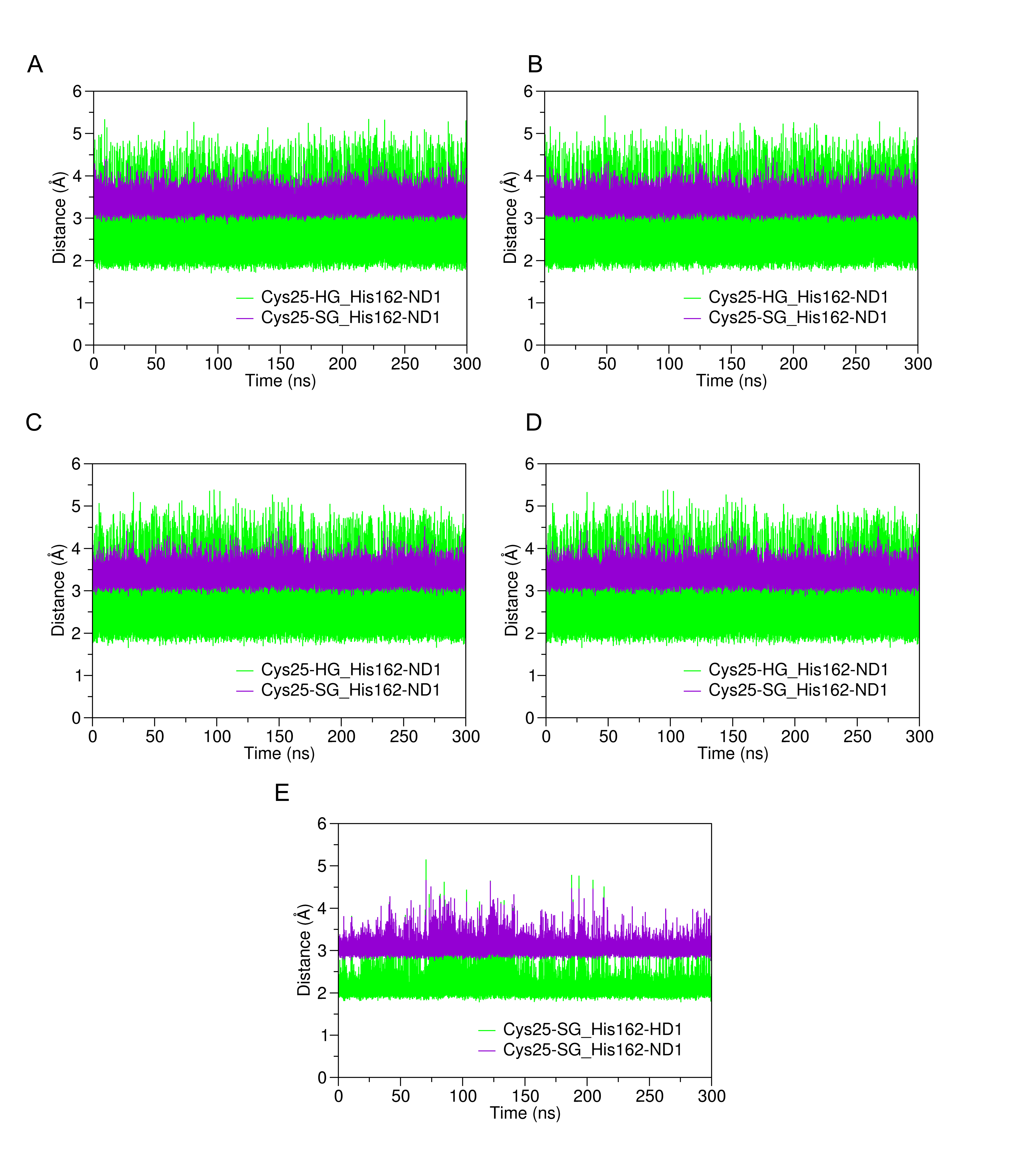


**Figure S9**. Atomic distance (Å) between the hydrogen and sulfur atom of Cys25 to the Nδ of His162 for the systems corresponding to the substrate-bound species in the holo form of the enzyme.


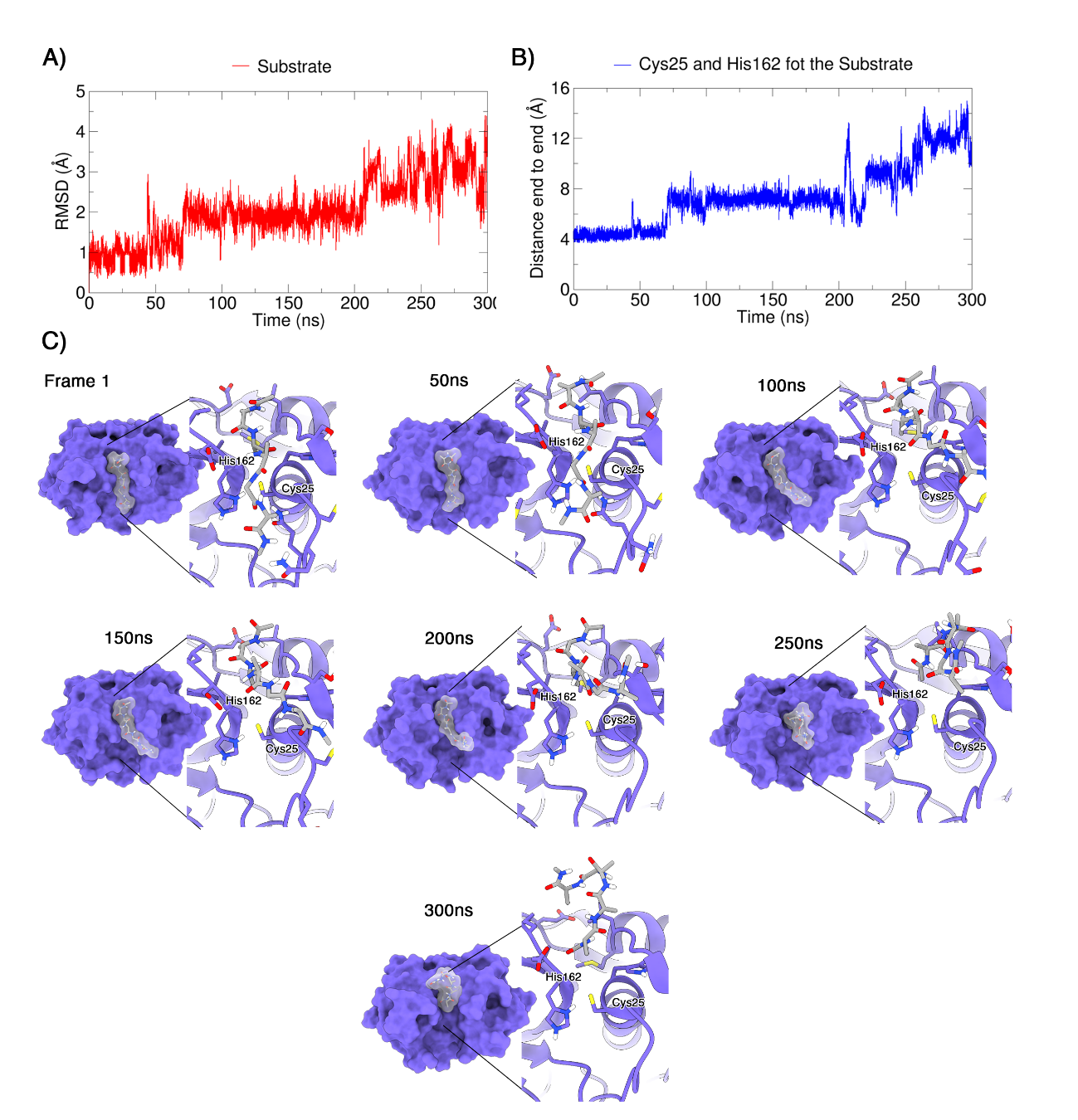


**Figure S10**. A) Time evolution of the RMSD (Å) of the substrate (Ac-Ala-Ala-Ala-Gly-Ala-OCH_3_) throughout the 300 ns simulation in minimum E. B) Distance (Å) from the center of mass between Cys25 and His262 to the substrate. C) Configurations of the substrate in the active site of Cz at selected points during the 300 ns.


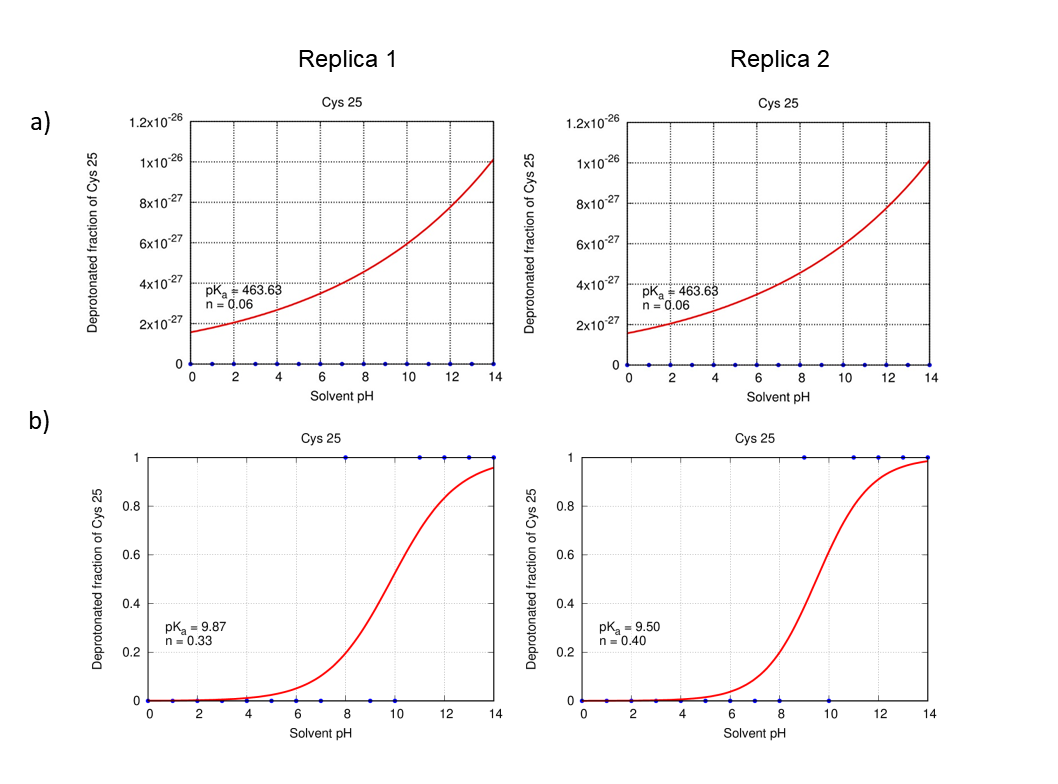


**Figure S11.** Titration curves of Cys25 in the Cz-**K777** complex, minimum A. The pKa of Cys25 was evaluated considering two cases: (a) neutral His162 and (b) positively charged His162.


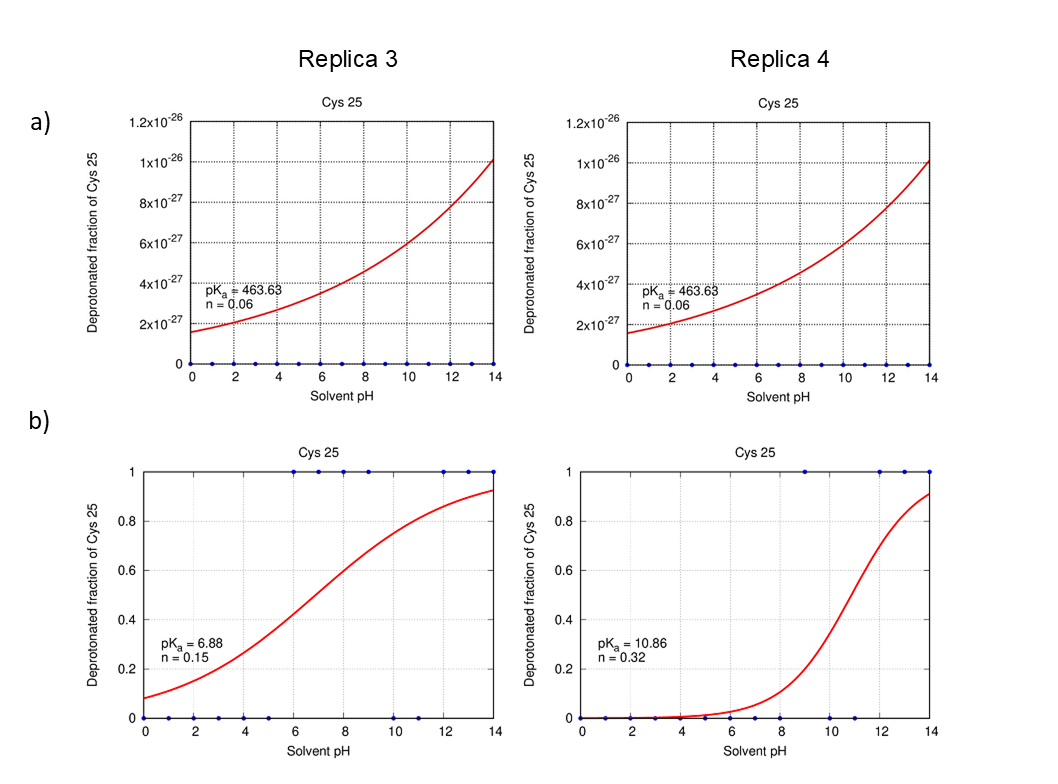


**Figure S12.** Titration curves of Cys25 in the Cz-**K777** complex, minimum B. The pKa of Cys25 was evaluated considering two cases: (a) neutral His162 and (b) positively charged His162.


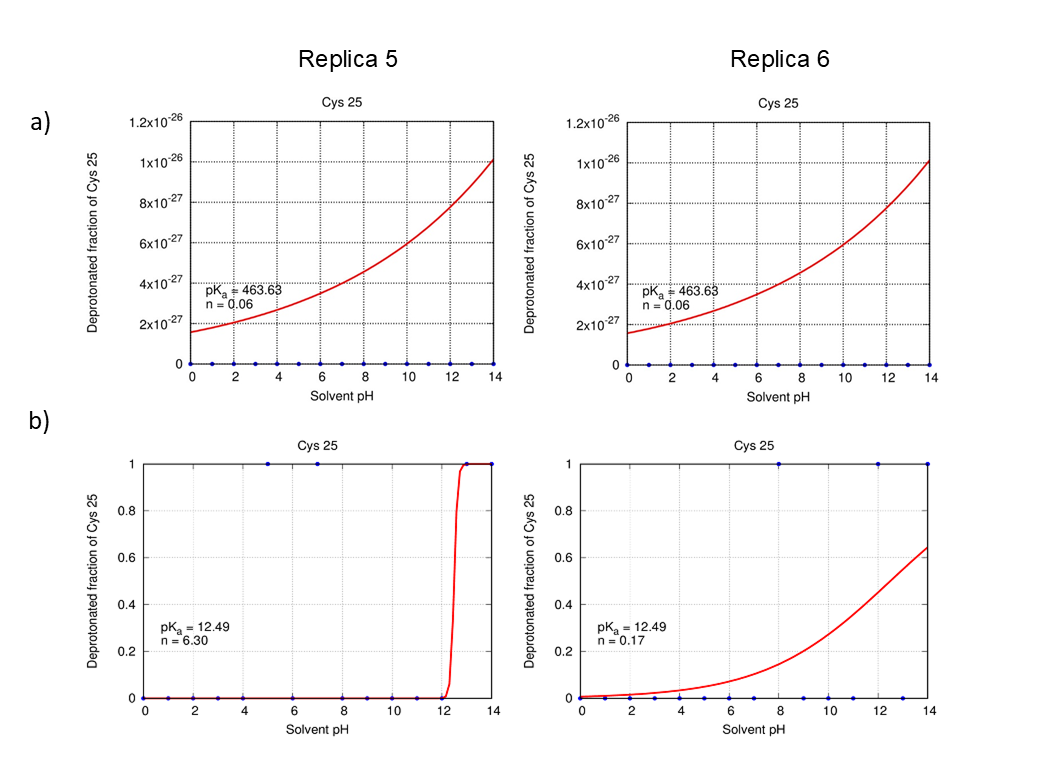


**Figure S13.** Titration curves of Cys25 in the Cz-**K777** complex, minimum C. The pKa of Cys25 was evaluated considering two cases: (a) neutral His162 and (b) positively charged His162.


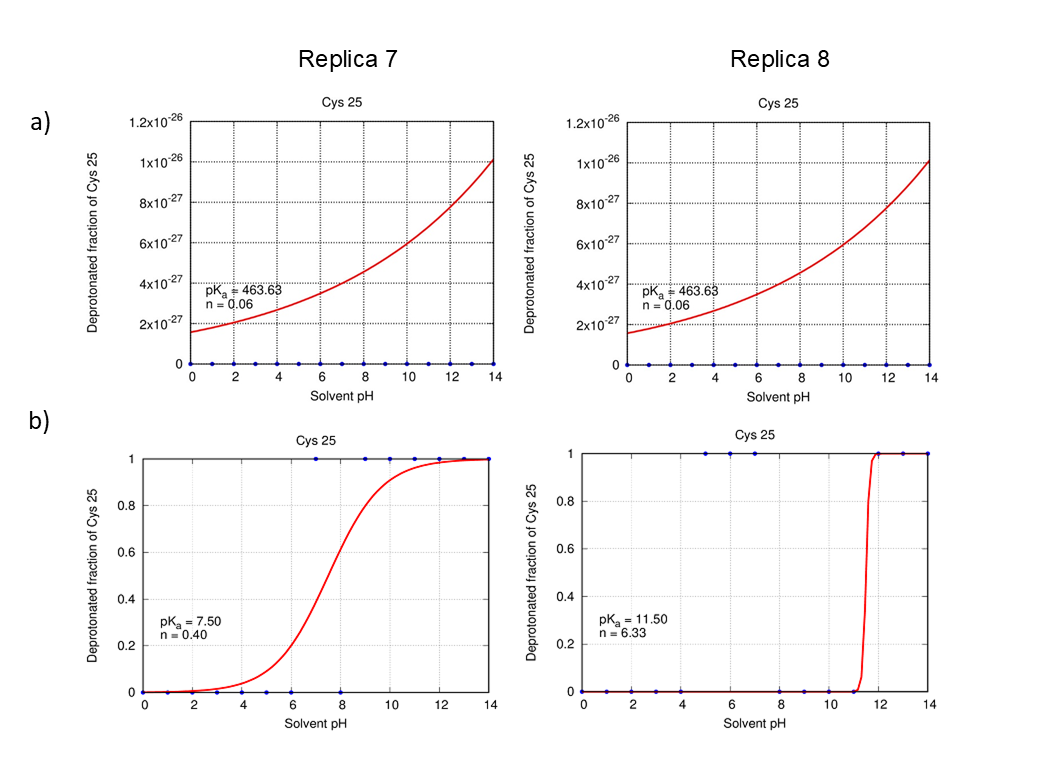


**Figure S14.** Titration curves of Cys25 in the Cz-**K777** complex, minimum D. The pKa of Cys25 was evaluated considering two cases: (a) neutral His162 and (b) positively charged His162.


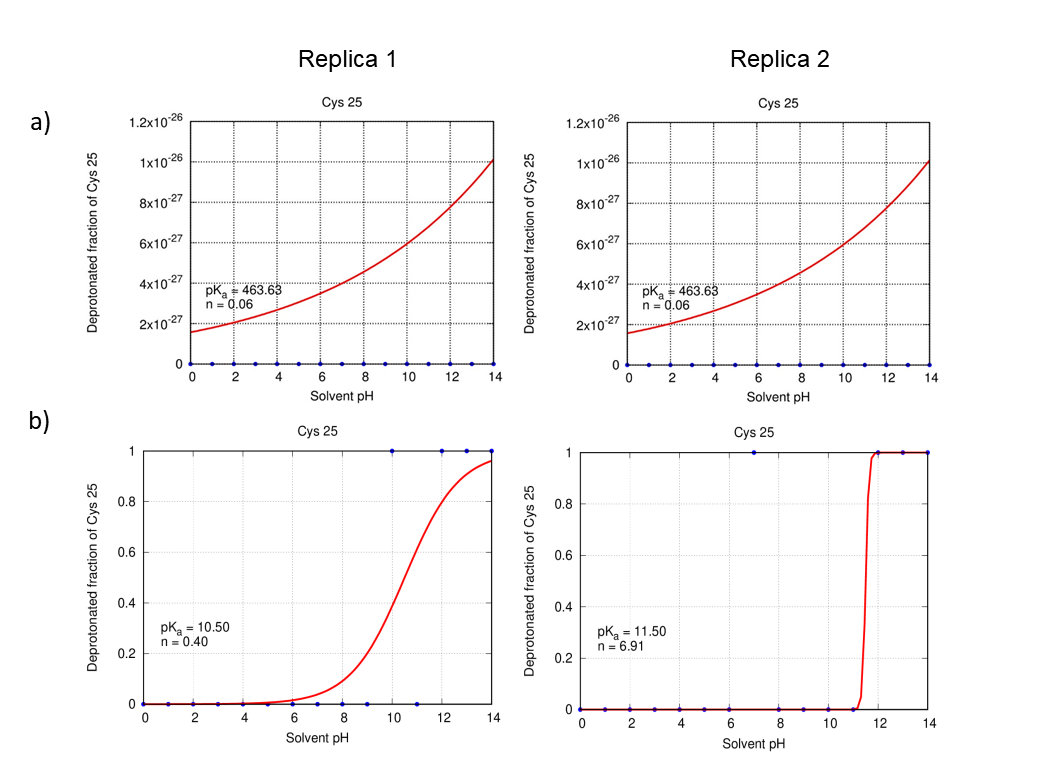


**Figure S15.** Titration curves of Cys25 in the Cz-substrate complex, minimum A. The pKa of Cys25 was evaluated considering two cases: (a) neutral His162 and (b) positively charged His162.


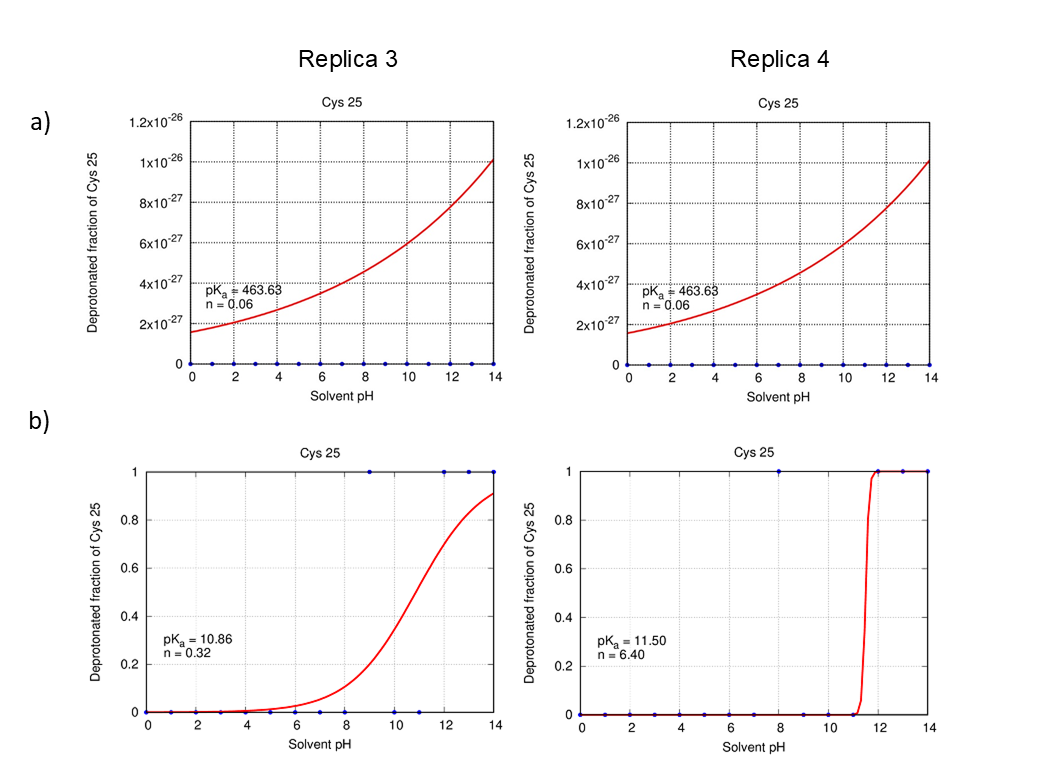


**Figure S16.** Titration curves of Cys25 in the Cz-substrate complex, minimum B. The pKa of Cys25 was evaluated considering two cases: (a) neutral His162 and (b) positively charged His162.


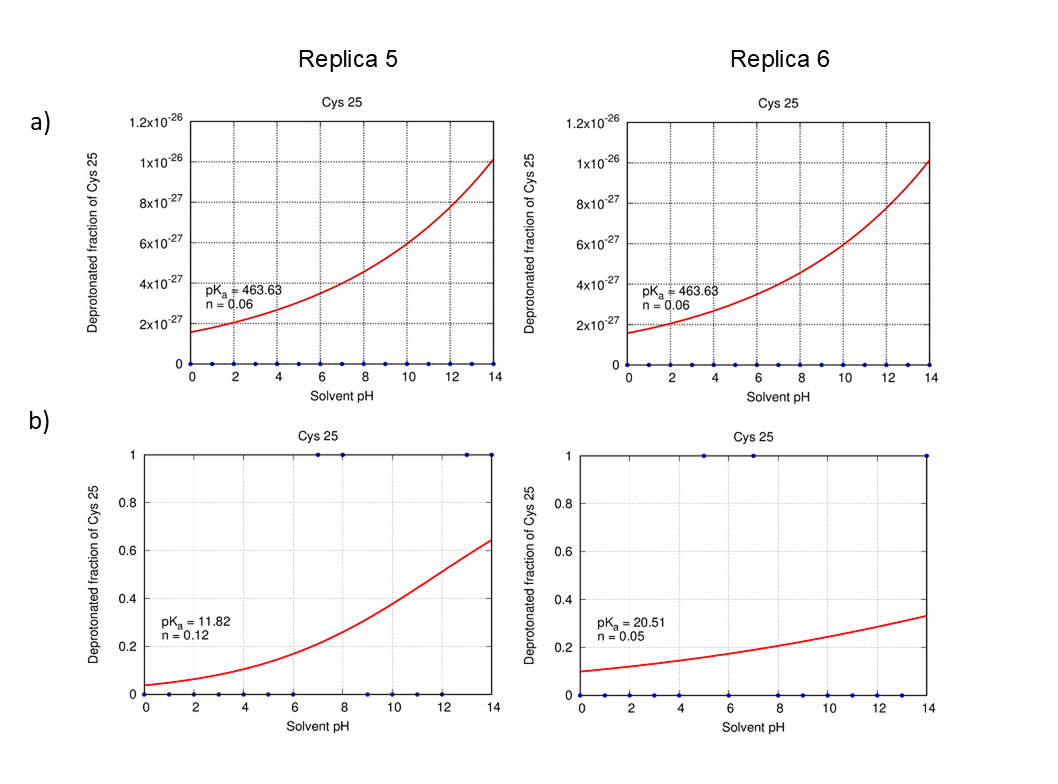


**Figure S17.** Titration curves of Cys25 in the Cz-substrate complex, minimum C. The pKa of Cys25 was evaluated considering two cases: (a) neutral His162 and (b) positively charged His162.


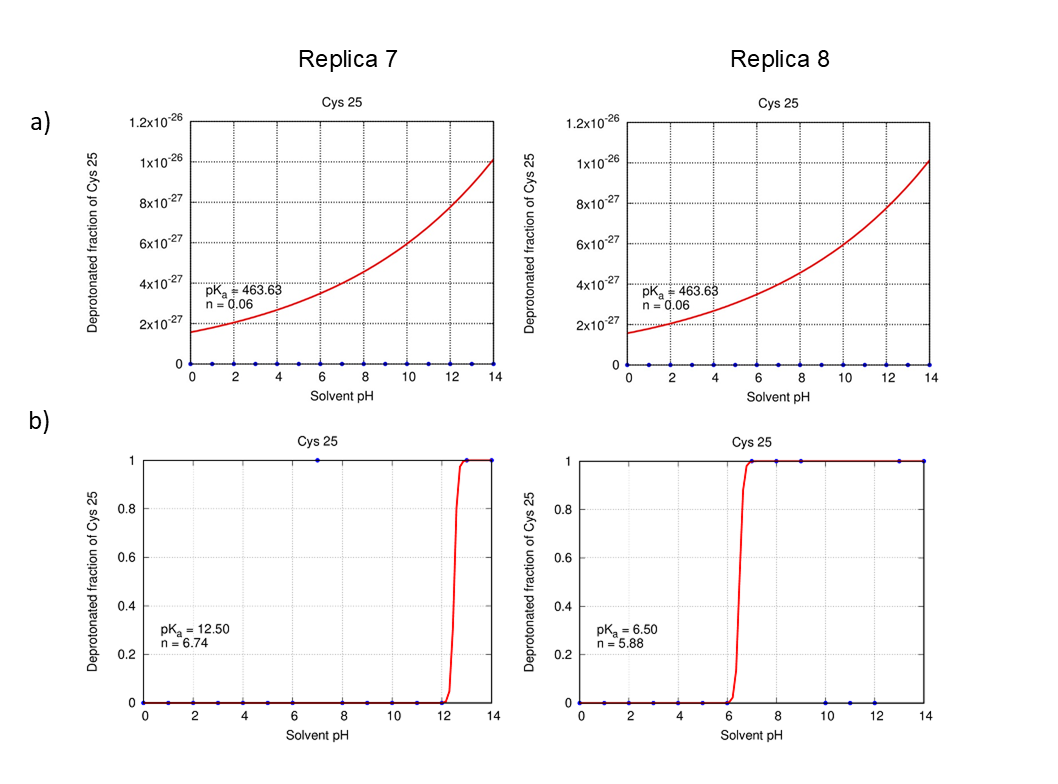


**Figure S18.** Titration curves of Cys25 in the Cz-substrate complex, minimum D. The pKa of Cys25 was evaluated considering two cases: (a) neutral His162 and (b) positively charged His162.


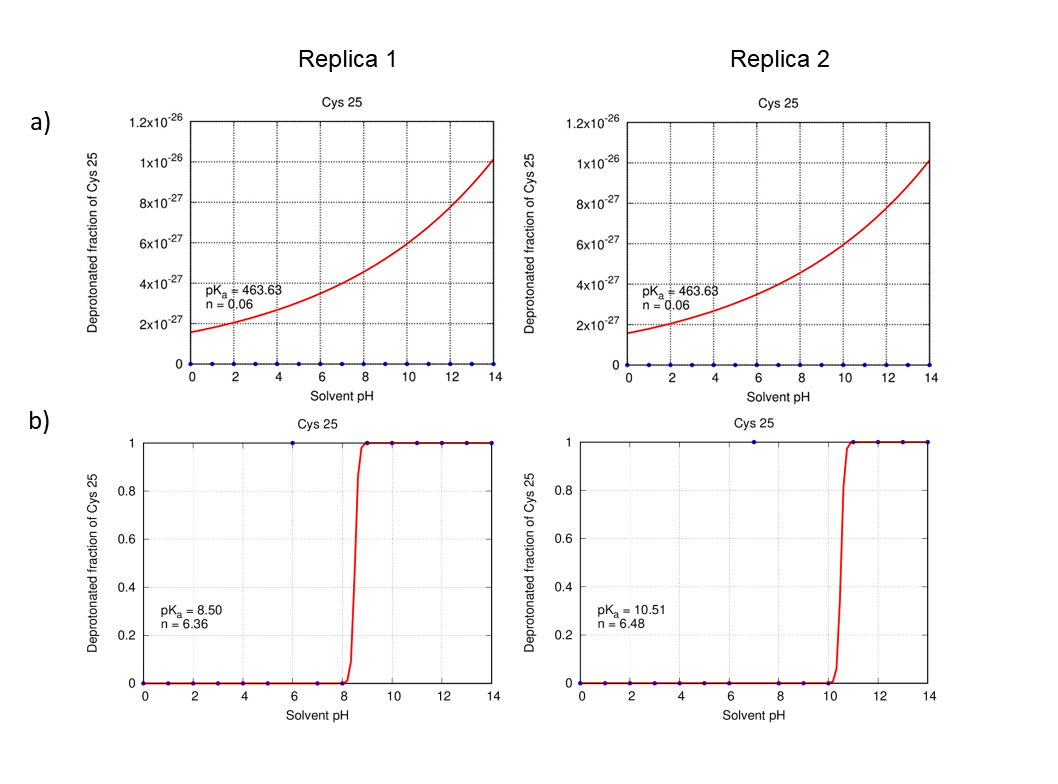


**Figure S19.** Titration curves of Cys25 in the Start point 1. The pKa of Cys25 was evaluated considering two cases: (a) neutral His162 and (b) positively charged His162.


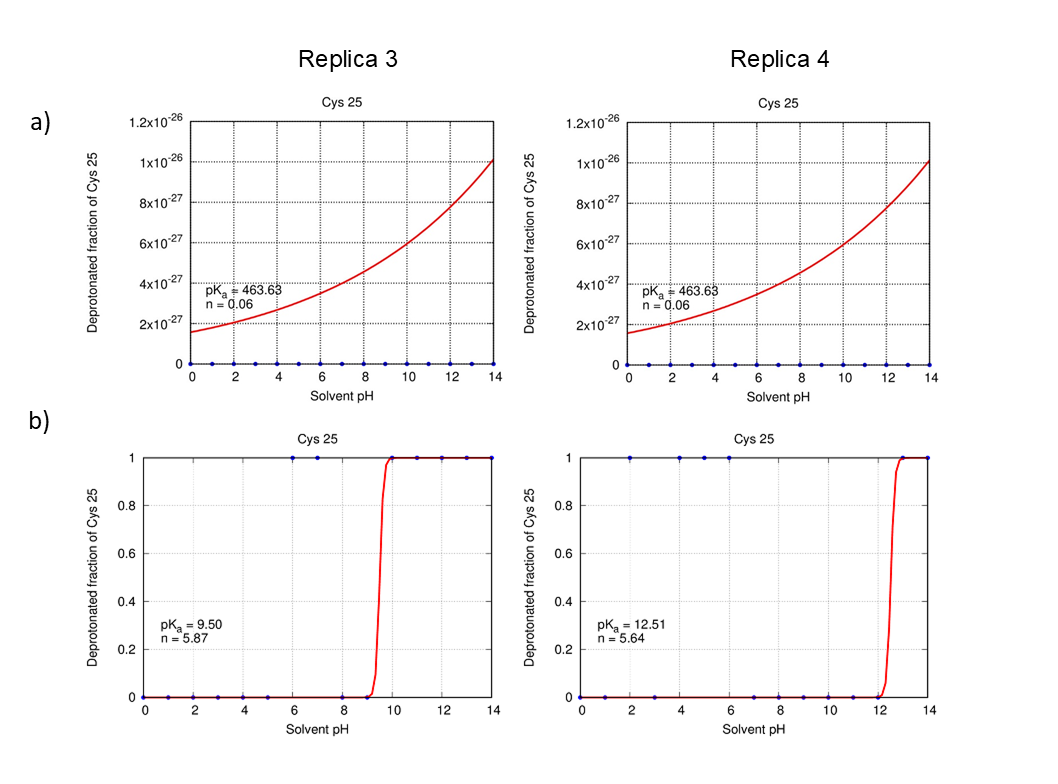


**Figure S20.** Titration curves of Cys25 in the Start point 2. The pKa of Cys25 was evaluated considering two cases: (a) neutral His162 and (b) positively charged His162.


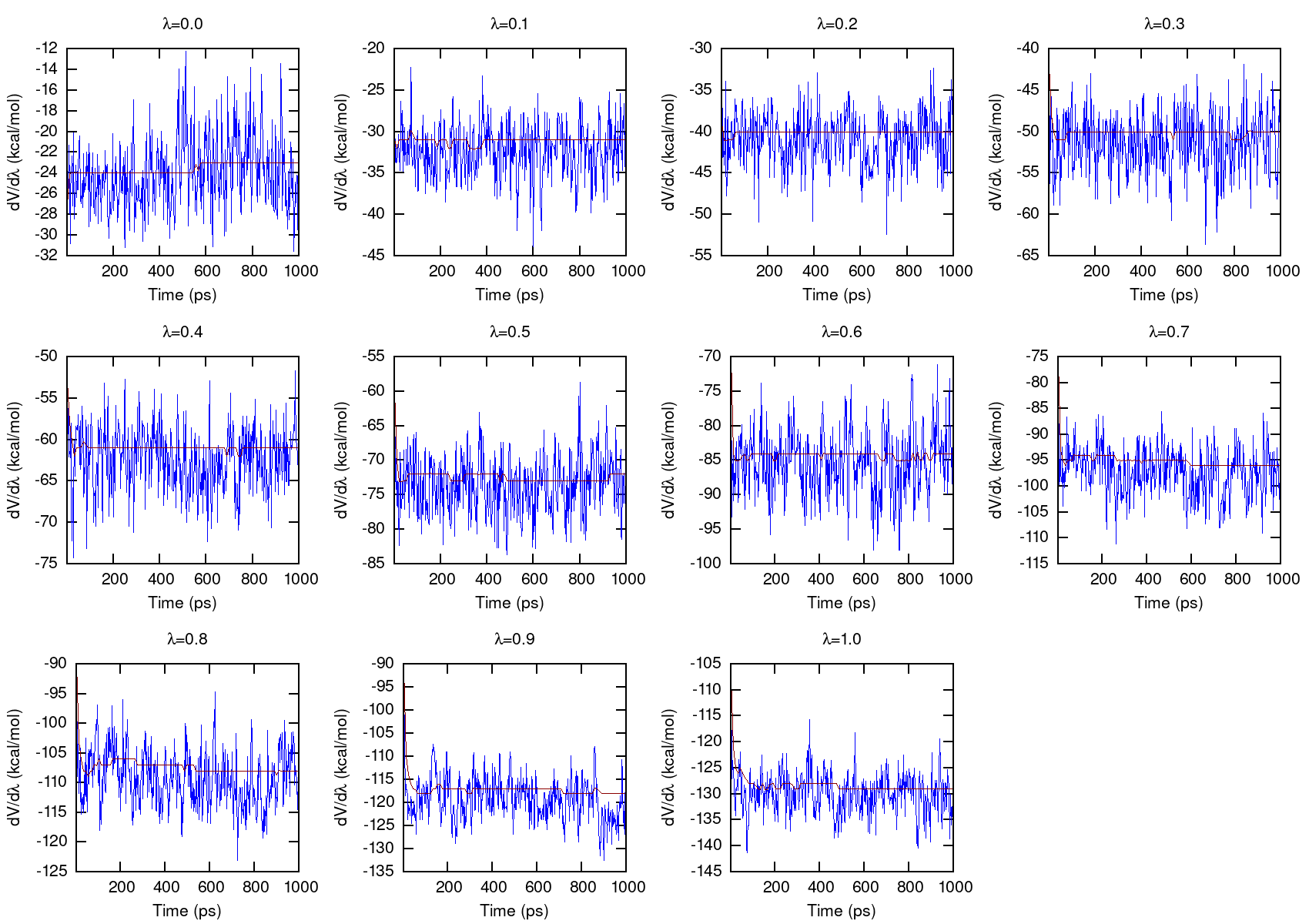


**Figure S21.** dV/dλ graphs (in kcal/mol) for Cys25 in Start point 1 (replica 1), at different values ​​of the coupling variable (λ, from 0.0 to 1.0). The blue line represents the instantaneous dV/dλ values ​​over time (up to 1000 ps), while the dark red line indicates the smoothed average of the data, obtained by spline fitting. The graphs allow evaluating the convergence and evolution of dV/dλ energy as a function of time for different values ​​of the coupling variable λ.


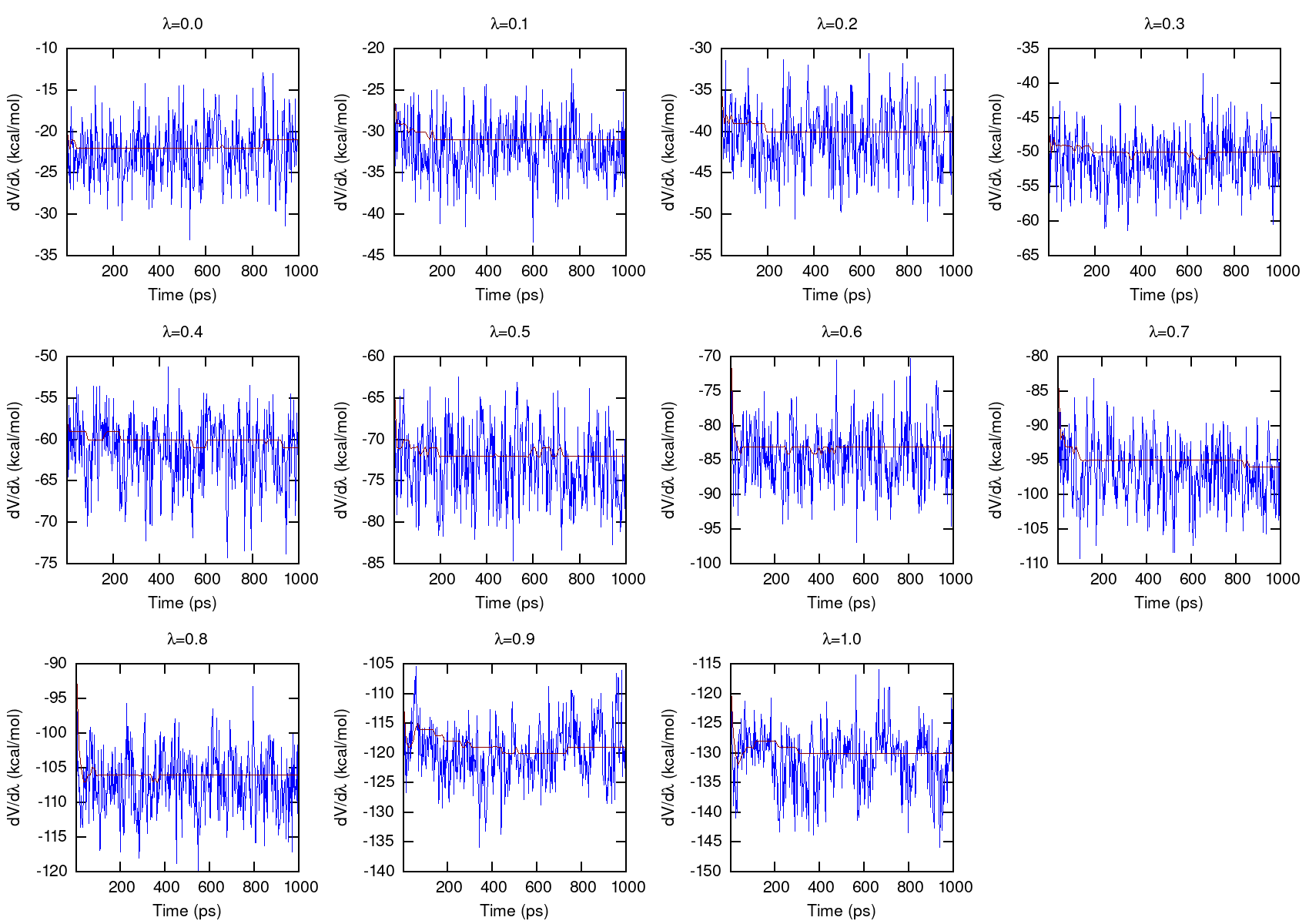


**Figure S22.** dV/dλ graphs (in kcal/mol) for Cys25 in Start point 1 (replica 2), at different values ​​of the coupling variable (λ, from 0.0 to 1.0). The blue line represents the instantaneous dV/dλ values ​​over time (up to 1000 ps), while the dark red line indicates the smoothed average of the data, obtained by spline fitting. The graphs allow evaluating the convergence and evolution of dV/dλ energy as a function of time for different values ​​of the coupling variable λ.


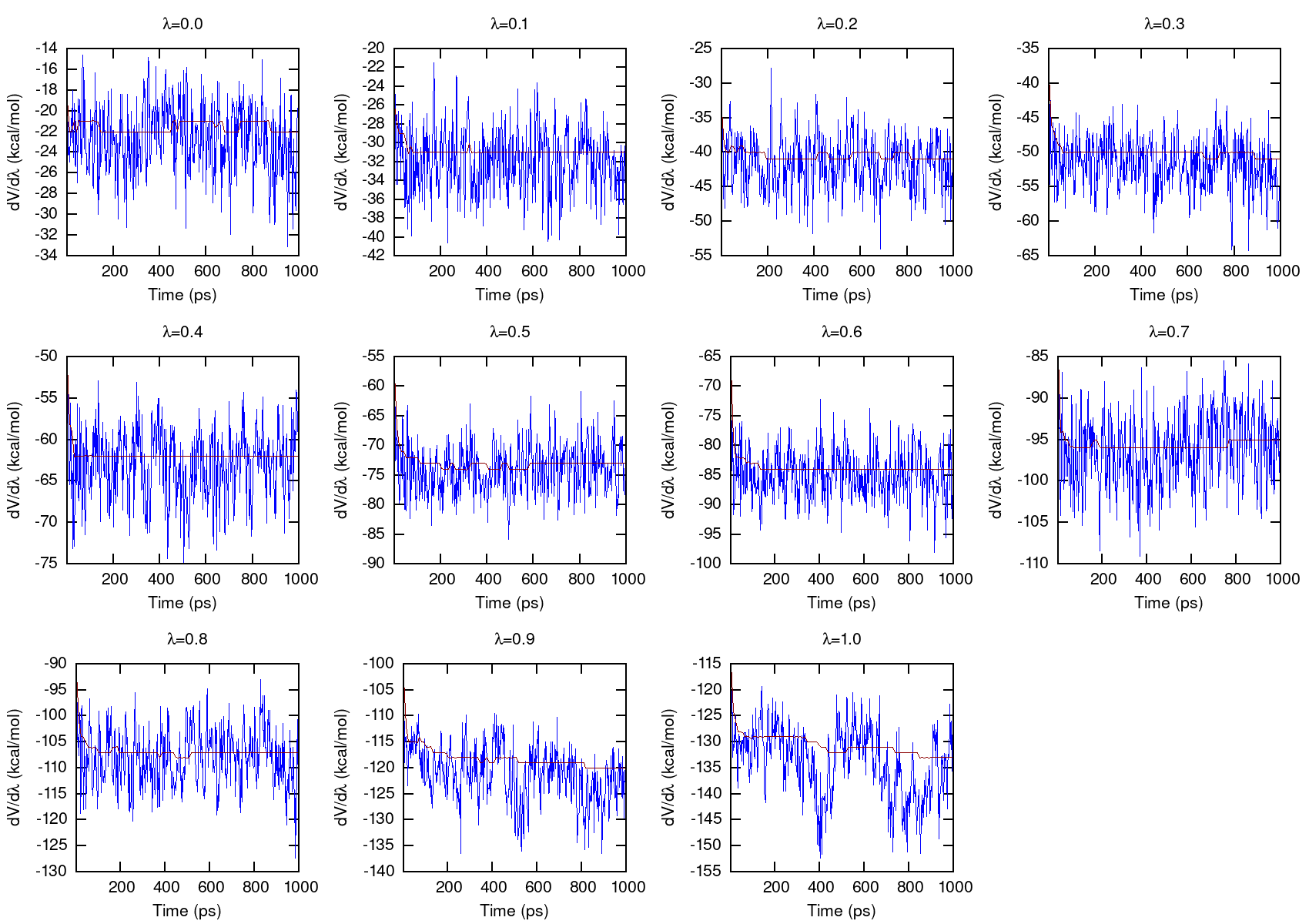


**Figure S23.** dV/dλ graphs (in kcal/mol) for Cys25 in Start point 2 (replica 3), at different values ​​of the coupling variable (λ, from 0.0 to 1.0). The blue line represents the instantaneous dV/dλ values ​​over time (up to 1000 ps), while the dark red line indicates the smoothed average of the data, obtained by spline fitting. The graphs allow evaluating the convergence and evolution of dV/dλ energy as a function of time for different values ​​of the coupling variable λ.


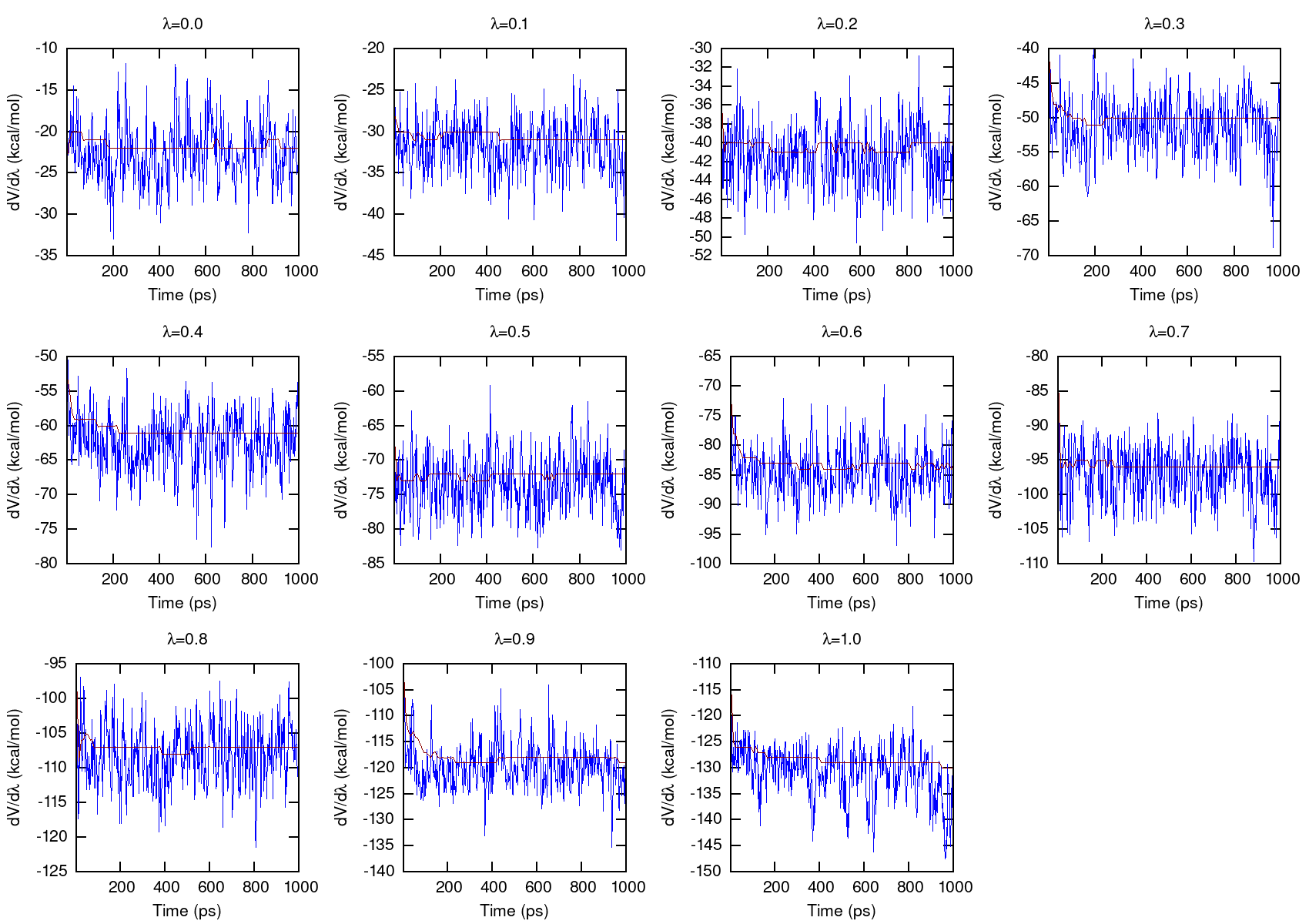


**Figure S24.** dV/dλ graphs (in kcal/mol) for Cys25 in Start point 2 (replica 4), at different values ​​of the coupling variable (λ, from 0.0 to 1.0). The blue line represents the instantaneous dV/dλ values ​​over time (up to 1000 ps), while the dark red line indicates the smoothed average of the data, obtained by spline fitting. The graphs allow evaluating the convergence and evolution of dV/dλ energy as a function of time for different values ​​of the coupling variable λ.


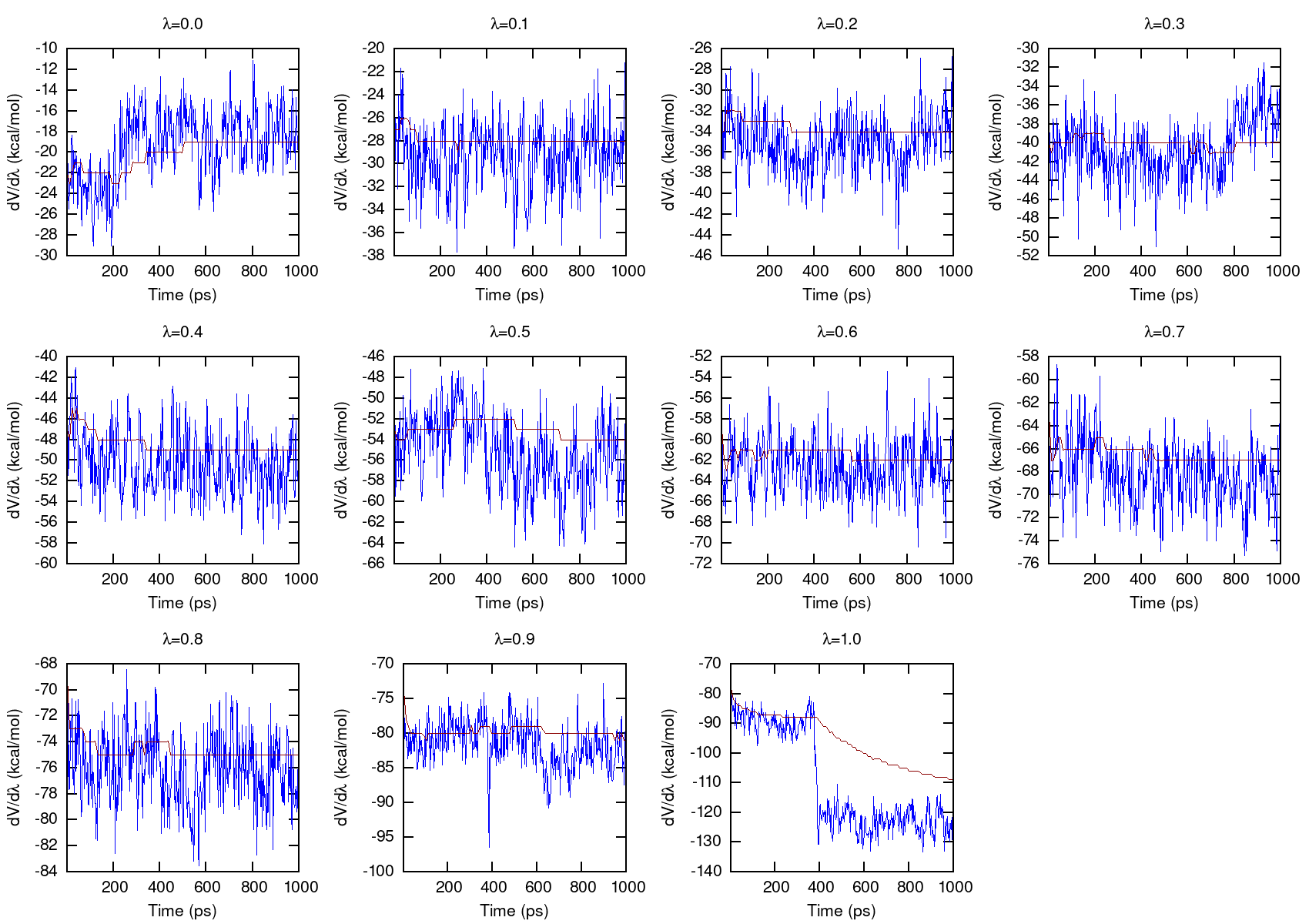


**Figure S25.** dV/dλ graphs (in kcal/mol) for Cys25 in Cz-**K777**, minimum A (replica 1), at different values ​​of the coupling variable (λ, from 0.0 to 1.0). The blue line represents the instantaneous dV/dλ values ​​over time (up to 1000 ps), while the dark red line indicates the smoothed average of the data, obtained by spline fitting. The graphs allow evaluating the convergence and evolution of dV/dλ energy as a function of time for different values ​​of the coupling variable λ.


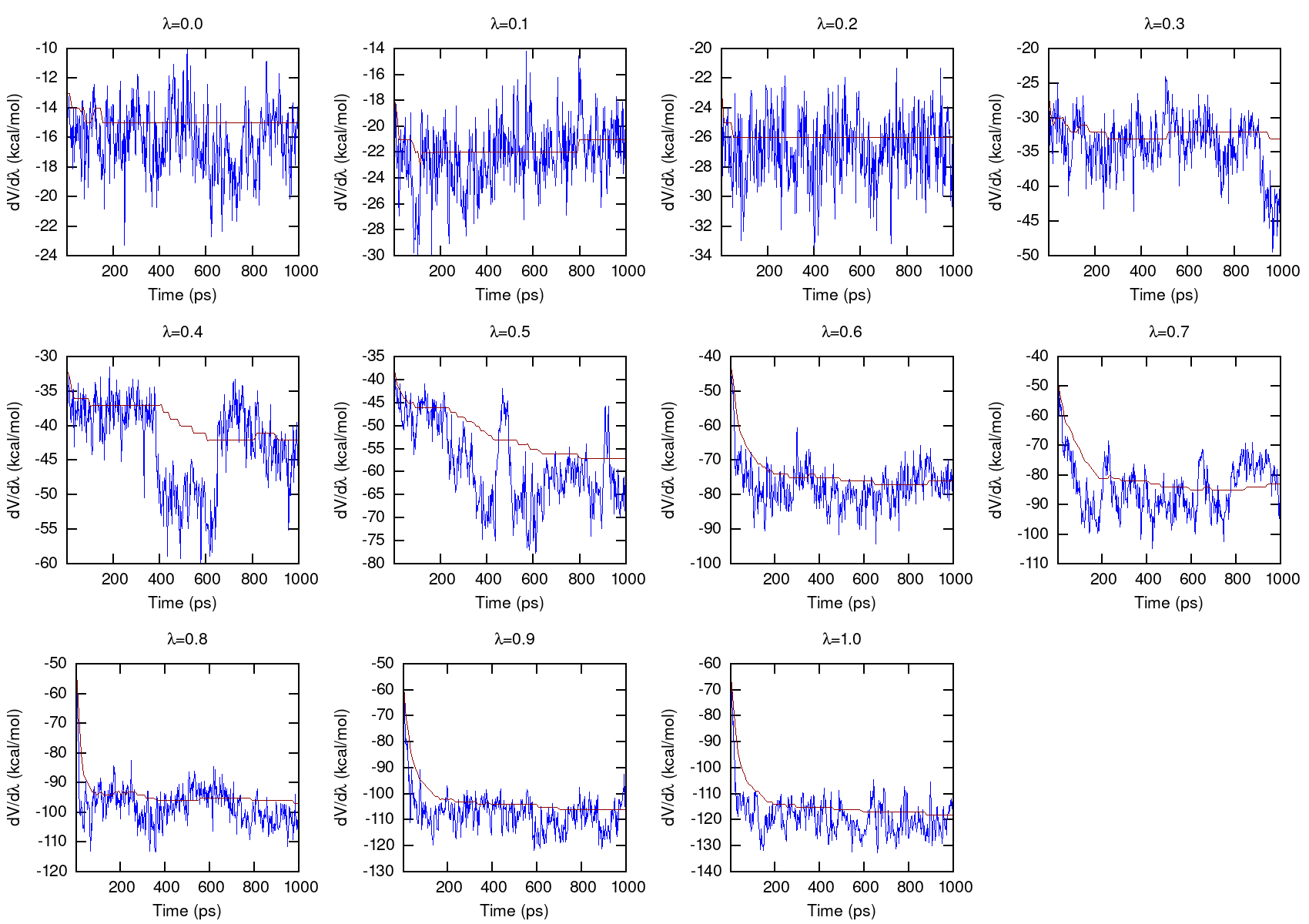


**Figure S26.** dV/dλ graphs (in kcal/mol) for Cys25 in Cz-**K777**, minimum A (replica 2), at different values ​​of the coupling variable (λ, from 0.0 to 1.0). The blue line represents the instantaneous dV/dλ values ​​over time (up to 1000 ps), while the dark red line indicates the smoothed average of the data, obtained by spline fitting. The graphs allow evaluating the convergence and evolution of dV/dλ energy as a function of time for different values ​​of the coupling variable λ.


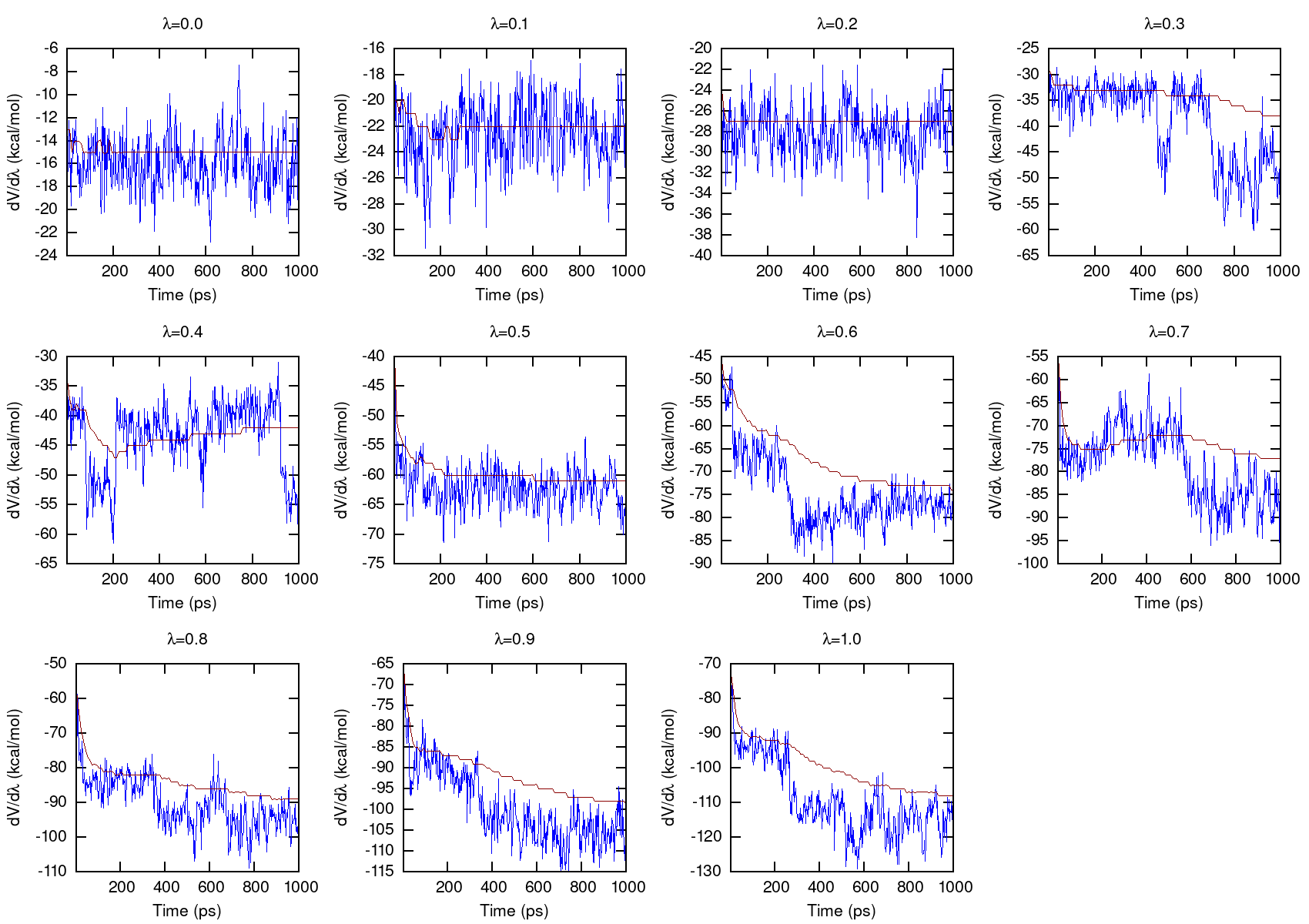


**Figure S27.** dV/dλ graphs (in kcal/mol) for Cys25 in Cz-**K777**, minimum B (replica 3), at different values ​​of the coupling variable (λ, from 0.0 to 1.0). The blue line represents the instantaneous dV/dλ values ​​over time (up to 1000 ps), while the dark red line indicates the smoothed average of the data, obtained by spline fitting. The graphs allow evaluating the convergence and evolution of dV/dλ energy as a function of time for different values ​​of the coupling variable λ.


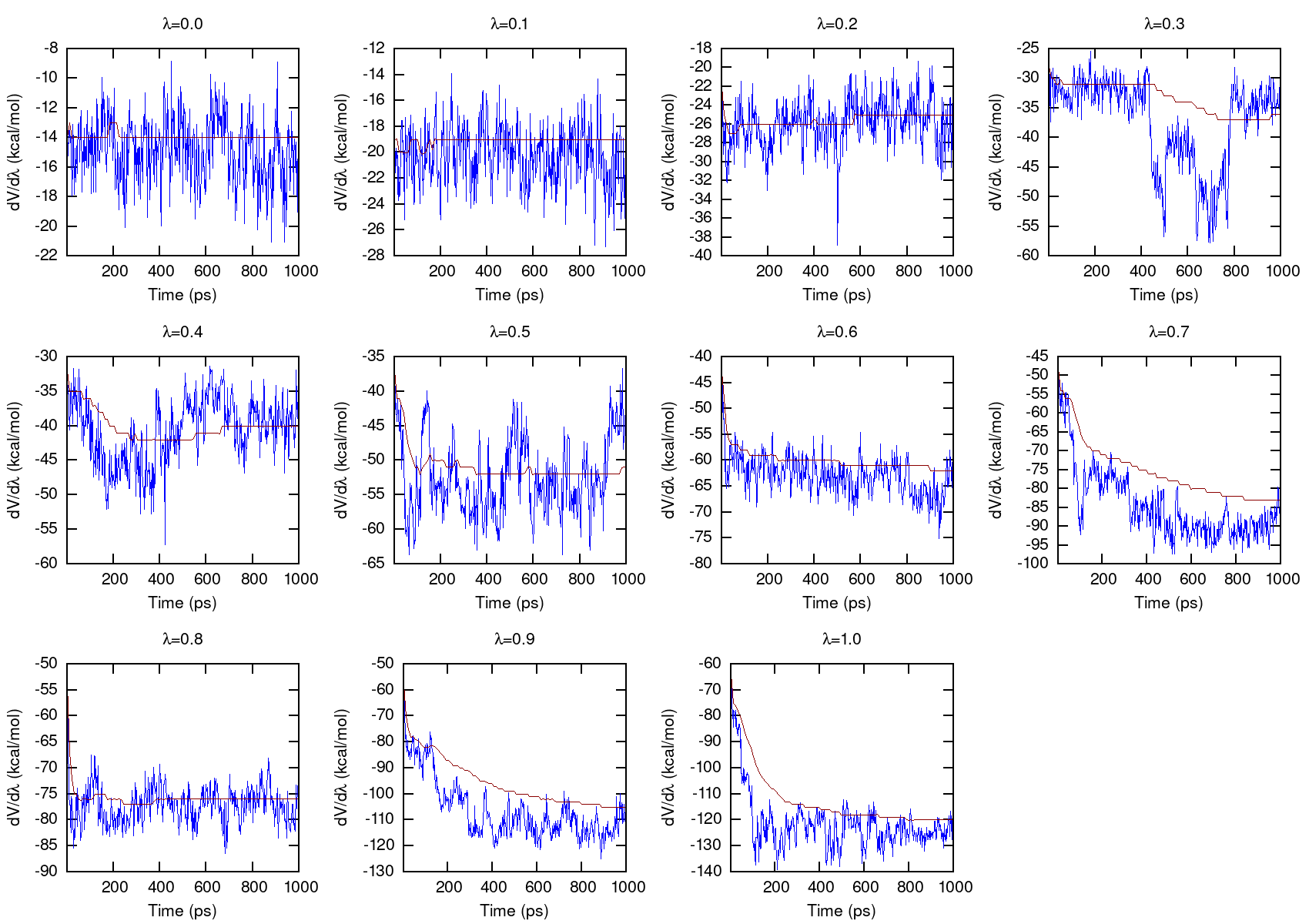


**Figure S28.** dV/dλ graphs (in kcal/mol) for Cys25 in Cz-**K777**, minimum B (replica 4), at different values ​​of the coupling variable (λ, from 0.0 to 1.0). The blue line represents the instantaneous dV/dλ values ​​over time (up to 1000 ps), while the dark red line indicates the smoothed average of the data, obtained by spline fitting. The graphs allow evaluating the convergence and evolution of dV/dλ energy as a function of time for different values ​​of the coupling variable λ.


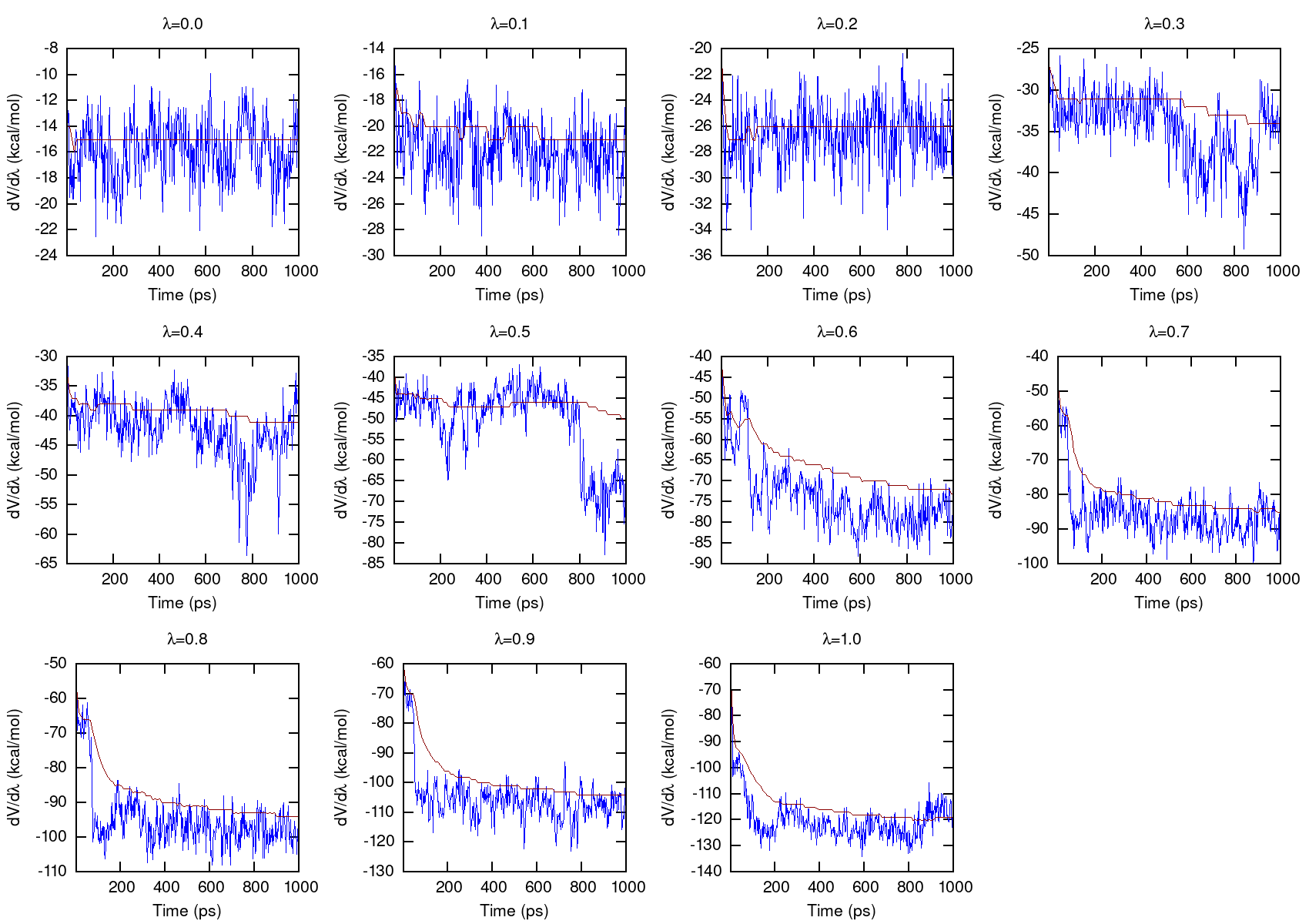


**Figure S29.** dV/dλ graphs (in kcal/mol) for Cys25 in Cz-**K777**, minimum C (replica 5), at different values ​​of the coupling variable (λ, from 0.0 to 1.0). The blue line represents the instantaneous dV/dλ values ​​over time (up to 1000 ps), while the dark red line indicates the smoothed average of the data, obtained by spline fitting. The graphs allow evaluating the convergence and evolution of dV/dλ energy as a function of time for different values ​​of the coupling variable λ.


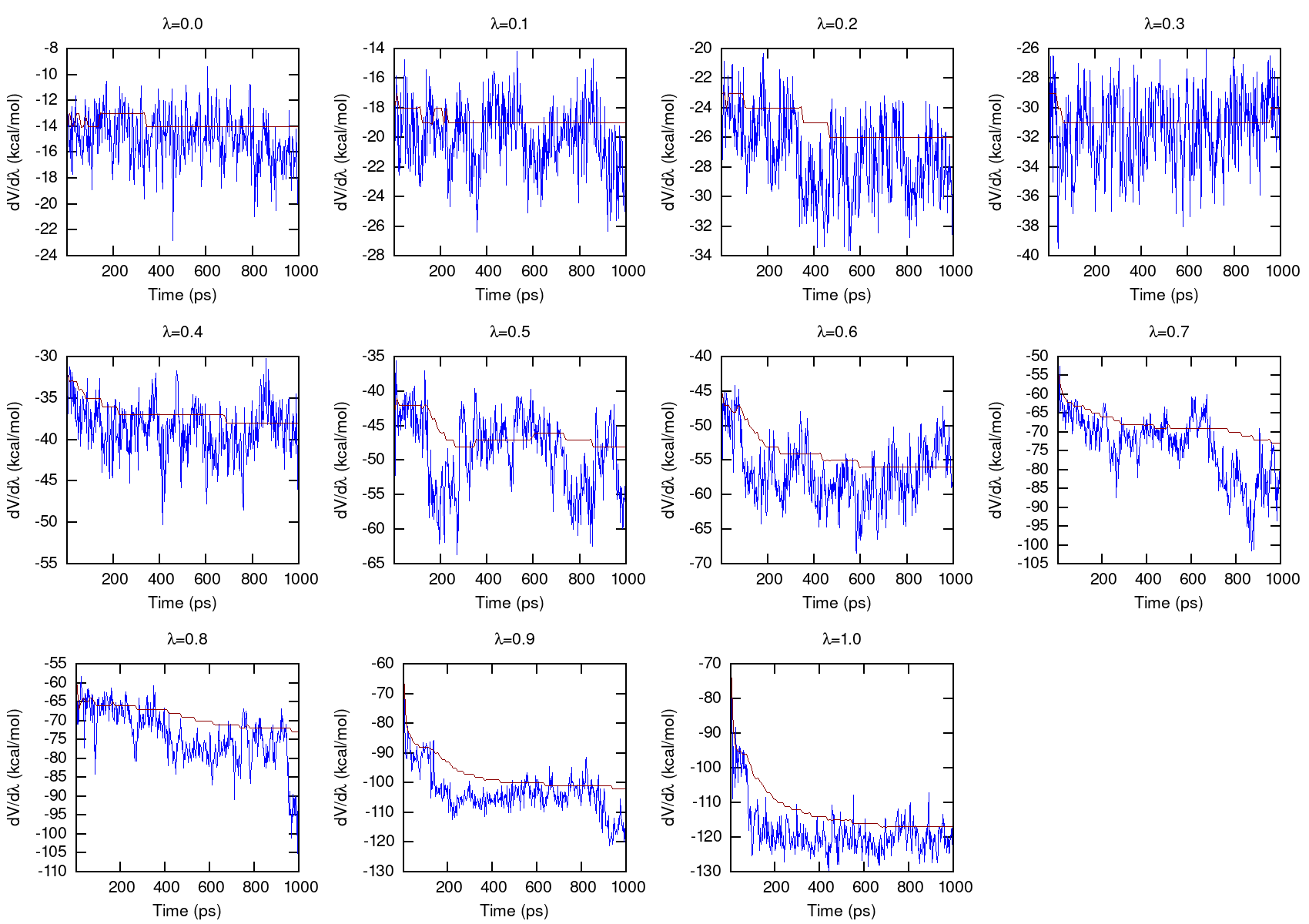


**Figure S30.** dV/dλ graphs (in kcal/mol) for Cys25 in Cz-**K777**, minimum C (replica 6), at different values ​​of the coupling variable (λ, from 0.0 to 1.0). The blue line represents the instantaneous dV/dλ values ​​over time (up to 1000 ps), while the dark red line indicates the smoothed average of the data, obtained by spline fitting. The graphs allow evaluating the convergence and evolution of dV/dλ energy as a function of time for different values ​​of the coupling variable λ.


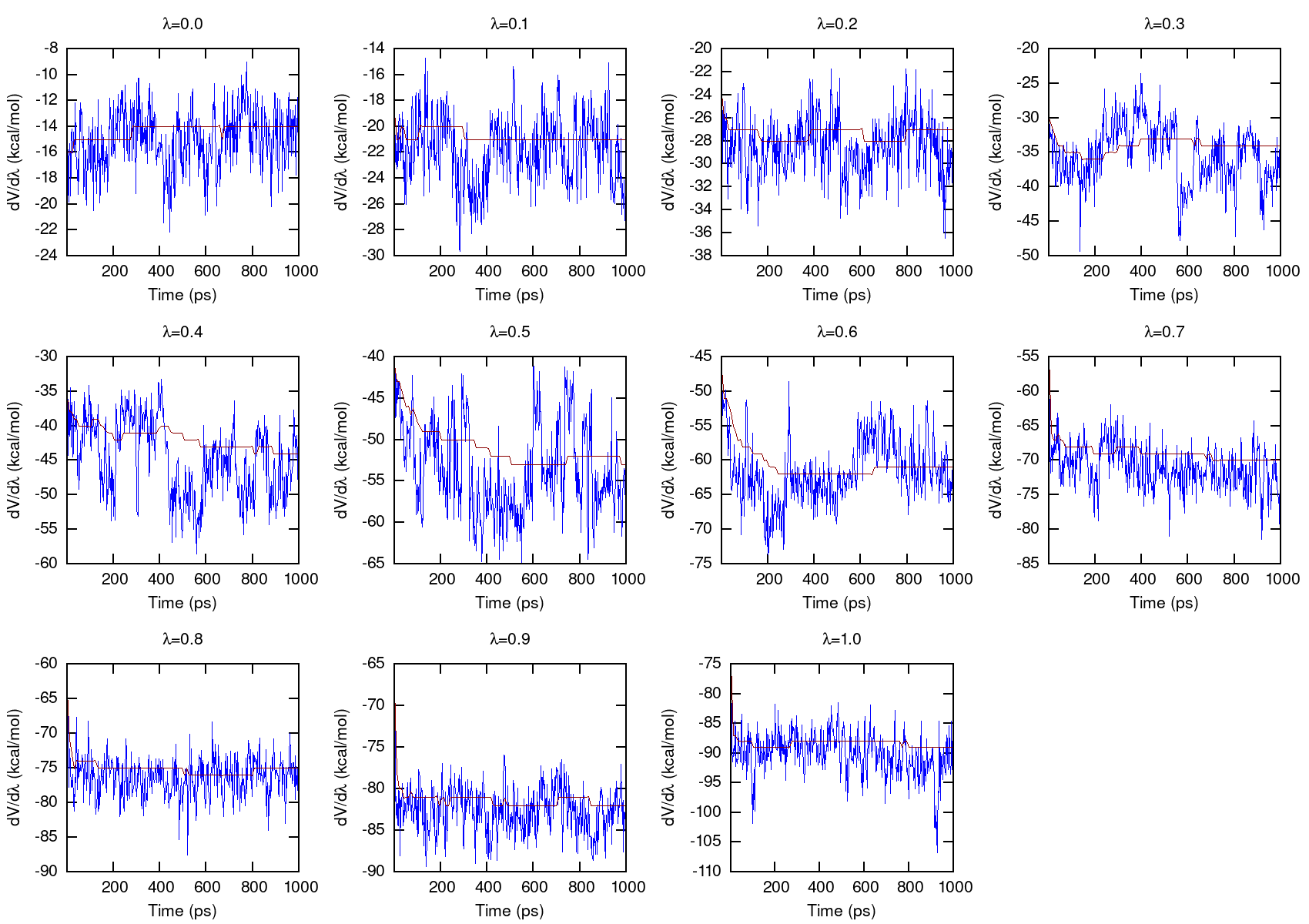


**Figure S31.** dV/dλ graphs (in kcal/mol) for Cys25 in Cz-**K777**, minimum D (replica 7), at different values ​​of the coupling variable (λ, from 0.0 to 1.0). The blue line represents the instantaneous dV/dλ values ​​over time (up to 1000 ps), while the dark red line indicates the smoothed average of the data, obtained by spline fitting. The graphs allow evaluating the convergence and evolution of dV/dλ energy as a function of time for different values ​​of the coupling variable λ.


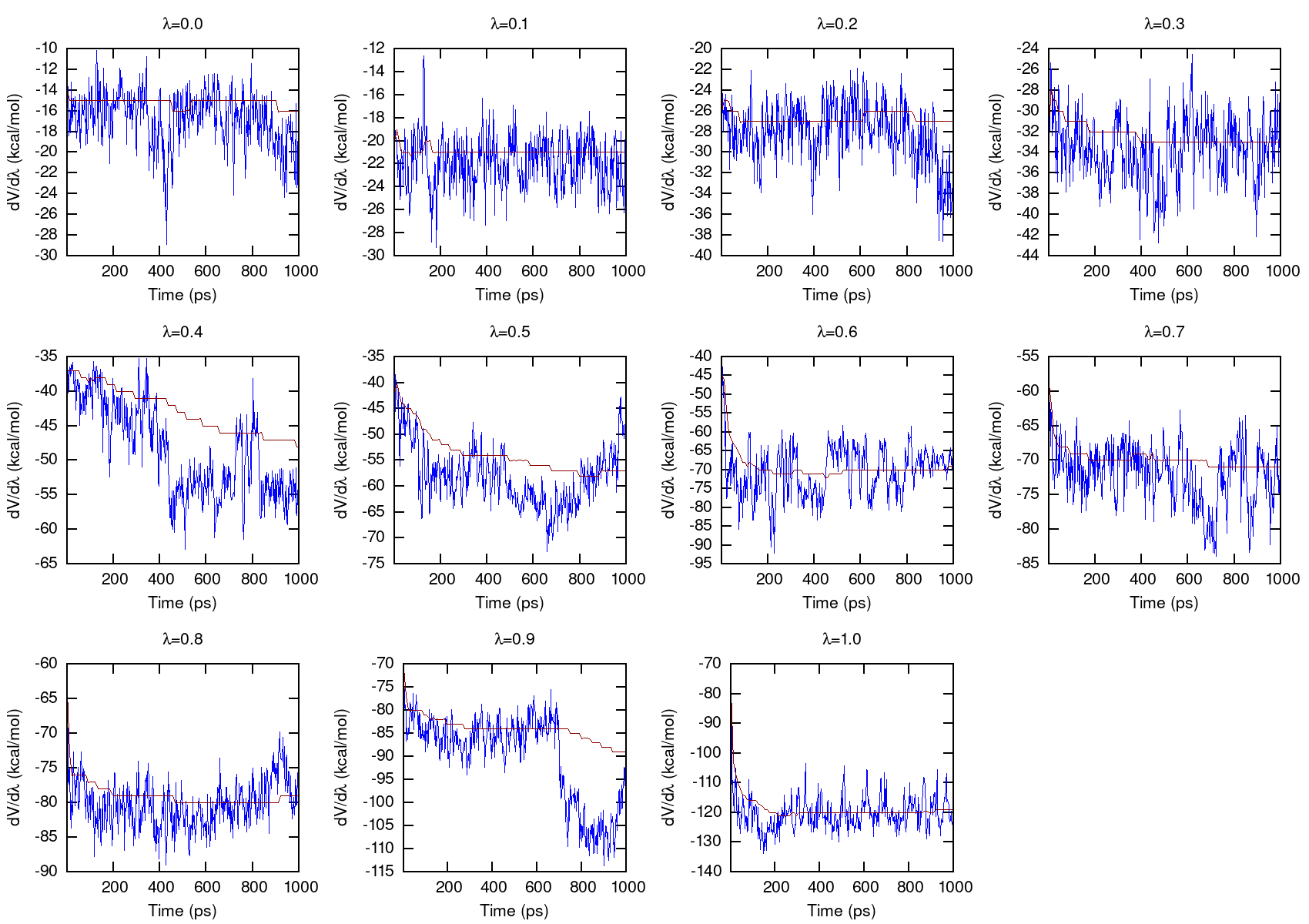


**Figure S32.** dV/dλ graphs (in kcal/mol) for Cys25 in Cz-**K777**, minimum D (replica 8), at different values ​​of the coupling variable (λ, from 0.0 to 1.0). The blue line represents the instantaneous dV/dλ values ​​over time (up to 1000 ps), while the dark red line indicates the smoothed average of the data, obtained by spline fitting. The graphs allow evaluating the convergence and evolution of dV/dλ energy as a function of time for different values ​​of the coupling variable λ.


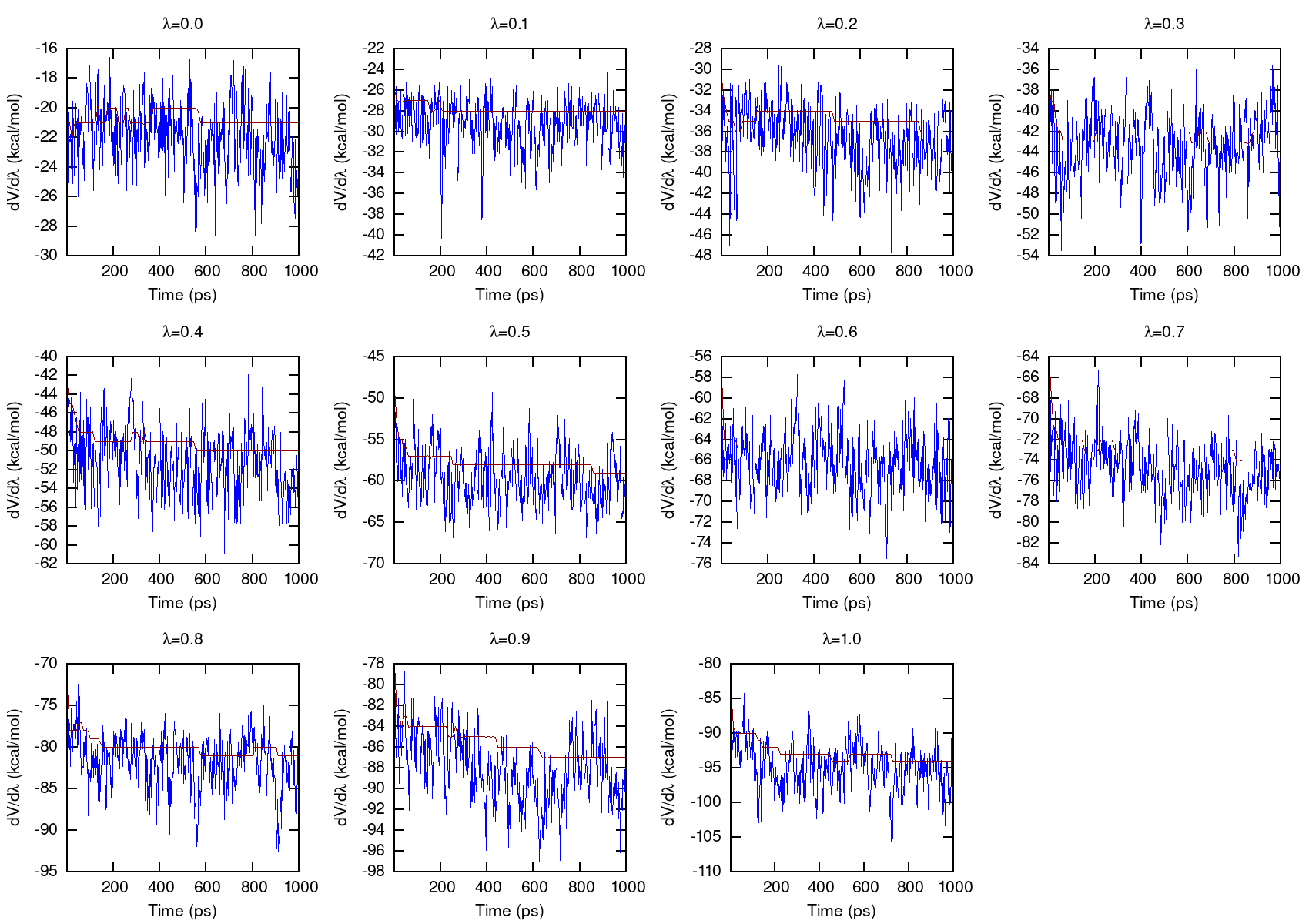


**Figure S33.** dV/dλ graphs (in kcal/mol) for Cys25 in Cz-substrate, minimum A (replica 1), at different values ​​of the coupling variable (λ, from 0.0 to 1.0). The blue line represents the instantaneous dV/dλ values ​​over time (up to 1000 ps), while the dark red line indicates the smoothed average of the data, obtained by spline fitting. The graphs allow evaluating the convergence and evolution of dV/dλ energy as a function of time for different values ​​of the coupling variable λ.


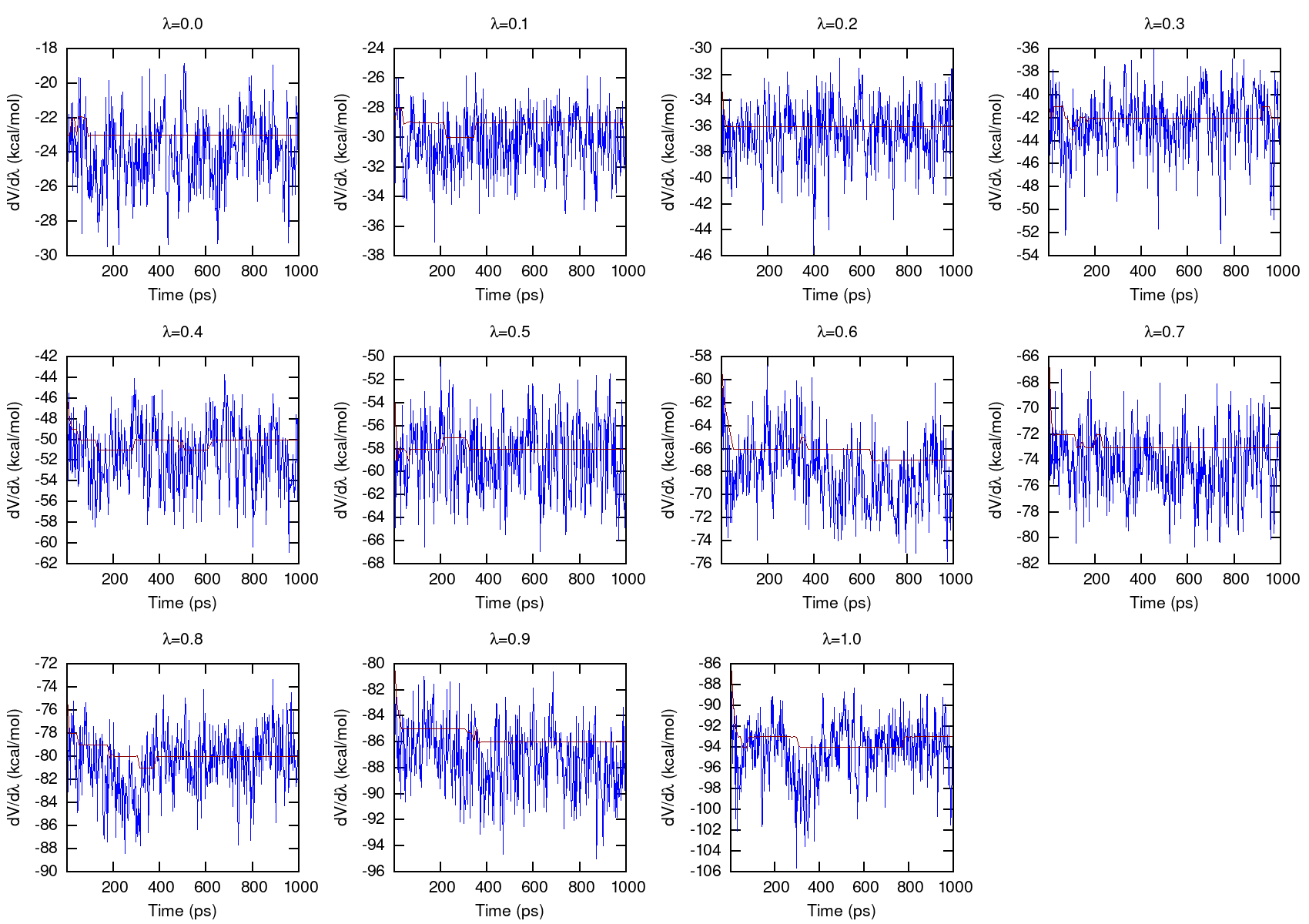


**Figure S34.** dV/dλ graphs (in kcal/mol) for Cys25 in Cz-substrate, minimum A (replica 2), at different values ​​of the coupling variable (λ, from 0.0 to 1.0). The blue line represents the instantaneous dV/dλ values ​​over time (up to 1000 ps), while the dark red line indicates the smoothed average of the data, obtained by spline fitting. The graphs allow evaluating the convergence and evolution of dV/dλ energy as a function of time for different values ​​of the coupling variable λ.


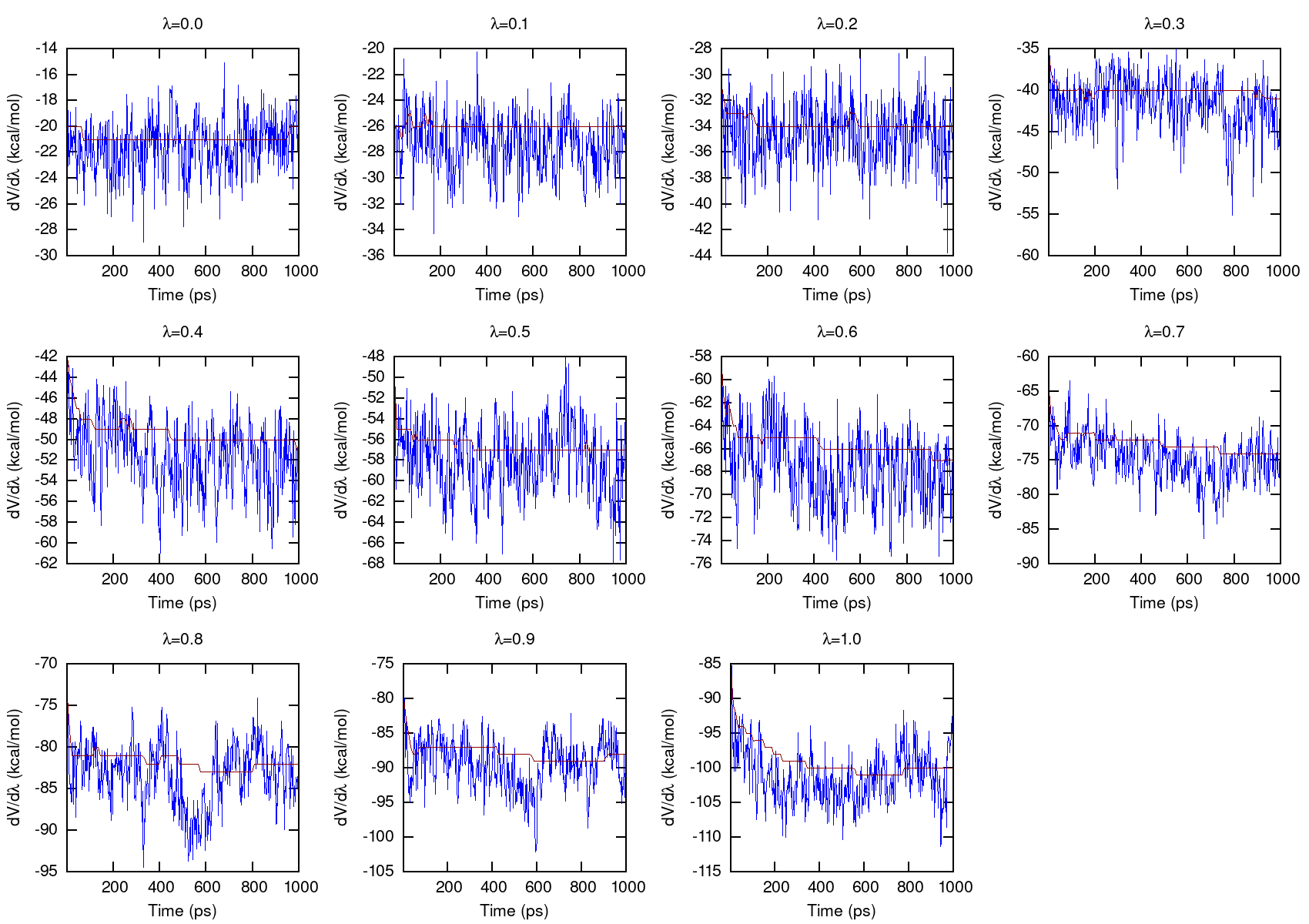


**Figure S35.** dV/dλ graphs (in kcal/mol) for Cys25 in Cz-substrate, minimum B (replica 3), at different values ​​of the coupling variable (λ, from 0.0 to 1.0). The blue line represents the instantaneous dV/dλ values ​​over time (up to 1000 ps), while the dark red line indicates the smoothed average of the data, obtained by spline fitting. The graphs allow evaluating the convergence and evolution of dV/dλ energy as a function of time for different values ​​of the coupling variable λ.


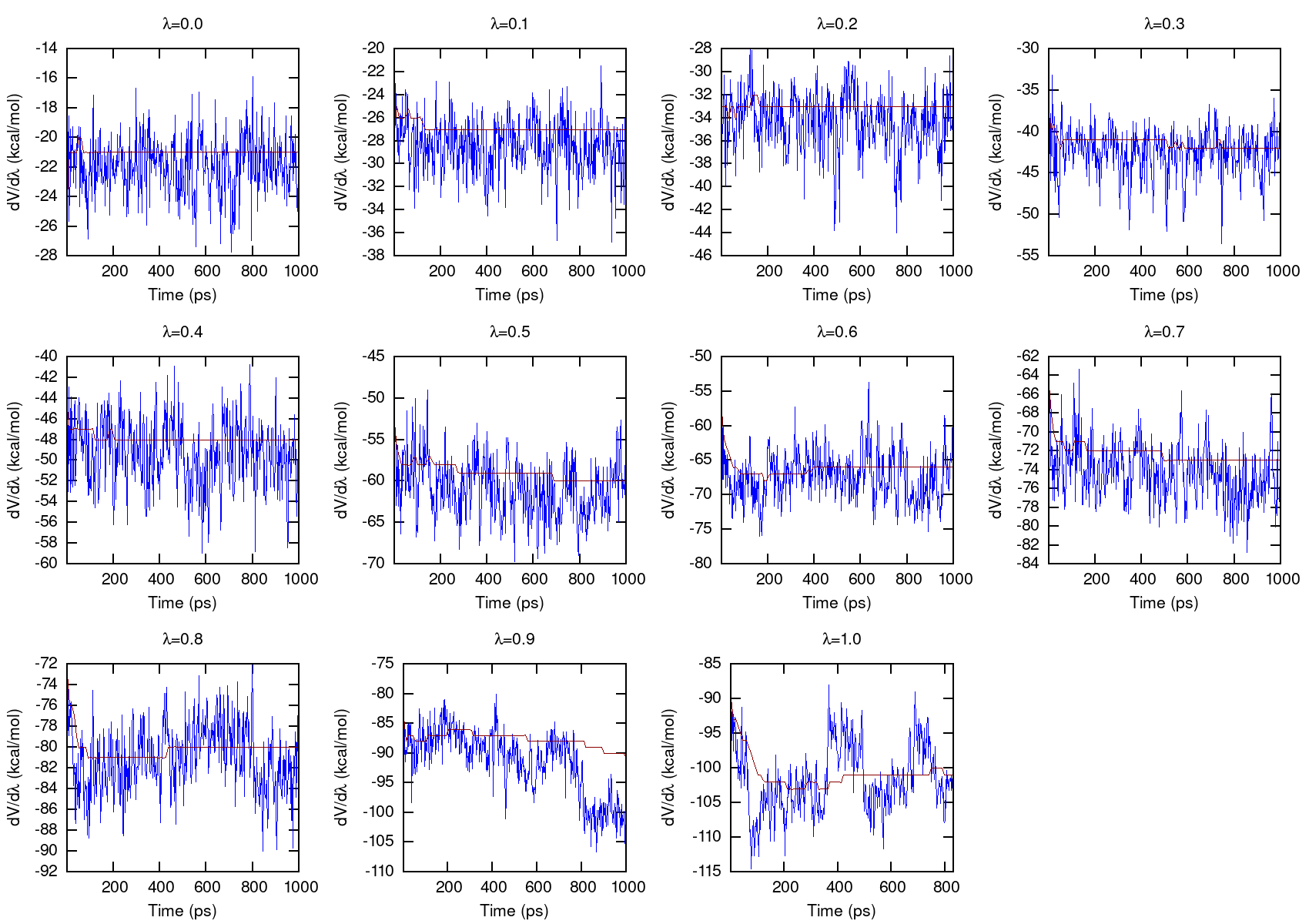


**Figure S36.** dV/dλ graphs (in kcal/mol) for Cys25 in Cz-substrate, minimum B (replica 4), at different values ​​of the coupling variable (λ, from 0.0 to 1.0). The blue line represents the instantaneous dV/dλ values ​​over time (up to 1000 ps), while the dark red line indicates the smoothed average of the data, obtained by spline fitting. The graphs allow evaluating the convergence and evolution of dV/dλ energy as a function of time for different values ​​of the coupling variable λ.


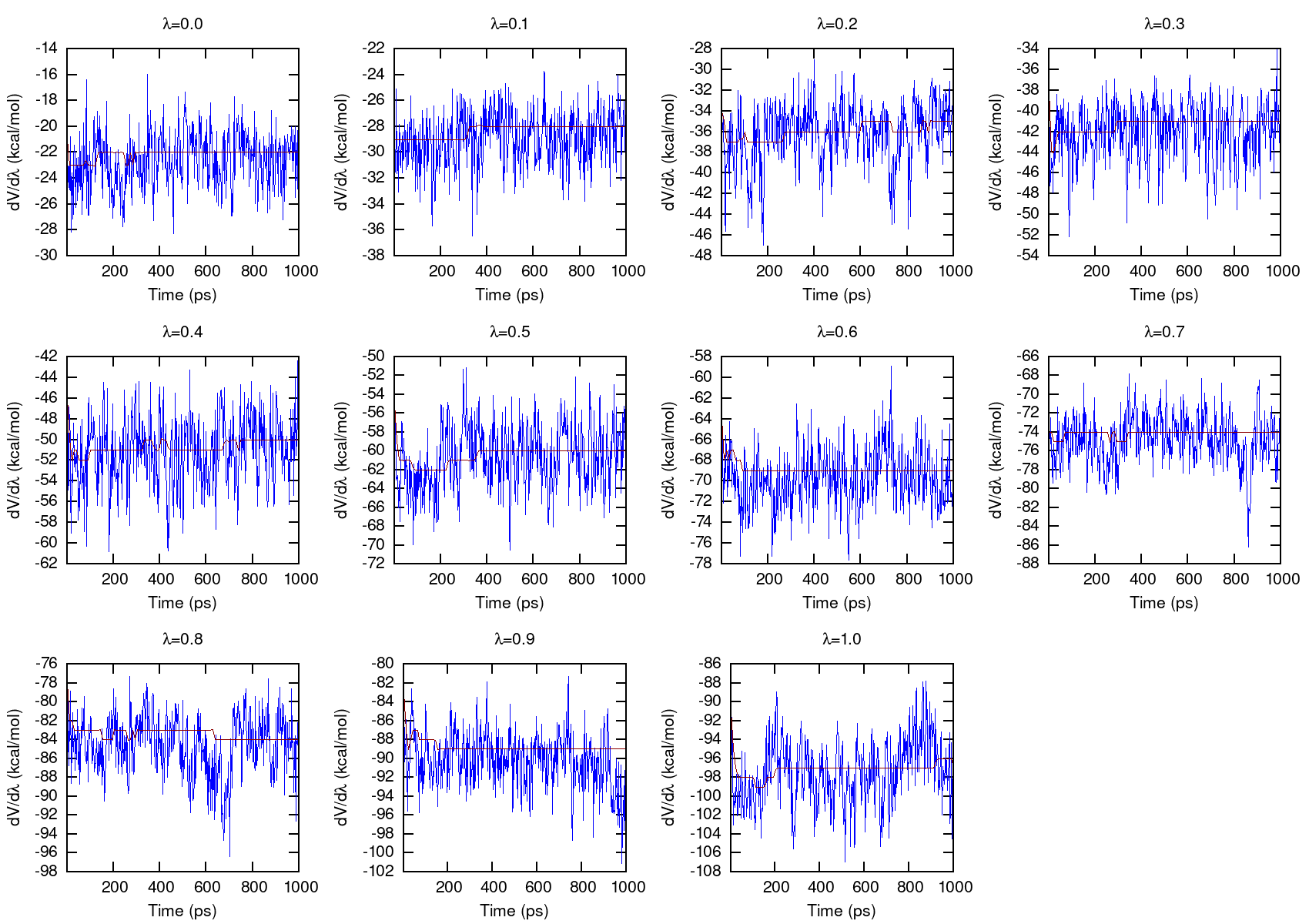


**Figure S37.** dV/dλ graphs (in kcal/mol) for Cys25 in Cz-substrate, minimum C (replica 5), at different values ​​of the coupling variable (λ, from 0.0 to 1.0). The blue line represents the instantaneous dV/dλ values ​​over time (up to 1000 ps), while the dark red line indicates the smoothed average of the data, obtained by spline fitting. The graphs allow evaluating the convergence and evolution of dV/dλ energy as a function of time for different values ​​of the coupling variable λ.


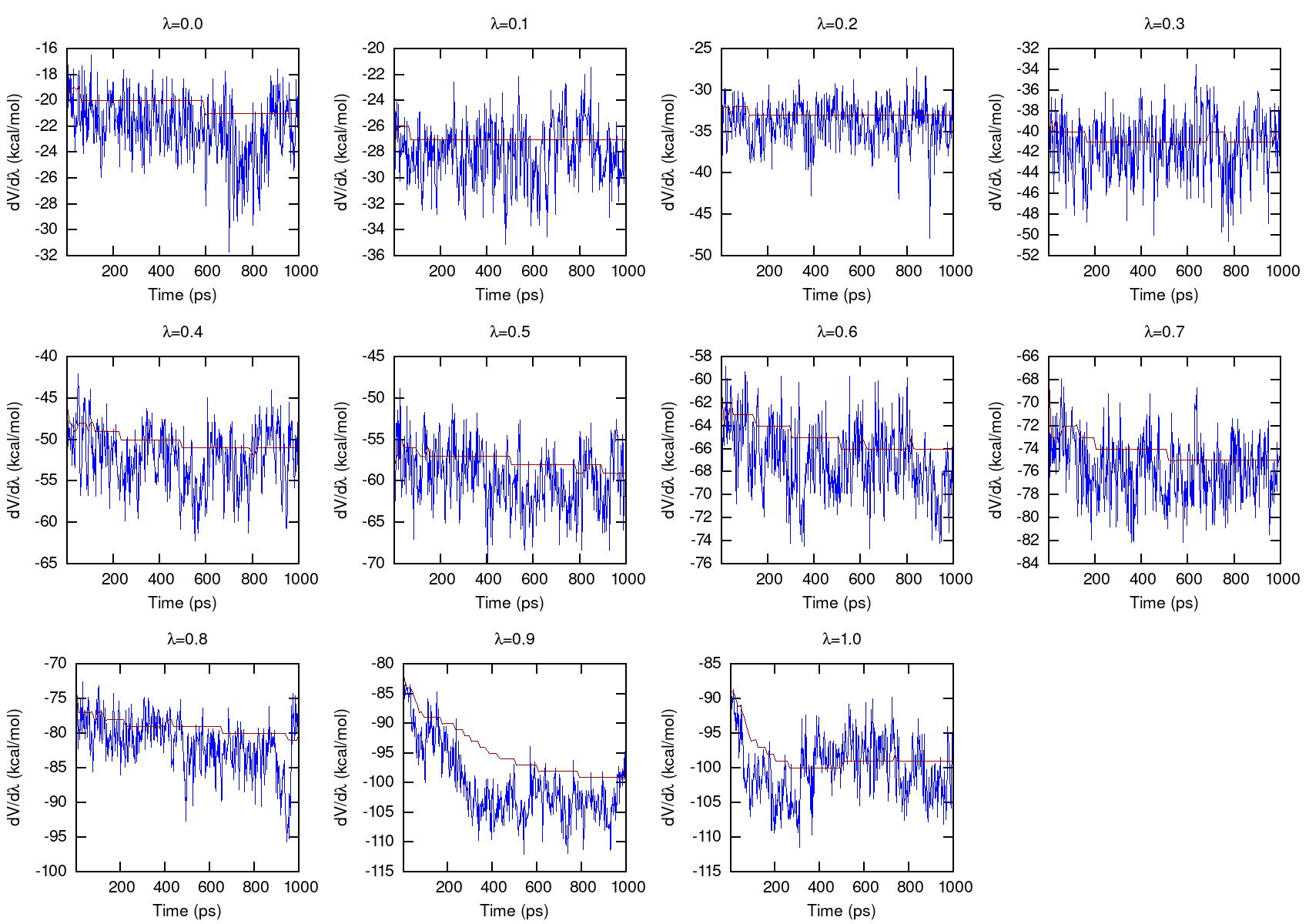


**Figure S38.** dV/dλ graphs (in kcal/mol) for Cys25 in Cz-substrate, minimum C (replica 6), at different values ​​of the coupling variable (λ, from 0.0 to 1.0). The blue line represents the instantaneous dV/dλ values ​​over time (up to 1000 ps), while the dark red line indicates the smoothed average of the data, obtained by spline fitting. The graphs allow evaluating the convergence and evolution of dV/dλ energy as a function of time for different values ​​of the coupling variable λ.


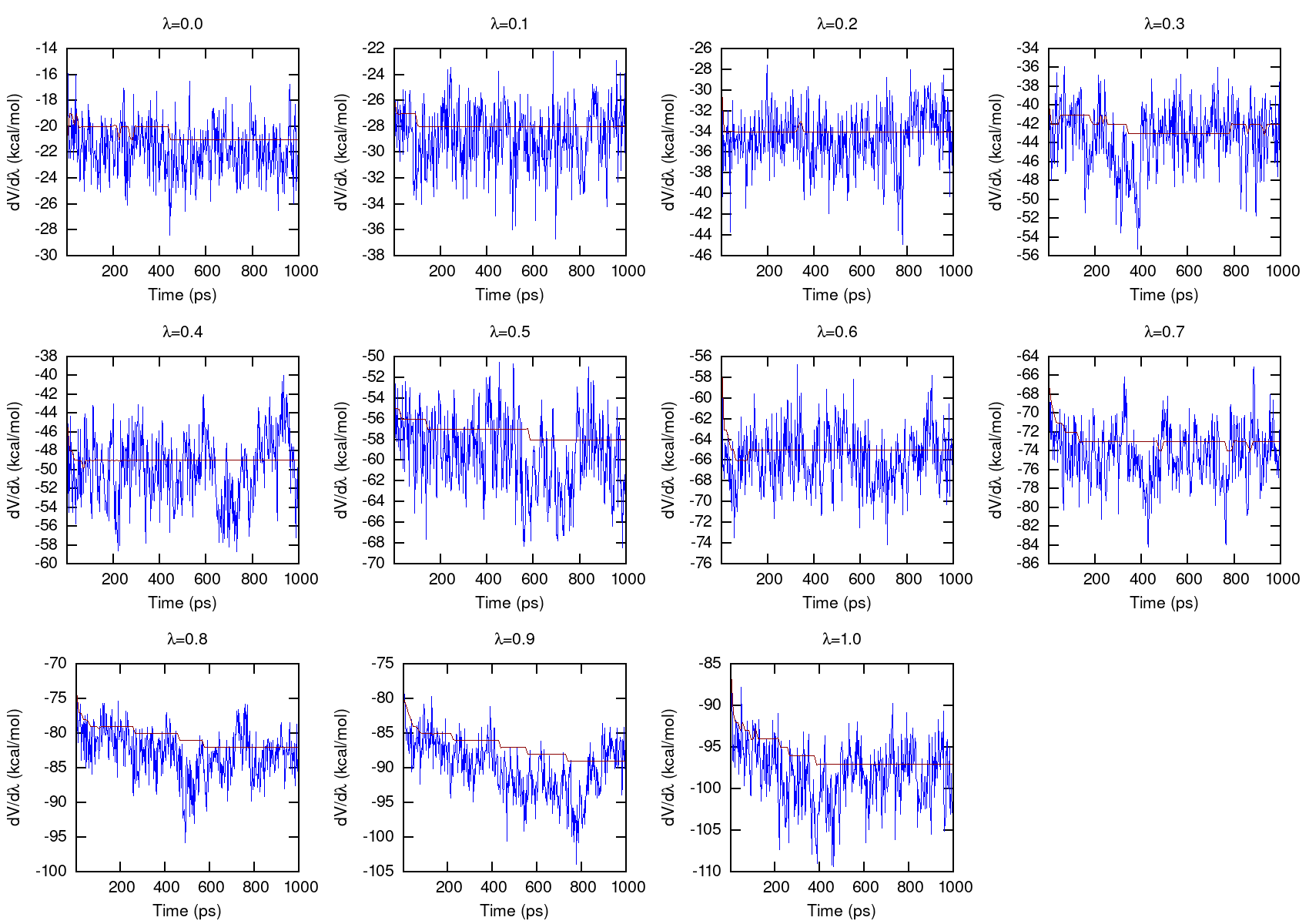


**Figure S39.** dV/dλ graphs (in kcal/mol) for Cys25 in Cz-substrate, minimum D (replica 7), at different values ​​of the coupling variable (λ, from 0.0 to 1.0). The blue line represents the instantaneous dV/dλ values ​​over time (up to 1000 ps), while the dark red line indicates the smoothed average of the data, obtained by spline fitting. The graphs allow evaluating the convergence and evolution of dV/dλ energy as a function of time for different values ​​of the coupling variable λ.


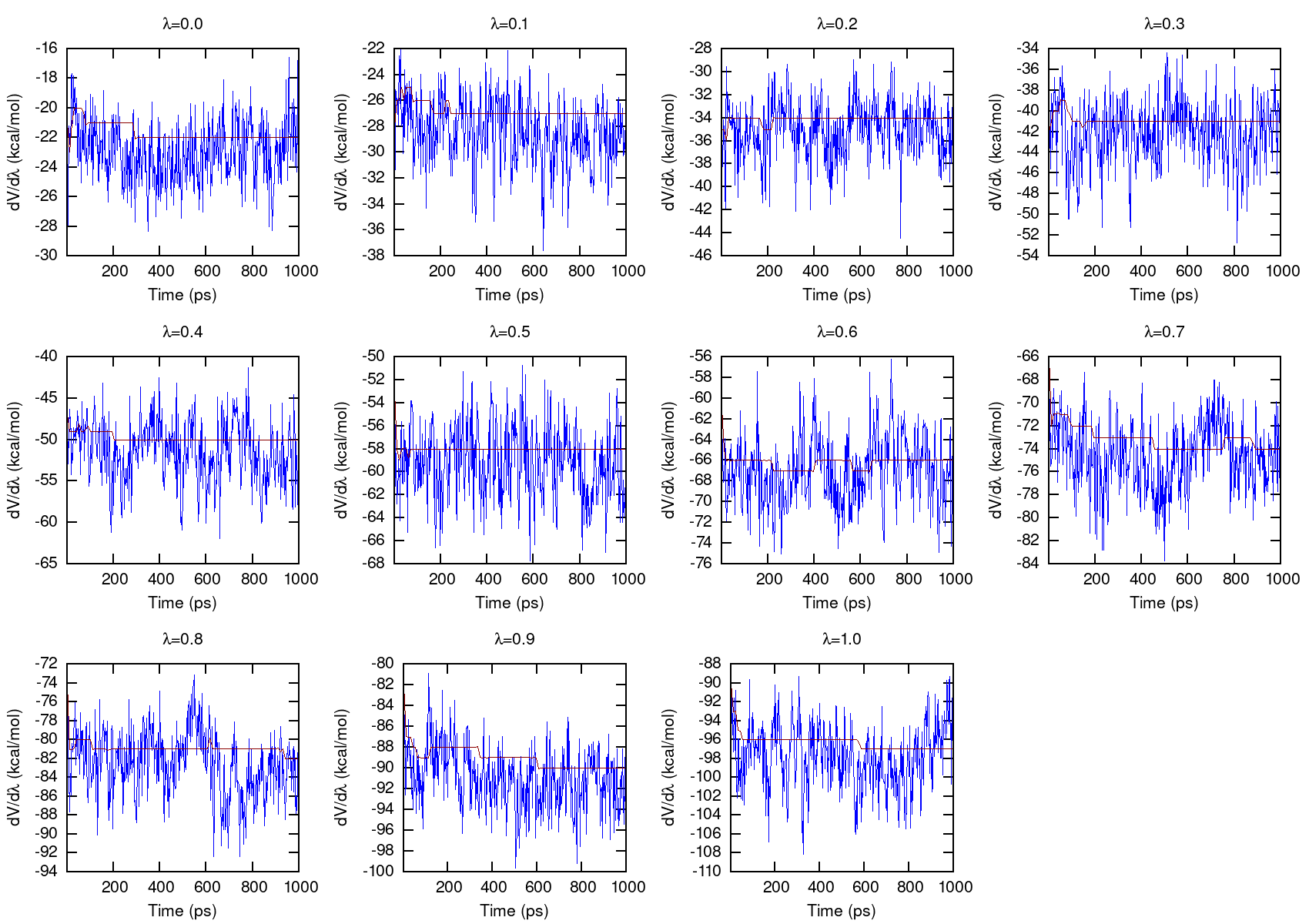


**Figure S40.** dV/dλ graphs (in kcal/mol) for Cys25 in Cz-substrate, minimum D (replica 8), at different values ​​of the coupling variable (λ, from 0.0 to 1.0). The blue line represents the instantaneous dV/dλ values ​​over time (up to 1000 ps), while the dark red line indicates the smoothed average of the data, obtained by spline fitting. The graphs allow evaluating the convergence and evolution of dV/dλ energy as a function of time for different values ​​of the coupling variable λ.

**Table S2***.* Average pKa of Cys25 in the Cz-**K777**, Cz-substrate and Cz-free form systems, including their respective replicates. The pKa of Cys25 was evaluated considering His162 in the positively charged form. For free cruzain, four replicates were performed, based on the structural starting points called Start points 1 and 2. For the cruzain-inhibitor (Cz-K777) and cruzain-substrate complexes, eight replicates were performed for each system, based on the structures corresponding to minimum-energy structures A, B, C and D*.* At Start point 1, Cys25 acts as a hydrogen bond (HB) acceptor for W598 and an HB donor for W561; at Start point 2, Cys25 acts as a HB donor for W598 and an HB acceptor for W561. Structures A, B, C, D and E represent minima identified from the analysis of free energy graphs (FEL). The energy minimum E was not analyzed, as it is associated with a system whose initial structure contains an ion pair. The simulation time for each replica correspond to 300 ns. Start points 1, 2 and 3 are described in Figure 2 of the manuscript.

|  | **pKa** |  |
| --- | --- | --- |
| **Cruzain free form** | |  |
| **Replica 1** | 8.5 |  |
| **Replica 2** | 10.5 |  |
| **Replica 3** | 9.5 |  |
| **Replica 4** | 12.5 |  |
| **Average pKa** | 10.2 |  |
| **Cruzain-K777** | |  |
| **Replica 1** | 9.9 |  |
| **Replica 2** | 9.5 |  |
| **Replica 3** | 6.9 |  |
| **Replica 4** | 10.9 |  |
| **Replica 5** | 12.5 |  |
| **Replica 6** | 12.5 |  |
| **Replica 7** | 7.5 |  |
| **Replica 8** | 11.5 |  |
| **Average pKa** | 10.1 |  |
|  | **Cruzain-Substrate** |  |
| **Replica 1** | 10.5 |  |
| **Replica 2** | 11.5 |  |
| **Replica 3** | 10.9 |  |
| **Replica 4** | 11.5 |  |
| **Replica 5** | 11.8 |  |
| **Replica 6** | 20.5 |  |
| **Replica 7** | 12.5 |  |
| **Replica 8** | 6.5 |  |
| **Average pKa** | 11.9 |  |

**Table S3**. Free energy differences (ΔΔG) and pKa changes were investigated for Cz in its different forms: free, inhibitor-bound and substrate-bound. For free cruzain, four replicates were performed, based on the structural starting points called Start points 1 and 2. For the cruzain-inhibitor (Cz-K777) and cruzain-substrate complexes, eight replicates were performed for each system, based on the structures corresponding to minimum-energy structures A, B, C and D. At Start point 1, Cys25 acts as a hydrogen bond (HB) acceptor for W598 and an HB donor for W561; at Start point 2, Cys25 acts as a HB donor for W598 and an HB acceptor for W561. The structures A, B, C and D represent minima identified from the analysis of free energy graphs (FEL). Both the energy minimum E and Start point 3 were not analyzed, as they are associated with a system whose initial structure contains the presence of the CysS - /HisH + ion pair.

|  | **TI (kcal mol^-1^)** | **ΔΔG (kcal mol^-1^)** | **Δp*K*a** |
| --- | --- | --- | --- |
|  | -78.3 (0.2) |  |  |
| **Cruzain free form** | | | |
| **Replica 1** | -75.3 (0.2) | 2.9 | 2.1 |
| **Replica 2** | -74.6 (0.2) | 3.6 | 2.6 |
| **Replica 3** | -75.0 (0.2) | 3.2 | 2.3 |
| **Replica 4** | -74.7 (0.2) | 3.5 | 2.6 |
| **Total Average** | -74.9 (0.2) | 3.3 (0.1) | 2.4 (0.1) |
| **Cruzain-K777** | | | |
| **Replica 1** | -57.2 (0.1) | 21.0 | 15.3 |
| **Replica 2** | -62.5 (0.2) | 15.7 | 11.4 |
| **Replica 3** | -62.0 (0.2) | 16.2 | 11.8 |
| **Replica 4** | -58.7 (0.2) | 19.5 | 14.2 |
| **Replica 5** | -61.8 (0.2) | 16.4 | 12.0 |
| **Replica 6** | -55.2 (0.2) | 23.0 | 16.8 |
| **Replica 7** | -53.3 (0.1) | 24.9 | 18.1 |
| **Replica 8** | -58.0 (0.2) | 20.2 | 14.7 |
| **Total Average** | -58.6 (0.2) | 19.6 (1.1) | 14.2 (0.8) |
| **Cruzain-Substrate** | | | |
| **Replica 1** | -59.7 (0.1) | 18.5 | 13.5 |
| **Replica 2** | -58.9 (0.1) | 19.3 | 14.1 |
| **Replica 3** | -59.3 (0.1) | 18.9 | 13.7 |
| **Replica 4** | -59.7 (0.1) | 18.5 | 13.5 |
| **Replica 5** | -59.4 (0.1) | 18.8 | 13.7 |
| **Replica 6** | -60.8 (0.1) | 17.5 | 12.7 |
| **Replica 7** | -59.4 (0.2) | 18.8 | 13.7 |
| **Replica 8** | -58.9 (0.2) | 19.4 | 14.1 |
| **-** | - | - | - |
| **-** | - | - | - |
| **Total Average** | -59.5 (0.1) | 18.7 (0.2) | 13.6 (0.1) |
